# Mechanically driven stainless steel-initiated activation of S–H bonds to construct disulfides†

**DOI:** 10.1039/d5ra01836f

**Published:** 2025-06-12

**Authors:** Xujuan Huang, Shiming Zhang

**Affiliations:** a Key Laboratory of Biocatalysis & Chiral Drug Synthesis of Guizhou Province, Generic Drug Research Center of Guizhou Province, School of Pharmacy, Zunyi Medical University Zunyi 563000 China smzhang@zmu.edu.cn; b Key Laboratory of Basic Pharmacology of Ministry of Education, Joint International Research Laboratory of Ethnomedicine of Ministry of Education, Zunyi Medical University Zunyi 563000 China

## Abstract

Disulfides are important scaffolds in biologically active molecules and pharmaceuticals; however, their traditional synthesis method relies on costly precious metals, toxic solvents, oxidants, and harsh reaction conditions. Herein, we report a mechanochemical strategy enabling solvent-, oxidant-, catalyst-, and auxiliary abrasive-free construction of disulfides *via* stainless steel-induced S–H activation. Stainless steel nanoparticles (SS NPs) generated during ball milling facilitated efficient oxidative coupling of thiols under ambient conditions, achieving 41–99.9% yields within 30–90 minutes. The mechanistic studies confirmed the synergistic radical-mediated pathway dominated by iron species, effectively suppressing over-oxidation. This green approach eliminated environmental burdens while offering broad substrate tolerance, advancing sustainable disulfide synthesis for pharmaceutical and material applications.

## Abstract 2

We developed a mechanochemical strategy for the solvent-, oxidant-, exogenous catalyst-, and auxiliary abrasives-free synthesis of symmetrical and unsymmetrical disulfides *via* stainless steel-induced S–H activation.

## Graphical abstract

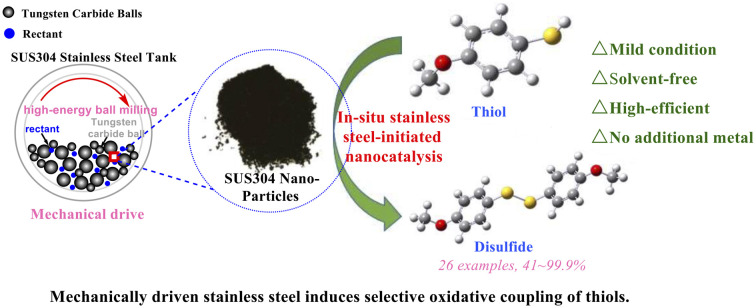

## Introduction

**Scheme 1:**
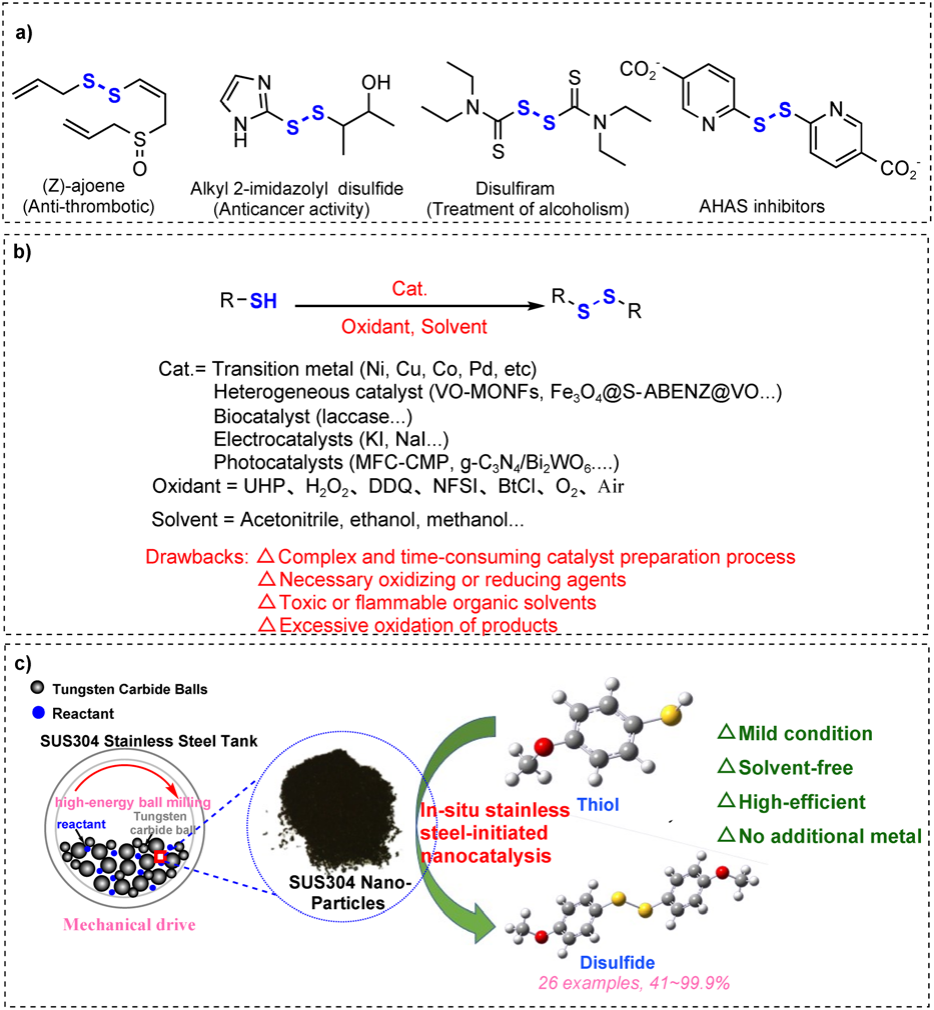
Synthesis of disulfides. (a) Examples of bioactive molecules and drugs containing S–S bonds. (b) Previous work: oxidative coupling of thiols catalyzed *via* other chemical methods. (c) This work: mechanically driven stainless steel induced selective oxidative coupling of thiols.

Disulfide bonds are privileged building blocks that are prevalent in a variety of biologically active molecules, pharmaceuticals, and natural products.^1–4^ The anti-thrombotic drug (*Z*)-ajoene found in garlic (allium) extract,^5^ alkyl 2-imidazolyl disulfide with anticancer activity,^6^ disulfiram for the treatment of chronic alcoholism,^7^ and acetohydroxyacid synthase (AHAS) inhibitors^8^ are some examples of compounds with disulfide bonds (Scheme 1a). Besides, disulfide bonds play vital roles in maintaining the secondary and tertiary structural stability of proteins.^7,9^ Furthermore, disulfide is an important reagent and intermediate for the construction of various organic compounds; disulfide is often used as a vulcanizing agent for rubber and elastomers, and by introducing disulfide bonds, rubber molecules can be cross-linked into a network structure, thereby increasing the strength and durability of rubber materials.^10,11^

Given the significance of disulfides, extensive strategies have been developed for the preparation of disulfides, such as the reductive coupling of sulfonyl compounds (*e.g.*, sodium sulfinates^12^ and sulfonyl chlorides^13^), Ni-catalyzed cross-coupling reaction of aryl(alkyl) halides with potassium sulfide,^14^ dehydrazination of arylsulfonylhydrazides using I_2_ and KOAc,^15^ and oxidative coupling of thiols. Among these synthetic methods, self-oxidative coupling of thiols is preferred as it is the most straightforward and simple way to synthesize disulfides, and thiol is an easily accessible, inexpensive substrate.^7^ The oxidative coupling of thiols to disulfides has been achieved through diverse methods (Scheme 1b), including homogeneous metal/metal oxide catalysts (Cu,^16^ Pd,^17^ Ni,^18^ and Mo^19^), heterogeneous nanomaterials (VO-MONFs,^20^ Ag–AcPy@ASMNPs,^21^ Fe_3_O_4_@MCM-41@VO-SPATB,^22^ and MCM-41 (ref. 18 and 23)), biocatalysts (laccase),^24^ electrocatalysts (potassium iodide),^25^ and photocatalysts (MFC-CMP,^26^ g-C_3_N_4_/Bi_2_WO_6_,^27^ HEO,^28^ and POM@MOF^3^). While these methods demonstrate robust catalytic activity, they are hindered by challenges such as high cost, complex and time-consuming catalyst synthesis, dependence on stoichiometric oxidants (*e.g.*, H_2_O_2_, UHP, DDQ, NFSI,^7^ and BtCl^29^), and environmental burden. Recent advancements in metal-free strategies offer sustainable alternatives for disulfide synthesis. For example, Bugde's group^30^ reported a mild, cost-effective protocol using fluorescein as a metal-free photocatalyst for visible-light-driven oxidative coupling of thiols, achieving disulfides under ambient conditions. However, this approach still necessitates thiocyanate as an oxidant, longer reaction time, and significant solvent usage. With the growing emphasis on green chemistry principles, the development of efficient, operationally simple, and eco-friendly methodologies for thiol oxidative coupling to disulfides has become a critical research priority.

Mechanochemistry,^31–34^ as a green and efficient novel technology, provides a solution for thiol oxidative coupling to build disulfides. In the last decade, mechanochemistry has been widely used in the field of organic synthesis owing to its advantages such as short reaction time, simple operation, high efficiency, and low energy consumption.^35,36^ More importantly, mechanochemistry directly utilizes mechanical energy to drive the reaction, which can efficiently promote the reaction under solvent-free conditions, change the selectivity of the products,^37^ and even achieve synthetic pathways that are not achievable *via* classical solution chemistry routes.^38^ Solvent-free reactions are not only in line with the principles of green chemistry,^39,40^ but also accelerate the reaction rate and reduce the undesirable side effects caused by solvents.^12^ In 2022, Li and Yuan^35^ successfully applied this mechanochemical method to thiol oxidative coupling for constructing disulfides. Although the method has many promising advantages, it still relies on the activation of molecular oxygen by piezoelectric materials. Green chemistry advocates minimizing the use of catalysts in chemical reactions.^41^ Bolm's group^42^ reported stainless steel-initiated chloro sulfoximidations of allenes under solvent-free conditions. The reaction does not require the addition of additional metal catalysts, but only the addition of silica as an auxiliary abrasive, which can be induced by stainless steel to produce free radicals to complete the chemical transformation.

Based on the above research, we propose a simpler and greener mechanochemical strategy for the efficient construction of disulfides by stainless steel-induced activation of S–H bonds (Scheme 1c). This study presents a solvent-free mechanochemical approach for the highly selective synthesis of disulfides under ambient conditions. Through precisely controlled mechanical activation using necessary abrasive media as intrinsic catalysts, the protocol eliminates the requirement for metal catalysts, auxiliary additives, and exogenous oxidants. The energy-transfer mechanism circumvents conventional solvent- or catalyst-mediated pathways while suppressing over-oxidation risks inherent in traditional catalytic systems.

## Results and discussion

### Reaction optimization

As shown in Table 1, to validate the mechanochemical strategy for disulfide synthesis *via* stainless steel-induced S–H activation, 4-methoxybenzenethiol (1a) was selected as a model substrate. A series of experiments were systematically conducted under different reaction conditions, including time, solvent, grinding media, ball-to-material ratio and ball milling speed. 4-Methoxybenzenethiol (0.3 mmol, 36.9 μL) was placed in a 100 mL stainless steel tank, and 63 g of tungsten carbide grinding balls were added. The reaction was conducted for 5 minutes at 500 rpm under air as the initial reaction conditions, resulting in a yield of 69.9% (entry 1). Extending the reaction time to 30 min increased the yield to 92.2% (entries 2–4), likely due to the progressive accumulation of active metal species from the grinding system. Prolonging the reaction time beyond 30 min did not further improve yields (entry 5), indicating the saturation of active species. Solvent screening reveals that polar proton-type solvents (*e.g.*, H_2_O and MeOH) exhibit significant catalytic effect (entries 6 and 7), whereas non-proton-type solvents (*e.g.*, EA, PE, and *n*-hexane) display relatively poor reactivity (entry 5 and Table S2†). Notably, solvent-free conditions delivered comparable efficiency (92.2%, entry 4), aligning with green chemistry principles. Replacing tungsten carbide balls with agate beads and zirconia balls in the agate jar led to a substantial reduction in yield, with only 19.1% and 13.2% yields, respectively (entries 9 and 10). This discrepancy arises because tungsten carbide balls, with higher density and inherent metallic components, more effectively facilitate *in situ* activation of the S–H bond within the stainless-steel jar, enabling robust mechanical energy transfer and induction of catalytic species. In contrast, agate and zirconia beads, owing to their lower density, cannot drive efficient *in situ* generation of active species critical for S–H bond activation. Additionally, optimization of the ball-to-material ratio showed a peak yield of 99.8% at 1200 : 1 (entry 11), while a lower ratio (300 : 1) resulted in poor conversion (31.3%, entry 12), likely because increasing the number of grinding balls enhances energy transfer and mass transfer efficiency and the generation of active species. Similarly, reducing the milling speed to 100 rpm halved the yield (59.5%, entry 13), underscoring the critical role of mechanical energy input in activating the catalytic system. Higher milling speeds increase the kinetic energy of the balls, promoting vigorous collisions that accelerate the generation of active species and enhance S–H bond homolysis; lower speeds decrease mechanical energy, slowing down the formation of active nanoparticles, which collectively hinders reaction progression. The optimal reaction conditions were established as follows: a ball-to-material ratio of 1200 : 1 with tungsten carbide balls, under solvent-free conditions, at a milling speed of 500 rpm for 30 minutes, affording a yield of 99.8%.

**Table 1:** Optimization of reaction conditions^a^^,^^b^

Entry	Time/min	Solvent	Milling ball	Yield (%)	Conv. (%)
1	5	Free	Tungsten carbide	69.9	75.5
2	10	Free	Tungsten carbide	75.1	83.8
3	20	Free	Tungsten carbide	89.0	98.5
4	30	Free	Tungsten carbide	92.2	98.5
5	40	Free	Tungsten carbide	88.5	98.5
6	30	H_2_O	Tungsten carbide	88.7	98.5
7	30	MeOH	Tungsten carbide	93.2	98.5
8	30	EA	Tungsten carbide	40.3	48.8
9	30	Free	Agate beads	19.1	21.3
10	30	Free	Zirconia ball	13.2	17.6
11^c^	30	Free	Tungsten carbide	99.8	99.9
12^d^	30	Free	Tungsten carbide	31.3	31.9
13^e^	30	Free	Tungsten carbide	59.5	64.8

a Reaction conditions: 1a (0.3 mmol), ten 8 mm and thirty 5 mm grinding balls (ball material ratio was 1500 : 1) added into a 100 mL ball milling jar, a milling speed of 500 rpm, reaction at room temperature for 30 min.

b Yields and conversions were determined using GC analysis with an internal standard.

c Ball material ratio was 1200 : 1.

d Ball material ratio was 300 : 1.

e Ball milling speed was 100 rpm.

### Substrate scope

**Fig. 1:**
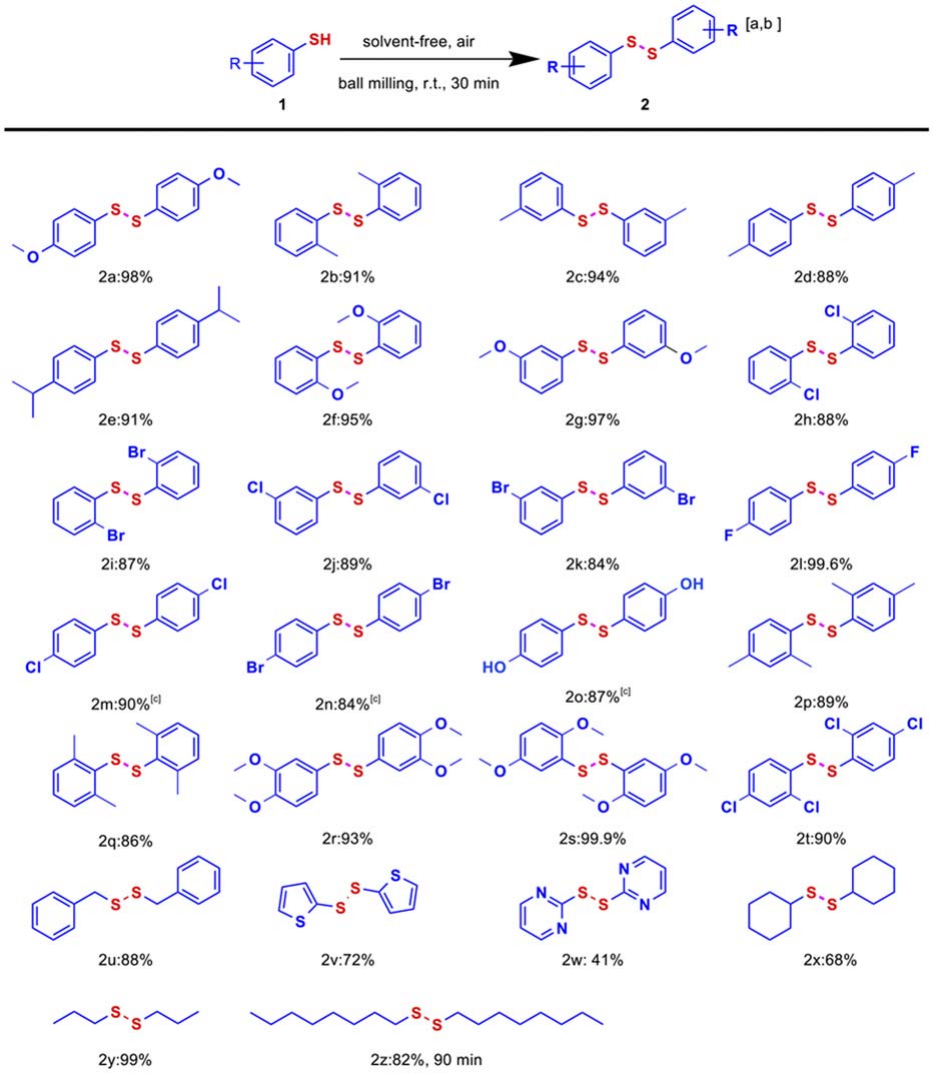
Substrate scope for the synthesis of symmetrical disulfides. Reaction condition: (a) 1 (0.3 mmol), solvent-free, eight 8 mm and twenty-four 5 mm tungsten carbide balls added into a 100 mL stainless steel jar, a milling speed of 500 rpm, reaction at room temperature for 30 min. (b) Isolated yields. (c) Solvent: 2 mL of methanol.

With optimized conditions established, the substrate scope of the disulfide construction *via* mechanochemical stainless steel-mediated S–H bond activation was systematically evaluated (Fig. 1). Aryl thiols substituted with methoxy at the *para*, *ortho*, and *meta* positions afforded disulfides 2a, 2f, and 2g in excellent yields (95–98%), demonstrating that the reaction is insensitive to substitution patterns. Substrates bearing electron-donating groups such as methyl, isopropyl, and hydroxyl consistently produced disulfides 2b–2e and 2o in high yields (87–94%). Impressively, even substrates with strongly electron-withdrawing substituents like F, Cl, and Br retained excellent reactivity, with yields ranging from 84% to 99.6% (2h–2n). Remarkably, the fluorinated derivative 2l achieved near-quantitative yield (99.6%), suggesting potential electronic or steric facilitation. Dual-substituted substrates, including those substituted with methyl, methoxy, and chlorine, also showed high reactivity, generating the corresponding disulfides 2p–2t with yields ranging from 86% to 99.9%. Moreover, benzyl- and cyclohexyl-substituted thiols also proceeded smoothly, furnishing 2u (88%) and 2x (68%), respectively; the latter highlights cyclohexane derivatives' moderate steric tolerance in the mechanochemical system. Heterocyclic thiol, including 2-thiophenethiol and 2-mercaptopyrimidine, furnished yields of 72% (2v) and 41% (2w), respectively, under standard conditions. This outcome confirms compatibility with electron-rich heterocyclic frameworks and polar heterocyclic structures. Despite 2-mercaptopyrimidine having slightly lower reactivity due to nitrogen's electron-withdrawing effect, these substrates still participated effectively in the reaction. Notably, when exploring 4-nitrobenzenethiol, 4-aminobenzenethiol, and 4-mercaptopyridine, GC-MS analysis revealed mixtures of disulfide products and thioether compounds. However, polarity and solubility issues impeded the isolation of pure disulfide products. The electron-withdrawing nitro and electron-donating amino groups, and specific heterocyclic structures of these substrates likely compromised the reaction selectivity, fostering byproduct formation. Aliphatic thiols, such as 1-propanethiol and 1-octanethiol, also exhibited good reactivity, providing yields of 99% (2y) and 82% (2z), respectively. It is worth noting that in the solvent-free mechanochemical protocol, the reaction time for 1-octanethiol was prolonged to 90 minutes to offset its relatively lower reactivity. This adjustment demonstrates the flexibility of the developed protocol, which can be readily tailored to accommodate less reactive substrates through minor modifications of the reaction conditions. This broad functional group compatibility—encompassing electron-rich, electron-deficient, multiply substituted, heterocyclic frameworks and aliphatic thiols—underscores the mechanochemical method's versatility and robustness. The uniformly high yields (41–99.9%) across structurally diverse substrates, combined with solvent-free, exogenous catalyst-free, and oxidant-free conditions, position this strategy as a sustainable and scalable alternative to conventional disulfide synthesis.

**Fig. 2:**
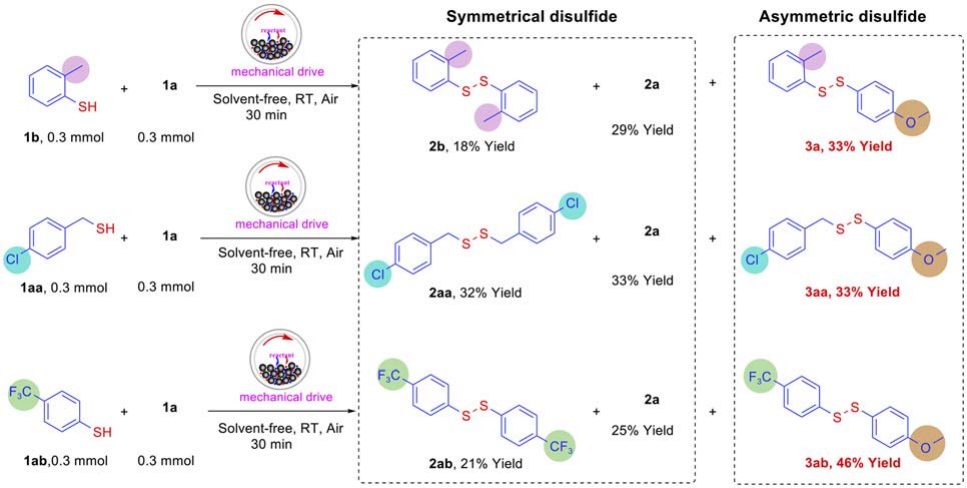
Mechanochemically driven synthesis of asymmetric disulfides.

Based on the successful construction of a symmetrical disulfide system, this study further extends the mechanochemical synthesis strategy to the field of asymmetric disulfide synthesis, as shown in Fig. 2. Cross-coupling reactions were performed between the template substrate 1a and 2-methylthiophenol (1b), 4-chlorobenzyl mercaptan (1aa), and 4-trifluoromethylthiophenol (1ab), respectively. Surprisingly, while homocoupling products of the two-component substrates were obtained, cross-coupling products were successfully synthesized with yields of 33% (3a), 33% (3aa), and 46% (3ab), respectively. Although the reaction selectivity requires further optimization, this mechanochemical method provides a green synthetic approach for asymmetric disulfide synthesis that is solvent-free, catalyst-free, and operationally simple.

### Scale-up experiments

**Fig. 3:**
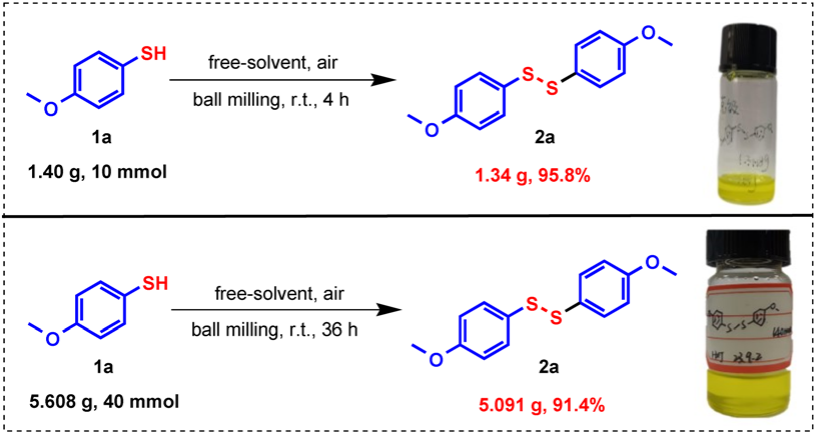
Exploration of gram-scale synthesis.

To further demonstrate the practical applicability, gram-scale synthesis of disulfide 2a were performed by scaling the model reaction to 10 mmol and 40 mmol levels (Fig. 3). During the process of scaling up the reaction, we ensure reaction homogeneity and mixing efficiency by extending the reaction time. Specifically, the reaction time is extended to 4 h for the 10 mmol scale reaction and to 36 h for the 40 mmol scale reaction. Remarkably, the process retained high efficiency, delivering 2a in 95.8% and 91.4% isolated yields, respectively. These results not only confirm the scalability of the process but also highlight the robustness of the mechanical approach in thiol oxidation coupling reactions. The near-quantitative yields under solvent- and catalyst-free conditions highlight its industrial viability, offering a scalable, cost-effective, and environmentally benign route to disulfides.

### Mechanistic investigation

To further investigate the reaction mechanism, a series of control experiments were carried out, and the results are summarized in Table 2. Firstly, in order to verify the role of tungsten carbide (WC) and stainless steel (SS) in the catalytic reaction, we replace the SS jar and WC ball with an agate jar and agate ball. The blank experiment conducted in an agate jar yielded only 16.1% (entry 1), in stark contrast to the 99.8% yield achieved using the SS jar and WC balls. This disparity highlights the pivotal role of milling material composition in driving catalytic activity. To elucidate the catalytic role of WC, control experiments were systematically conducted by introducing commercial WC powder (5–20 mg) into agate jars (entries 2–4). Although the yield gradually increased from 14.4% to 22.9% with the increase of WC addition, the marginal improvement indicated limited catalytic activity. Particle size effects were further investigated through ball-milling commercial WC powder in ethanol for 12 h (entry 5), yet the ground catalyst only achieved a 24% yield. Strikingly, replacement of agate balls with WC balls (entry 6) enhanced the yield to 51.8%, suggesting synergistic effects from trace metallic components in WC balls rather than pure WC catalysis. Subsequent investigations focused on evaluating SS components. Replacing agate balls with SS balls in agate jars (entry 7) enhanced the yield to 90.6%, while substituting the agate jar with an SS jar using agate balls (entry 8) achieved 72.3% yield. The synergistic SS jar/ball system (entry 9) demonstrated near-quantitative efficiency (98.3%), confirming dual mechanical-catalytic roles of SS components. When nanoparticles milled down from SS jars and WC balls (named SS NPs) were added to the agate jar, a yield of 96.2% was achieved (entry 10), which indicated that SS NPs were the active species.

**Table 2:** Control experiments

Entry^a^	Material of jar	Material of balls	Additive (mg)	Conv.^b^ (%)	Yield^b^ (%)
1	Agate	Agate	—	16.9	16.1
2	Agate	Agate	WC (5)	14.7	14.4
3	Agate	Agate	WC (10)	19.1	17.7
4	Agate	Agate	WC (20)	24.0	22.9
5^c^	Agate	Agate	WC (20)	24.5	24.0
6	Agate	WC	—	55.9	51.8
7	Agate	SS	—	99.6	90.6
8	SS	Agate	—	71.3	72.3
9	SS	SS	—	98.5	98.3
10	Agate	Agate	SS NPs (20)	97.0	96.2

a Reaction conditions: 1a (0.3 mmol), solvent-free, eight 8 mm and twenty-four 5 mm grinding balls added into a 100 mL grinding jar, a milling speed of 500 rpm, reaction at room temperature for 30 min.

b Yields and conversions were determined using GC analysis with an internal standard.

c WC powder was ground in an ethanol solution in an agate jar for 12 h.

To unravel the synergistic catalytic mechanism in mechanochemically driven thiol oxidative coupling, a series of characterizations of SS NPs were conducted, including powder X-ray diffraction (XRD), X-ray photoelectron spectroscopy (XPS), scanning electron microscopy (SEM), and high-resolution transmission electron microscopy (TEM).

**Fig. 4:**
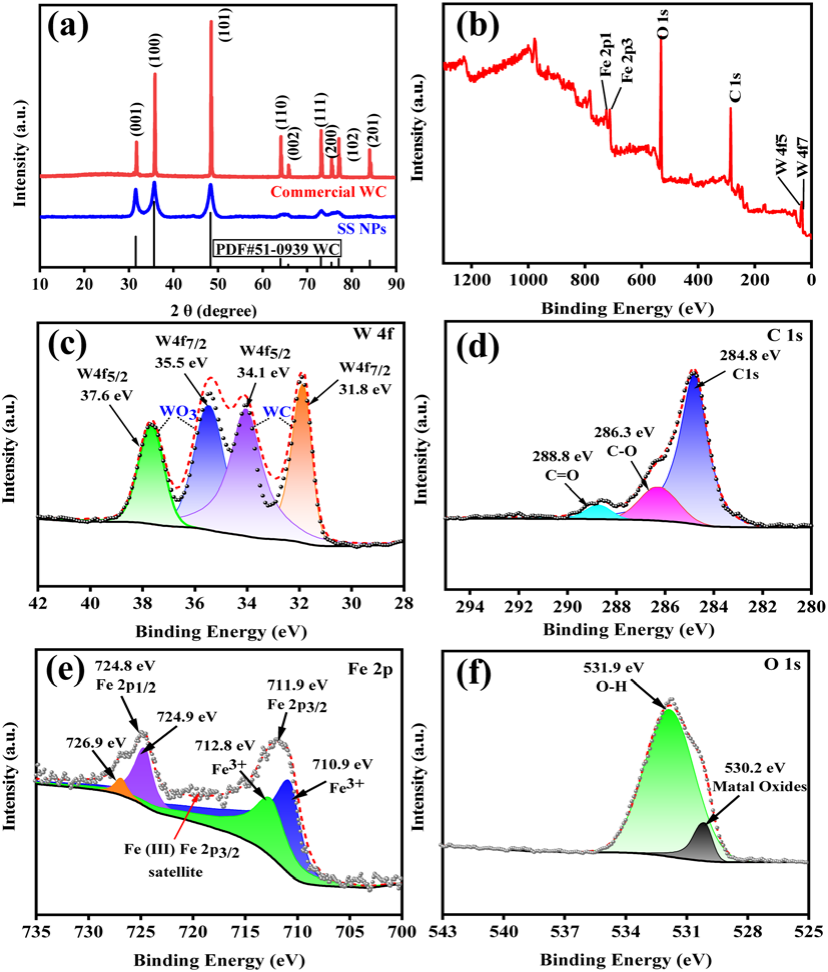
XRD and XPS characterization of SS NPs. (a) XRD images of SS NPs (blue line) and commercial WC (red line); (b) XPS survey spectrum of SS NPs; and (c) W 4f, (d) C 1s, (e) Fe 2p, and (f) O 1s high-resolution narrow spectra.

The crystal structure of SS NPs was determined by XRD (Fig. 4a). The diffraction pattern exhibited perfect consistency with hexagonal tungsten carbide^43^ (WC, space group *P-*6*m*2) from the ICDD PDF database (PDF #51-0939). While peak positions matched commercial WC powder, significant peak broadening in SS NPs indicated a reduced crystallite size. This nanoscale dimension, coupled with the previously demonstrated synergistic catalytic effects from trace metallic components in WC balls rather than pure WC, necessitated complementary XPS characterization to elucidate surface elemental composition and oxidation states. The XPS survey spectrum of SS NPs (Fig. 4b) confirms the presence of C, W, Fe, and O. The W 4f spectrum (Fig. 4c) displays two doublets: the first at 31.8 eV (W 4f_7/2_) and 34.1 eV (W 4f_5/2_) corresponding to bulk WC, and the second at 35.5 eV (W 4f_7/2_) and 37.6 eV (W 4f_5/2_) corresponding to surface WO_3_ (W^6+^). The C 1s spectrum (Fig. 4d) shows peaks at 283.6 eV (C–O bonds, possibly from organic contaminants or oxygen-containing groups) and 288.8 eV (C=O or carbonate species). The Fe 2p spectrum (Fig. 4e) indicates the introduction of iron oxides, with peaks at 710.9 eV and 712.8 eV corresponding to Fe 2p_3/2_ attributed to Fe^3+^, and the splitting peaks at 724.9 eV and 726.9 eV corresponding to Fe 2p_1/2_, with the spin–orbit splitting spacing of 14.0 eV, which is consistent with the Fe^3+^ oxidation state. There is a satellite peak at 719 eV, which may be the characteristic signal of Fe_3_O_4_. The O 1s spectrum (Fig. 4f) contains contributions from lattice oxygen in metal oxides (530.2 eV) and surface hydroxyl groups/adsorbed water (531.9 eV). The surface compositions of SS NPs comprise WC cores with partial WO_3_ oxidation layers and Fe^3+^-dominant iron oxides.

**Fig. 5:**
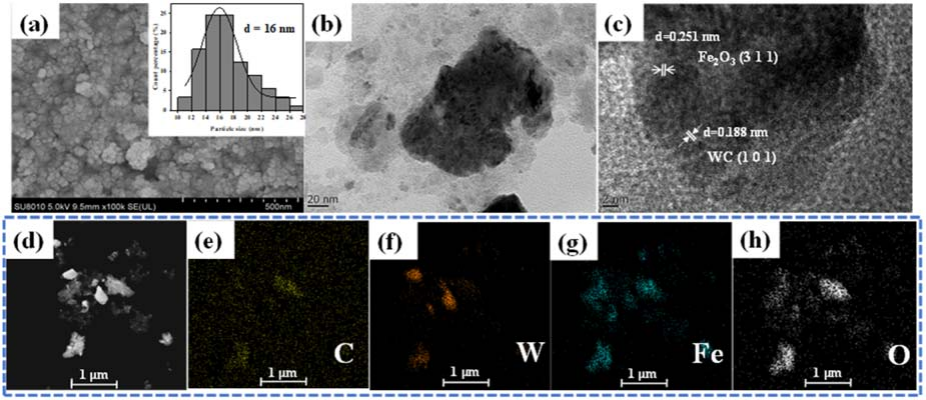
SEM and TEM characterization of SS NPs. (a) SEM images of SS NPs; (b) HRTEM images of SS NPs; (c) lattice fringe analysis of SS NPs; (d–h) electronic image and elemental distribution of EDS spectra.

The morphology of SS NPs was characterized by SEM, as shown in Fig. 5a, which reveals that SS NPs consist of uniform nanosphere agglomerates with an average diameter of approximately 16 nm. Nanoscale catalysts exhibit high specific surface area, which significantly promotes reactant adsorption and increases surface-active site density, thereby improving catalytic efficiency. Multiscale characterization of SS NPs was conducted using HRTEM and energy-dispersive X-ray spectroscopy (EDS). As shown in Fig. 5b and c, HRTEM images of the nanoplate structures exhibit well-defined lattice fringes. Fast Fourier transform (FFT) analysis (Fig. S10†) revealed distinct lattice spacings of 0.188 nm and 0.251 nm, corresponding to the (1 0 1) planes of WC and (3 1 1) planes of Fe_2_O_3_, respectively, consistent with XRD phase identification. EDS elemental mapping (Fig. 5d–h) confirmed the presence of C, W, Fe, and O, consistent with the XPS compositional analysis.

To evaluate the catalytic activity of SS NPs, TON and TOF, which serve as critical parameters for assessing the catalytic process, are indispensable. However, directly evaluating the active sites of SS NPs presents significant challenges. Given that characterization results (*e.g.*, XPS and TEM) indicated the presence of iron species (*e.g.*, Fe^3+^ and iron oxides) on the surface of SS NPs, iron and its oxides were introduced as a viable approach to assess the catalytic performance. Under the assumption that the entirety of Fe, Fe_3_O_4_ and Fe_2_O_3_ serves as catalytic active sites, relevant experiments were executed. Catalytic amounts (5 mol%) of metallic Fe, Fe_2_O_3,_ and Fe_3_O_4_ were systematically evaluated in agate jars (Table 3). The Fe_2_O_3_ and Fe_3_O_4_ systems achieved high yields of 73.2% and 91.2%, respectively, while metallic Fe exhibited moderate activity. Notably, Fe_3_O_4_ demonstrated superior catalytic efficiency (91.2%, TON = 9.3, TOF = 18.5 h^−1^), with Fe_2_O_3_ yielding TON = 7.3 and TOF = 14.7 h^−1^, and metallic Fe showing TON = 4.9 and TOF = 9.8 h^−1^. These TON and TOF values were calculated to quantify the catalytic turnover capacity within the 30 minute reaction time, which further confirms that iron species, particularly iron oxides, act as the dominant active species in this mechanochemical process.

**Table 3:** Catalytic activity of metallic Fe and its iron oxides

Entry^a^	Additive (mg)	Conversion^b^ (%)	Yield^b^ (%)	TON	TOF (h^−1^)
1	Fe powder (0.8)	49.6	43.6	4.9	9.8
2	Fe_3_O_4_ (3.5)	93.1	91.2	9.3	18.5
3	Fe_2_O_3_ (2.4)	88.1	73.2	7.3	14.7

a Reaction conditions: 1a (0.3 mmol), solvent-free, 5 mol% addictive, eight 8 mm and twenty-four 5 mm agate balls added into a 100 mL agate pot, a milling speed of 500 rpm, reaction at room temperature for 30 min.

b Yields and conversions were determined by GC analysis with an internal standard.

**Fig. 6:**
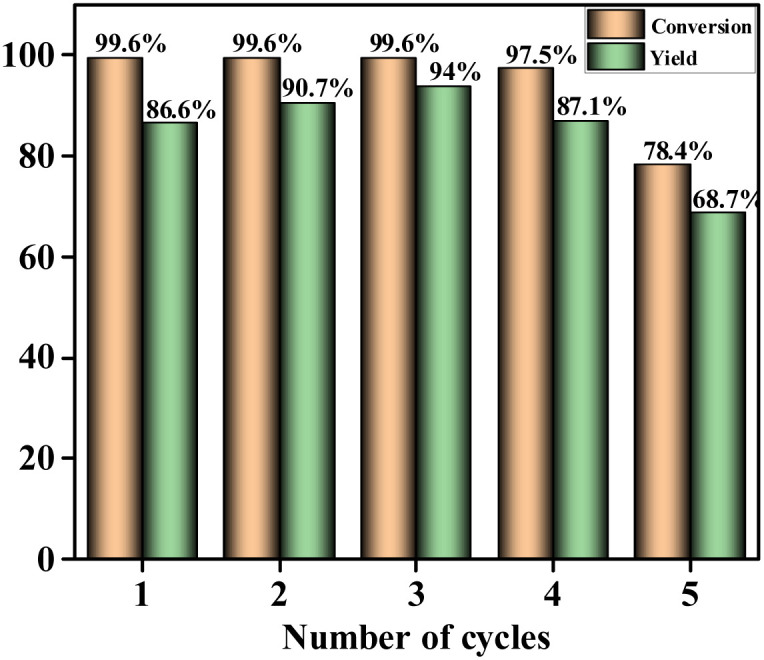
Recycling experiments of SS NPs in an agate jar.

To further explore the application potential of SS NPs, cyclic utilization experiments were conducted. SS NPs generated by grinding tungsten carbide balls in a stainless-steel tank were recycled. Then, 5 mg of SS NPs served as the catalyst in an agate–tank reaction system, reacting at room temperature for 30 min. Afterward, the solid particles were washed with ethyl acetate, recovered, dried, and reused until the yield decreased significantly. As shown in Fig. 6, SS NPs exhibit excellent recycling performance: the yield gradually increased in the first three cycles but decreased with increasing cycles, dropping to 68.7% in the fifth cycle. The yield initially increases and then decreases, which is likely attributed to changes in catalyst particle size and active sites. In the initial cycles, smaller nanoparticle size increases the specific surface area and exposes more active sites, enhancing the catalytic efficiency. With increasing cycles, however, nanoparticle aggregation reduces the number of accessible active sites, leading to a decline in yield.

**Fig. 7:**
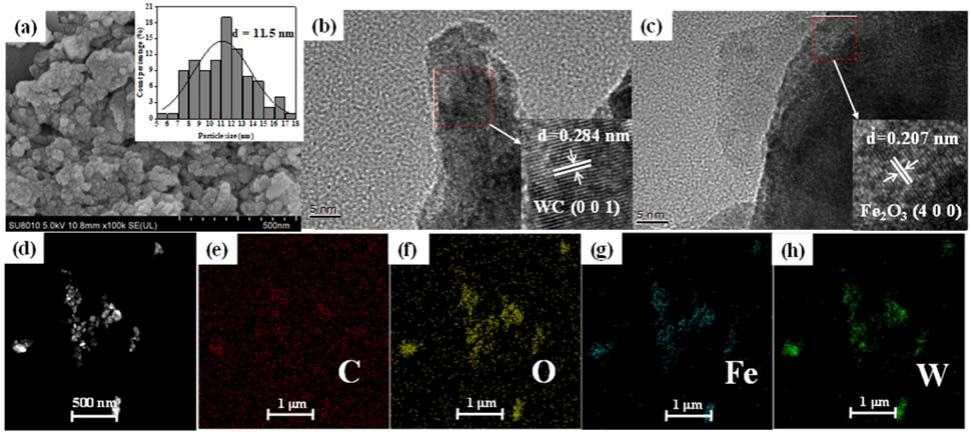
SEM and TEM characterization of SS NPs after 5 cycles. (a) SEM images of SS NPs; (b and c) HRTEM images and lattice fringe analysis of SS NPs; (d–h) electronic image and elemental distribution of EDS spectra.

To investigate the structural stability of SS NPs before and after the reaction, SEM and TEM characterizations were performed on the samples after 5 cycles of use, as shown in Fig. 7. The SEM characterization (Fig. 7a) revealed that after 5 cycles, SS NPs exhibited a slight increase in agglomeration, but the average particle size decreased from the initial 16 nm to 11.5 nm. Further TEM analysis (Fig. 7b and c) indicated that the phase composition of SS NPs remained unchanged before and after the reaction, with the main components being WC and Fe_2_O_3_. EDS elemental mapping (Fig. 7d–h) showed no significant variation in the uniform distribution of elements before and after the reaction. These characterization results collectively confirm that the catalyst maintains good structural stability before and after the reaction.

To probe the involvement of radical pathways in mechanochemically driven thiol oxidative coupling, radical scavenging experiments were conducted using the radical scavengers 2,2,6,6-tetramethylpiperidinyl-1-oxide (TEMPO) and 2,6-di-*tert*-butyl-4-methylphenol (BHT) under standard conditions (Table 4). The addition of TEMPO significantly inhibits the formation of the target product: in the standard reaction system (entry 1), complete substrate conversion was observed with about 40% yield reduction to 59.7% (*vs.* 99.8% control); in agate/SS systems (entry 3, 86.8% yield), TEMPO addition (entry 4) caused a 26.8% yield drop to 60%, while in Fe_3_O_4_-catalyzed systems (entry 5, 98.1% yield), TEMPO induced a more pronounced 44.0% yield decrease to 54.1% (entry 6). This inhibition shows a free radical reaction mechanism.^44^ Moreover, HRMS analysis confirmed radical intermediates through detected adducts^45^ (*m*/*z* = 318.1509, calc. 318.1504) (see Fig. S12†). In contrast, BHT showed minimal yield variation (entry 2), suggesting selective radical scavenging by TEMPO. Intriguingly, TEMPO exhibited dual functionality: as a sole catalyst^46^ (entry 7), it achieved full conversion but only 56% yield, with GC-MS identification of *S*-(4-methoxyphenyl) 4-methoxybenzenesulfonothioate byproducts (*m*/*z* = 311.2) (see Fig. S13†), revealing its concurrent roles in radical scavenging and oxidative side reactions, which collectively explain the residual yields in inhibition experiments.

**Table 4:** Free radical trapping experiments

Entry^a^	Material of jar	Material of balls	Radical scavenger/additive	Conversion^b^ (%)	Yield^b^ (%)
1	SS	WC	TEMPO (2 equiv.)	98.4	59.7
2	SS	WC	BHT (2 equiv.)	85.5	85.2
3	Agate	SS	—	97.3	86.8
4	Agate	SS	TEMPO (2 equiv.)	99.6	60.0
5	Agate	Agate	Fe_3_O_4_ (15%)	98.3	98.1
6	Agate	Agate	Fe_3_O_4_ (15%) + TEMPO (2 equiv.)	99.6	54.1
7	Agate	Agate	TEMPO (2 equiv.)	99.6	56.0

a Reaction conditions: 1a (0.3 mmol), solvent-free, eight 8 mm and twenty-four 5 mm grinding balls added into a 100 mL grinding jar, a milling speed of 500 rpm, reaction at room temperature for 30 min.

b Yields and conversions were determined using GC analysis with an internal standard.

**Fig. 8:**
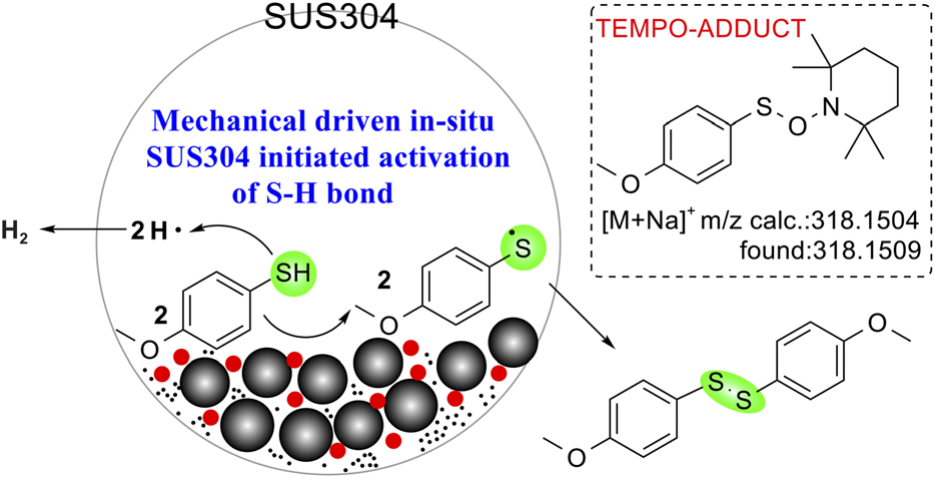
Possible reaction mechanism.

Based on all of the control experimental results, characterization data, and previous reports, a plausible tentative mechanism for the selective formation of disulfide is proposed in Fig. 8. Initially, ball milling generates SS NPs and tungsten carbide-derived species, exposing active iron oxides and WC surfaces. Mechanical energy induces homolytic cleavage of S–H bonds in thiols, forming thiyl radicals (RS˙) and hydrogen radicals (H˙) without the need for an additional oxidant. Fe^3+^ facilitates electron transfer, stabilizing radicals and promoting their coupling to form disulfides (RSSR). Meanwhile, the metal element within SS NPs catalyzes the production of hydrogen.^47,48^ WC cores and oxidized WO_3_ layers enhance electron mobility, while iron oxides provide active sites for radical stabilization, collectively suppressing over-oxidation.

### Comparison of the method

Compared with traditional protocols for synthesizing disulfides (Table 5, entries 1–7), which typically rely on elaborate catalyst preparation, stoichiometric oxidants, and substantial amounts of toxic organic solvents, the mechanochemical approach offers distinct advantages. Moreover, under certain catalysts like TBAI (entry 8), ^n^Bu_4_NBF_4_ (entry 9), [Mo]_2_ (entry 10), and (BuSe)_2_ (entry 11), traditional methods often suffer from over-oxidation, yielding by-products such as sulfonic esters, sulfonates, and thiosulfonates. In contrast, our mechanochemical strategy obviates the need for complex catalyst synthesis or external oxidants. By directly utilizing stainless steel to induce *in situ* activation of S–H bonds, it enables the formation of disulfides with remarkable benefits—simple operation, environmental benignity, and high efficiency. Notably, this method achieves a disulfide yield of 99.9% (entry 12), outperforming many traditional approaches that struggle with lower yields (*e.g.*, 84% in entry 4 and 80% in entry 5) or produce unwanted by-products, thereby highlighting its superiority in both efficiency and selectivity.

**Table 5:** Comparison of traditional protocols for the synthesis of disulfide from thiol

Entry	Catalyst/oxidant/solvent	Time	Product (yield)	Ref.
1	Sr-MOF/O_2_/MeOH	6 h	Disulfide (99)	49
2	BrCCl_3_ and NaOH/O_2_/water	12 h	Disulfide (91)	50
3	CoFe_2_O_4_@SiO_2_@CPTMS@VO(salophen-OH)/UHP/EtOH	50 min	Disulfide (90)	51
4	BaMnO_4_/BaMnO_4_/PEG-200	4 h	Disulfide (84)	52
5	GR–CdS–(Co–Pi) ternary photocatalyst/—/MeCN	5 h	Disulfide (80)	53
6	KIO_3_/—/H_2_O	40 min	Disulfide (96)	54
7	DDQ/DDQ/MeCN	4.5 h	Disulfide (92)	55
8	TBAI/^*t*^BuONO/Toluene	8 h	Thiosulfonates (86)	56
9	^*n*^Bu_4_NBF_4_/—/CH_2_Cl_2_	20 h	Sulfinic esters (87)	57
10	[Mo]_2_/UHP/EtOH	24 h	Sulfonate (98)	58
11	Bu(Se)_2_/H_2_O_2_/CH_3_CN	24 h	Thiosulfonates (75)	59
12	SS NPs/—/—	30 min	Disulfide (99.9)	This work

## Conclusions

In summary, we developed a mechanochemical strategy for the solvent-free, oxidant-free, exogenous catalyst-free, and auxiliary abrasive-free synthesis of symmetrical and unsymmetrical disulfides *via* stainless steel-induced S–H activation. The method leverages SS NPs generated during ball milling as active species to drive efficient oxidative coupling of thiols under ambient conditions, which has a good yield and substrate universality. Gram-scale reactions retained high efficiency, demonstrating industrial viability. Mechanistic studies have shown that the synergistic effects between iron species and WC nanoparticles were identified as critical for catalytic activity, with radical-mediated pathways dominating the reaction. This approach, which eliminates toxic solvents, external oxidants, and precious metal catalysts, aligns with the principles of sustainable synthesis and establishes a robust platform for scalable disulfide synthesis in pharmaceutical and materials science applications.

## Experimental

### Reagents and solvents

4-Methoxybenzenethiol (97%) was obtained from Shanghai Bide Pharmaceutical Technology Co., Ltd. Other aliphatic or aromatic thiol substrates were obtained from Shanghai Bide Pharmaceutical Technology Co., Ltd or Beijing InnoChem Science & Technology Co., Ltd. Ethyl acetate (EA), acetonitrile (MeCN), methanol (MeOH), dichloromethane (DCM), *n*-hexane, acetone, petroleum ether (PE), *N*,*N*-dimethylformamide (DMF) and other solvents were acquired from Beijing InnoChem Science & Technology Co., Ltd. Tungsten carbide powder (<10 μm, 99.9%) was commercially purchased.

## Experimental procedures

4-Methoxybenzenethiol (1a, 0.3 mmol, 36.9 μL) was placed in a 100 mL stainless steel tank, and 63 g of tungsten carbide grinding balls were added. Then the tank was closed, and the reaction was performed by using a high-energy planetary ball mill for 30 min at 500 rpm. After the reaction was completed, 10 mL of ethyl acetate containing 20 mM 4-nitrotoluene internal standard was added to the stainless steel tank to fully extract the sample. The organic phase was dried with anhydrous sodium sulfate and filtered through a 0.22 μm organic filter membrane. Then, gas chromatography was used to analyze the yield and conversion of the sample.

## Author contributions

Shiming Zhang: supervision, conceptualization, methodology, writing-review & editing, funding acquisition. Xujuan Huang: conceptualization, methodology, software, validation, formal analysis, investigation, writing-original draft.

## Conflicts of interest

There are no conflicts to declare.

## Supplementary Material

RA-015-D5RA01836F-s001

## Data Availability

All data supporting the findings of this study are available within the paper and its ESI.†
