# Supplementary material for: Mechanically driven stainless steel-initiated activation of S–H bonds to construct disulfides
Source: RSC Adv. 2025 Jun 12;15(24):19296–304. doi: 10.1039/d5ra01836f (PMC12159594; doi:10.1039/d5ra01836f)
Supplement: RA-015-D5RA01836F-s001 [file RA-015-D5RA01836F-s001.pdf]

Supplementary Information (SI) for RSC Advances.
This journal is © The Royal Society of Chemistry 2025

Supporting Information

Mechanically driven stainless steel-initiated activation of S-H bonds to construct disulfides

Xujuan Huang [1,2], Shiming Zhang [1,2,*]

[1] *Key Laboratory of Biocatalysis & Chiral Drug Synthesis of Guizhou Province, Generic Drug Research Center of Guizhou Province, School of Pharmacy, Zunyi Medical University, Zunyi, 563000, China.*

[2] *Key Laboratory of Basic Pharmacology of Ministry of Education and, Joint International Research Laboratory of Ethnomedicine of Ministry of Education, Zunyi Medical University, Zunyi, 563000, China.*

.

***Corresponding author:**

Shiming Zhang, E-mail: smzhang@zmu.edu.cn

Table of Contents

**Experimental details**	**p.**
**Figure S1** The standard curve of substrate (left) and product (right)	4
**Figure S2** GC chromatography of reaction solution with internal standard. Analysis method: HP-5 (30 m × 0.32 mm × 0.25 μm, the initial temperature was 90 °C for 0 min, then it was heated to 100 °C at a rate of 2 °C/min for 2 min, and then heated to 250 °C at a rate of 10 °C/min for 2 min).	5
**Table S1** The reaction time screening.	6
**Figure S3** The reaction time screening.	6
**Table S2** The reaction solvent screening.	7
**Figure S4** The reaction solvent screening.	7
**Table S3** Screening of ball mill media types.	8
**Figure S5** Screening of ball mill media types.	8
**Table S4** Ball material ratio screening.	9
**Figure S6** Ball material ratio screening.	9
**Table S5** Ball mill speed screening.	10
**Figure S7** Ball mill speed screening.	10
**Figure S8** SEM image and particle size distribution of SS NPs.	11
**Figure S9** SEM image and particle size distribution of SS NPs after reaction.	11
**Figure S10** SS NPs (a, b, d) HRTEM images and lattice fringe analysis at greater magnifications; (c, e, f) Fast Fourier Transform plots (FFT); (g) Electronic image and elemental content distribution of EDS spectra.	12
**Figure S11** SEM and TEM characterization of SS NPs after 5 cycles. (a) SEM images of SS NPs; (b~c) HRTEM images and lattice fringe analysis of SS NPs; (d~h) Electronic image and elemental distribution of EDS spectra.	12
**Figure S12** The HRMS of tempo-adduct: calcd. for $C_{16}H_{25}NO_2NaS$ $[M + Na]^+$ 318.1504, found 318.1509.	13

**Figure S13** GC-MS spectra of Tempo hyperoxidation of thiols to S-(4-methoxyphenyl) 4-methoxybenzenesulfonothioate byproducts.	14
**Spectroscopic Data**	15
**^{1}H and ^{13}C NMR Spectra**	22

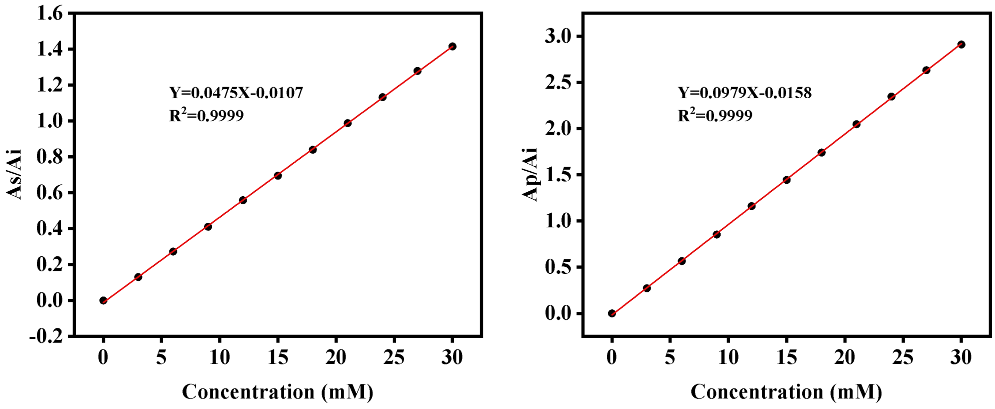

Figure S1 The standard curve of substrate (left) and product (right)

Use the internal standard method for quantitative analysis of reactants to minimize errors. A 20 mM solution of 2-nitrotoluene was employed as the internal standard to establish standard curves. A series of standard solutions with known concentrations (3 mM, 6 mM, 9 mM, 12 mM, 15 mM, 18 mM, 21 mM, 24 mM, 27 mM, and 30 mM) of the substrate 4-methoxyphenylthiophenol (**1a**) and the product bis(4-methoxyphenyl)disulfide (**2a**) were prepared. The standard curve is drawn by using the linear relationship between the peak area ratio of **1a (or 2a)** and the internal standard and the concentration. Finally, **1a (or 2a)** internal standard solutions of different concentrations were analysis by gas chromatography. Analysis method: HP-5 (30 m × 0.32 mm × 0.25 μm, the initial temperature was 90 °C for 0 min, then it was heated to 100 °C at a rate of 2 °C/min for 2 min, and then heated to 250 °C at a rate of 10 °C/min for 2 min).

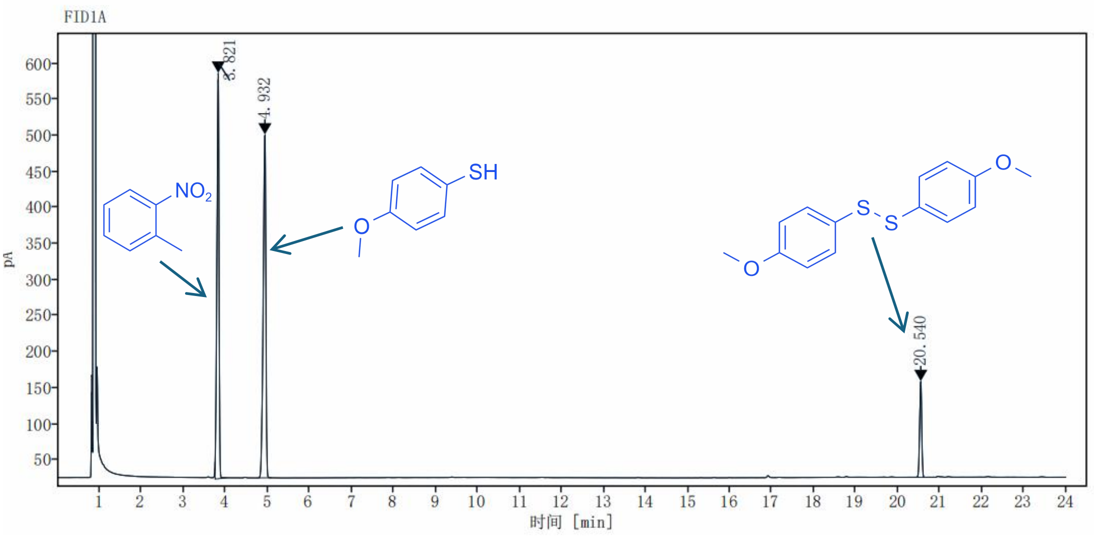

信号: FID1A

保留时间 [min]	类型	峰宽 [min]	峰面积	峰高	峰面积%	名称
3.821	MM m	0.2708	1990.7952	562.5773	43.8088	
4.932	MM m	0.3070	2094.0354	475.8050	46.0807	
20.540	BB	0.2344	459.4528	133.0826	10.1106	

Figure S2 GC chromatography of reaction solution with internal standard. Analysis method: HP-5 (30 m × 0.32 mm × 0.25 μm, the initial temperature was 90 ^{o}C for 0 min, then it was heated to 100 ^{o}C at a rate of 2 ^{o}C/min for 2 min, and then heated to 250 ^{o}C at a rate of 10 ^{o}C/min for 2 min).

Table S1 The reaction time screening.

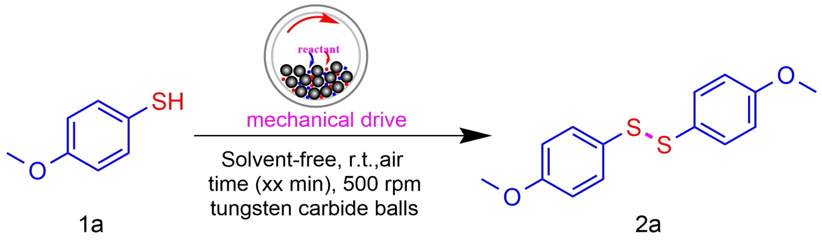

Entry[a]	Time (min)	Conversion (%)-1a[b]]	Yield (%)-2a[b
**1**	5	75.5	69.9
**2**	10	83.8	75.1
**3**	20	98.5	89.0
**4**	**30**	**98.5**	**92.2**
**5**	40	98.5	88.5
**6**	50	98.5	90.9
**7**	60	98.5	90.1
**8**	120	98.5	87.1
**9**	240	98.5	92.1

[a] Reaction conditions: **1a** (0.3 mmol), solvent-free, ten 8 mm and thirty 5 mm tungsten carbide balls were added into a 100 mL stainless steel jar, and the milling speed was 500 rpm, reaction at room temperature for xx min.
[b] Yields and conversions were determined by GC analysis with an internal standard.

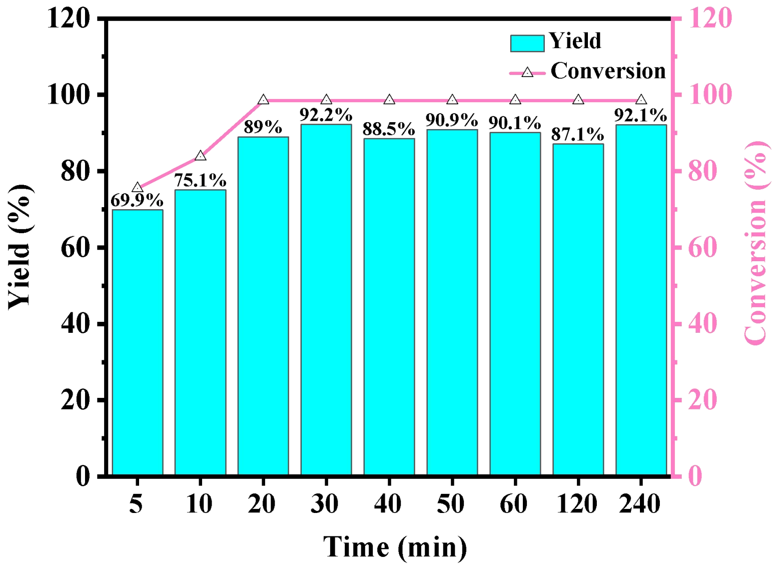

Figure S3 The reaction time screening.

Table S2 The reaction solvent screening.

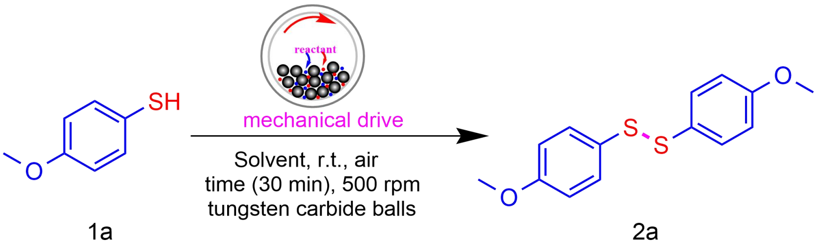

Entry[a]	Solvent (mL)	Conversion (%)-1a[b]	Yield (%)-2a[b]
**1**	**free**	**98.5**	**92.2**
**2**	H_2O (2)	98.5	88.7
**3**	EA (2)	48.8	40.3
**4**	MeCN (2)	98.5	88.9
**5**	MeOH (2)	98.5	93.2
**6**	DCM (2)	55.4	45.6
**7**	n-hexane (2)	83.5	79.8
**8**	acetone (2)	80.3	77.4
**9**	PE (2)	70.5	58.6
**10**	DMF (2)	98.5	83.4

[a] Reaction conditions: **1a** (0.3 mmol), solvent was xx (2 mL), ten 8 mm and thirty 5 mm tungsten carbide balls were added into a 100 mL stainless steel jar, and the milling speed was 500 rpm, reaction at room temperature for 30 min.

[b] Yields and conversions were determined by GC analysis with an internal standard.

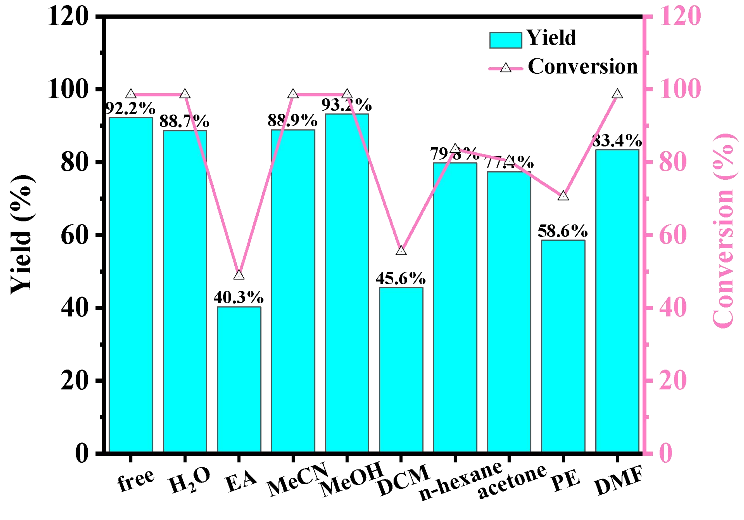

Figure S4 The reaction solvent screening.

Table S3 Screening of ball mill media types.

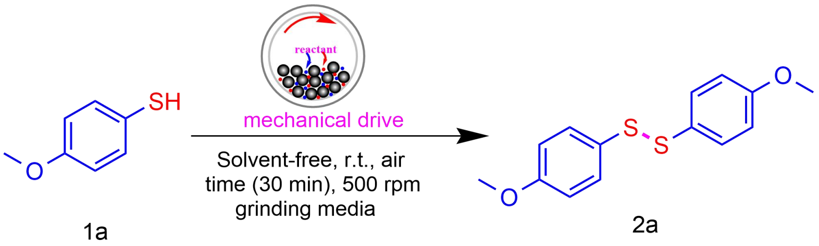

Entry[a]	Grinding Ball	Conversion (%)-1a[b]	Yield (%)-2a[b]
**1**	Tungsten carbide balls	98.5	92.2
**2**[c]	Agate beads	21.3	19.1
**3**[c]	Zirconia balls	17.6	13.2

[a] Reaction conditions: **1a** (0.3 mmol), solvent-free, ten 8 mm and thirty 5 mm different grinding balls were added into a 100 mL stainless steel jar, and the milling speed was 500 rpm, reaction at room temperature for 30 min.

[b] Yields and conversions were determined by GC analysis with an internal standard.

[c] The ball mill jar used is an agate jar.

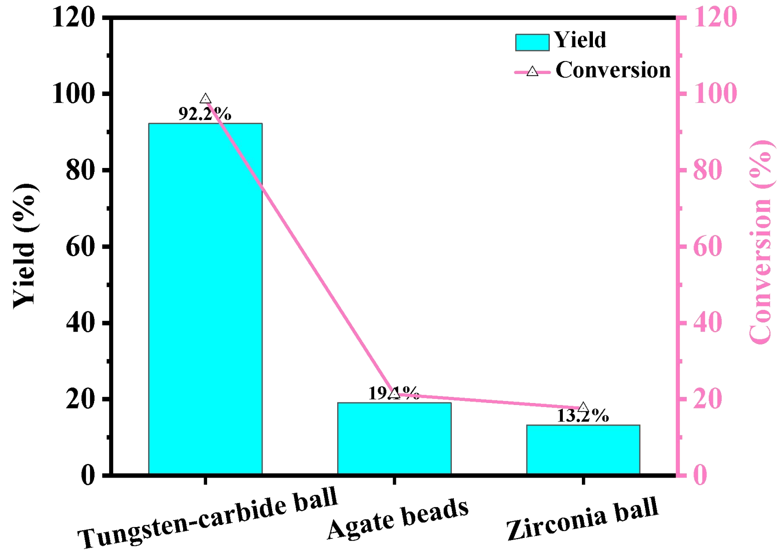

Figure S5 Screening of ball mill media types.

Table S4 Ball material ratio screening.

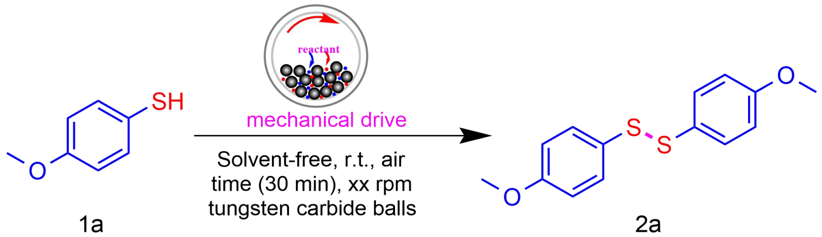

1a 2a

Entry[a]	Ball Material Ratio	Conversion (%)-1a[b]]	Yield (%)-2a[b
**1**	300： 1	31.9	31.3
**2**	600： 1	67.1	65.5
**3**	900： 1	96.8	95.3
**4**	1200： 1	99.9	99.8
**5**	1500： 1	97.8	97.4

[a] Reaction conditions: **1a** (0.3 mmol), solvent-free, x 8 mm and x 5 mm tungsten carbide balls were added into a 100 mL stainless steel jar, and the milling speed was 500 rpm, reaction at room temperature for 30 min.

[b] Yields and conversions were determined by GC analysis with an internal standard.

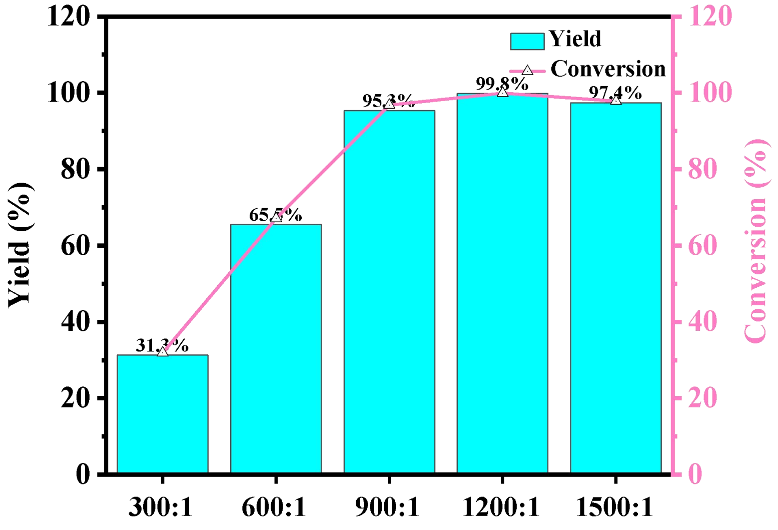

Figure S6 Ball material ratio screening.

Table S5 Ball mill speed screening.

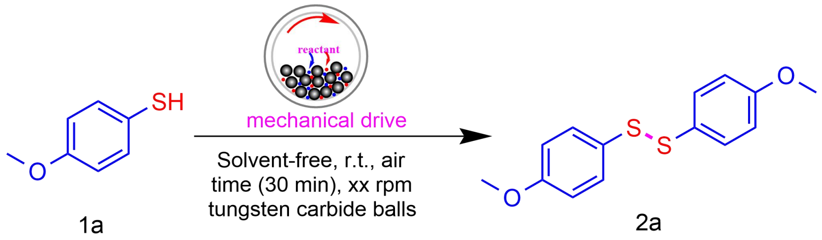

Entry[a]	Speed (rpm)	Conversion (%)-1a[b]]	Yield (%)-2a[b
**1**	100	64.8	59.5
**2**	200	72.1	61.6
**3**	300	85.4	86.3
**4**	400	99.0	97.0
**5**	500	99.9	99.8

[a] Reaction conditions: **1a** (0.3 mmol), solvent-free, eight 8 mm and twenty-four 5 mm tungsten carbide balls were added into a 100 mL stainless steel jar, and the milling speed was xx rpm, reaction at room temperature for 30 min.

[b] Yields and conversions were determined by GC analysis with an internal standard.

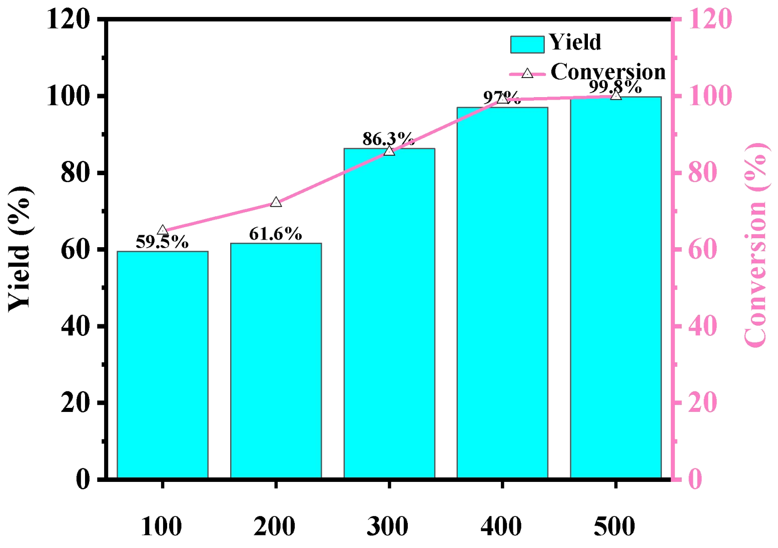

Figure S7 Ball mill speed screening.

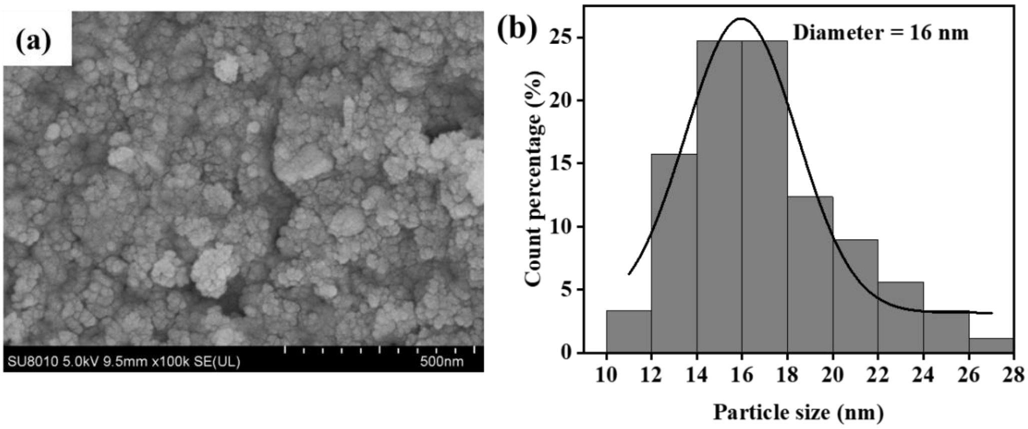

Figure S8 SEM image and particle size distribution of SS NPs before reaction.

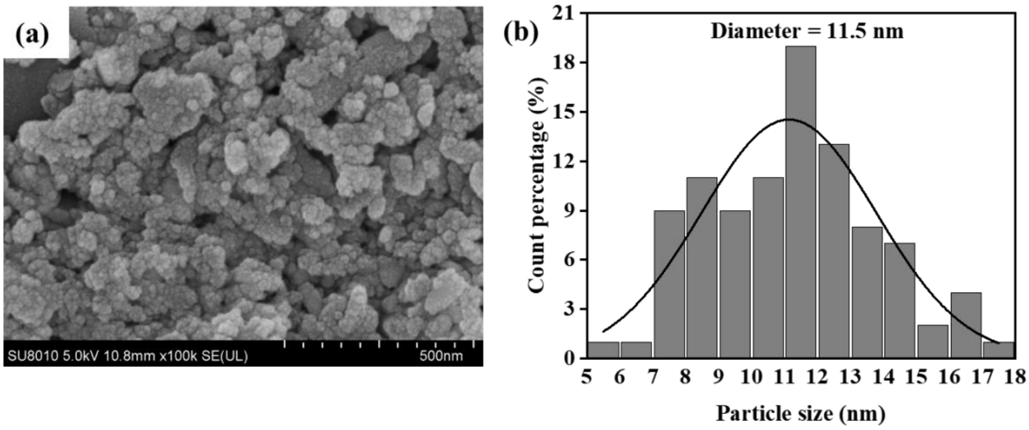

Figure S9 SEM image and particle size distribution of SS NPs after reaction.

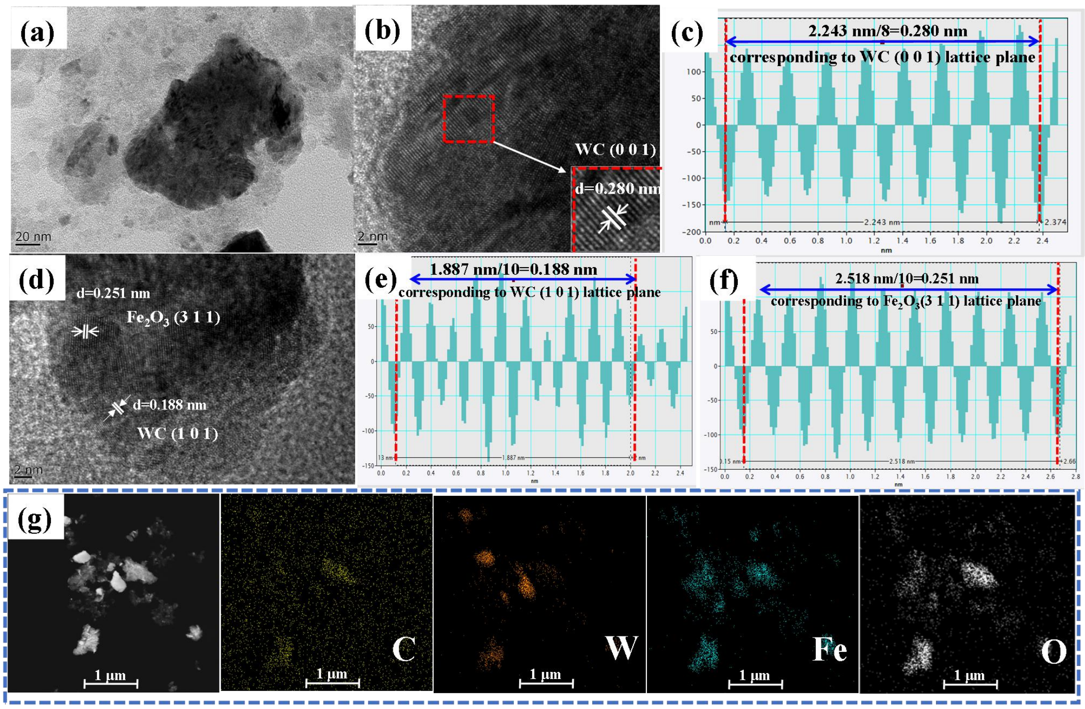

Figure S10 SS NPs (a, b, d) HRTEM images and lattice fringe analysis at greater magnifications; (c, e, f) Fast Fourier Transform plots (FFT); (g) Electronic image and elemental content distribution of EDS spectra.

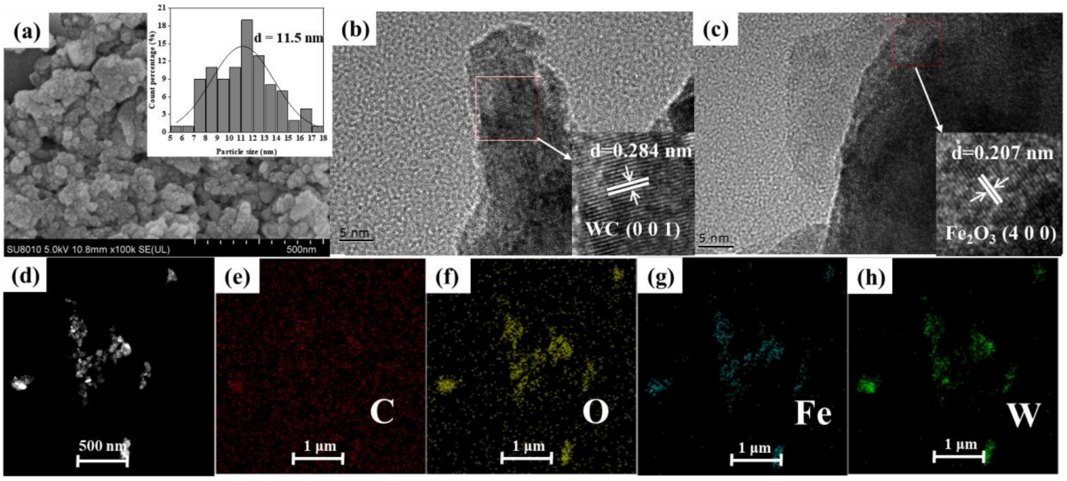

Figure S11 SEM and TEM characterization of SS NPs after 5 cycles. (a) SEM images of SS NPs; (b~c) HRTEM images and lattice fringe analysis of SS NPs; (d~h) Electronic image and elemental distribution of EDS spectra.

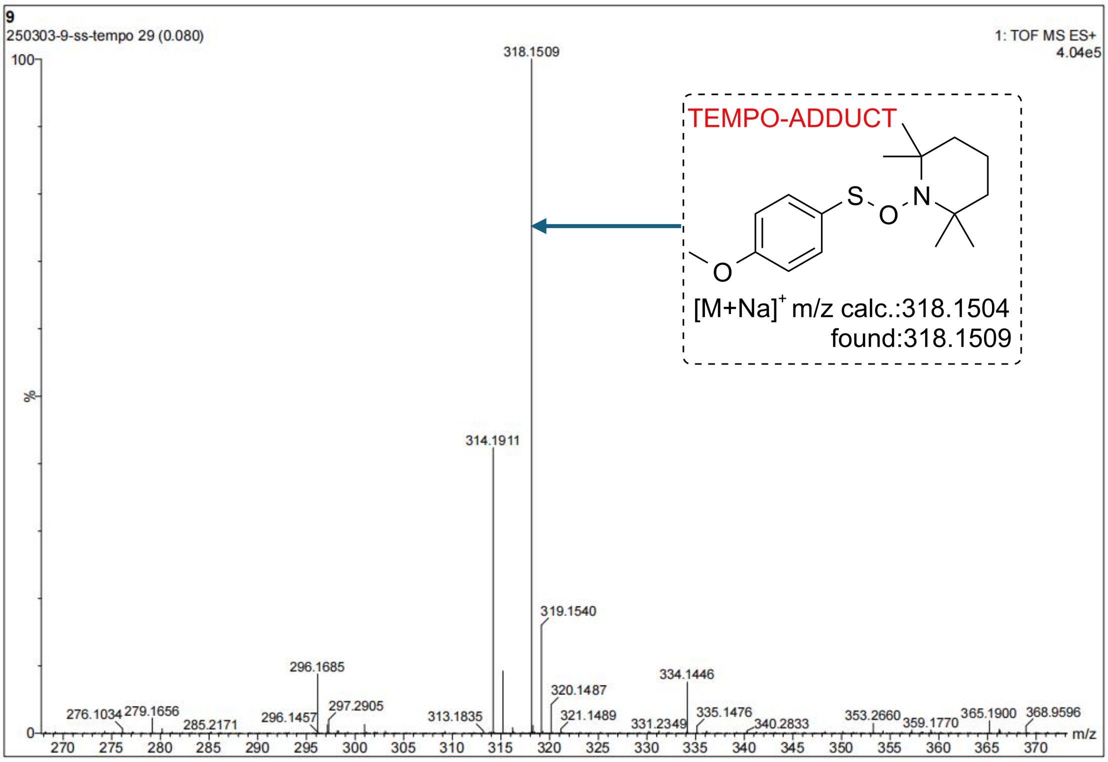

Figure S12 The HRMS of tempo-adduct: calcd. for $C_{16}H_{25}NO_2NaS$ $[M + Na]^+$ 318.1504, found 318.1509.

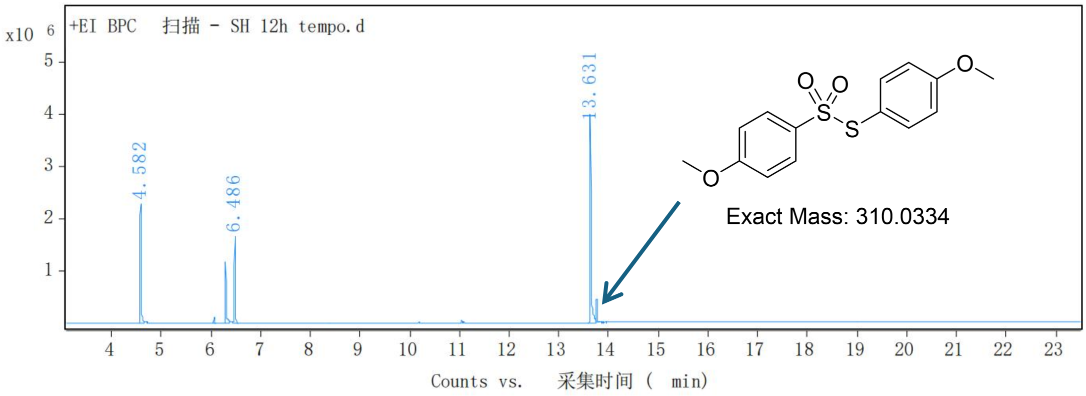

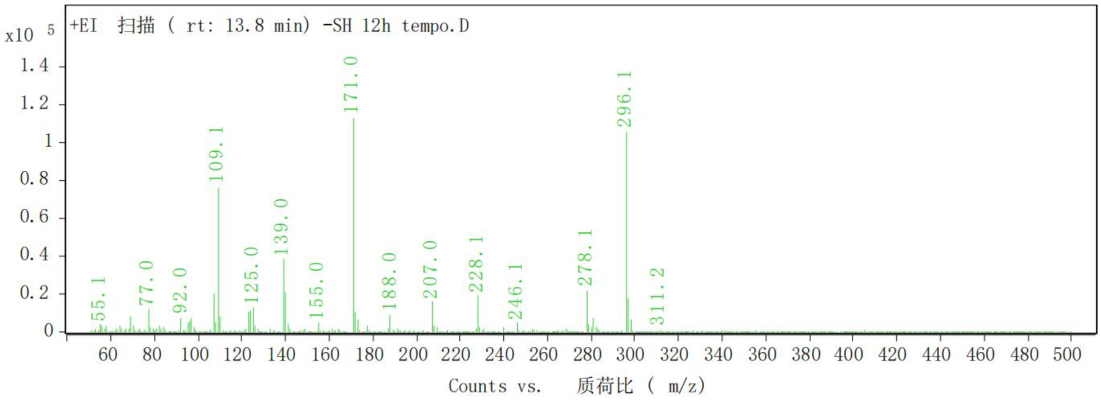

Figure S13 GC-MS spectra of Tempo hyperoxidation of thiols to S-(4-methoxyphenyl) 4-methoxybenzenesulfonothioate byproducts.

Spectroscopic Data

1,2-bis(4-methoxyphenyl)disulfane (2a): CAS Number 5335-87-5.

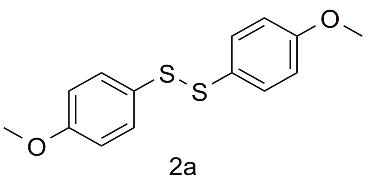

2a

Following the general procedure, the title compound was isolated as a yellow solid (Yield 98%, 41.0 mg). **^{1}H-NMR** (400 MHz, $CDCl_3$)：δ 7.40 (d, *J* = 8.8 Hz, 4H), 6.84 (d, *J* = 8.8 Hz, 4H), 3.80 (s, 6H). **^{13}C-NMR** (101 MHz, $CDCl_3$)：δ 160.0, 132.8, 128.5, 114.7, 55.5.

1,2-di-o-tolyldisulfane (2b): CAS Number 4032-80-8.

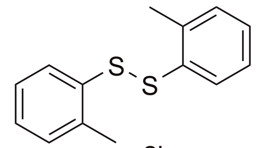

2b

Following the general procedure, the title compound was isolated as a yellow brown solid (Yield 91%, 33.6 mg). **^{1}H-NMR** (400 MHz, $CDCl_3$)：δ 7.53 (dd, *J* = 5.6 Hz, 2H), 7.19-7.12 (m, 6H), 2.45 (s, 6H). **^{13}C NMR** (101 MHz, $CDCl_3$)：δ 137.3, 135.5, 130.4, 128.5, 127.4, 126.8, 20.1.

1,2-di-m-tolyldisulfane (2c): CAS Number 20333-41-9.

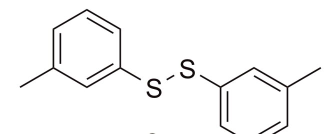

2c

Following the general procedure, the title compound was isolated as a yellow solid (Yield 94%, 34.6 mg). **^{1}H NMR** (400 MHz, $CDCl_3$)：δ 7.33–7.31 (m, 4H), 7.22-7.18 (m, 2H), 7.06–7.04 (m, 2H), 2.34 (s, 6H). **^{13}C NMR** (101 MHz, $CDCl_3$)：δ 139.1, 137.0, 129.0, 128.1, 128.0, 124.6, 21.5.

1,2-di-p-tolyldisulfane (2d): CAS Number 103-19-5.

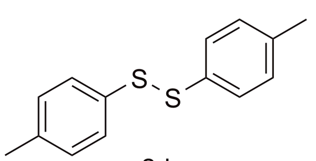

2d

Following the general procedure, the title compound was isolated as a yellow solid (Yield 88%, 32.6 mg). **^{1}H-NMR** (400 MHz, $CDCl_3$)：δ 7.40 (d, *J* = 8.4 Hz, 4H), 7.12 (d, *J* = 8.0 Hz, 4H), 2.34 (s, 6H). **^{13}C-NMR** (101 MHz, $CDCl_3$)：δ 137.5, 134.0, 129.9, 128.6, 21.2.

1,2-bis(4-isopropylphenyl)disulfane (2e): CAS Number 622407-64-1.

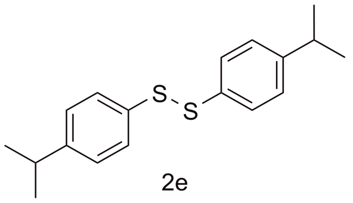

2e

Following the general procedure, the title compound was isolated as a yellow oil liquid (Yield 91%, 41.1

mg). **^{1}H NMR** (400 MHz, $CDCl_3$): δ 7.45 (d, *J* = 8.4 Hz, 4H), 7.19 (d, *J* = 8.0 Hz, 4H), 2.95-2.86 (m, 2H), 1.24 (d, *J* = 7.2 Hz, 12H). **^{13}C NMR** (101 MHz, $CDCl_3$)：δ 148.4, 134.4, 128.3, 127.4, 33.9, 24.0.

1,2-bis(2-methoxyphenyl)disulfane (2f): CAS Number 59014-89-0.

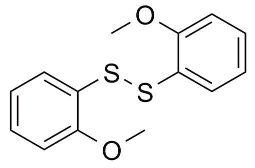

2f

Following the general procedure, the title compound was isolated as a white solid (Yield 95%, 39.8 mg). **^{1}H NMR** (400 MHz, $CDCl_3$)：δ 7.54 (dd, *J* = 8.0, 1.6 Hz, 2H), 7.22-7.17 (m, 2H), 6.92 (td, *J* = 7.6, 1.2 Hz, 2H), 6.86 (dd, *J* = 8.0, 1.2 Hz, 2H), 3.90 (s, 6H). **^{13}C NMR** (101 MHz, $CDCl_3$)：δ 156.6, 127.8, 127.6, 124.6, 121.4, 110.5, 56.0.

1,2-bis(3-methoxyphenyl)disulfane (2g): CAS Number 59014-89-0.

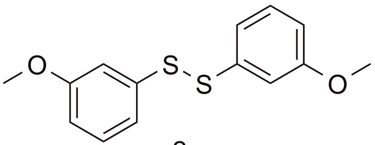

2g

Following the general procedure, the title compound was isolated as a yellow liquid (Yield 97%, 40.3 mg). **^{1}H NMR** (400 MHz, $CDCl_3$)：δ 7.22 (t, *J* = 8.0 Hz, 2H), 7.10–7.07 (m, 4H), 6.78-6.75 (m, 2H), 3.77 (s, 6H). **^{13}C NMR** (101 MHz, $CDCl_3$)：δ 160.1, 138.4, 130.0, 119.6, 113.2, 112.6, 55.4.

1,2-bis(2-chlorophenyl)disulfane (2h): CAS Number 31121-19-4.

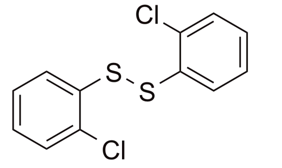

2h

Following the general procedure, the title compound was isolated as a white solid (Yield 88%, 37.8 mg). **^{1}H-NMR** (400 MHz, $CDCl_3$)：δ 7.55 (dt, *J* = 8.0, 1.2 Hz, 2H), 7.37 (dt, *J* = 7.6, 1.2 Hz, 2H), 7.25-7.20 (m, 2H), 7.18-7.14 (m, 2H). **^{13}C-NMR** (101 MHz, $CDCl_3$)：δ 134.4, 131.9, 129.8, 127.9, 127.7, 127.2.

1,2-bis(2-bromophenyl)disulfane (2i): CAS Number 71112-91-9.

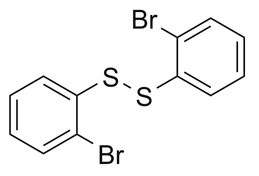

2i

Following the general procedure, the title compound was isolated as a white solid (Yield 87%, 48.9 mg). **^{1}H NMR** (400 MHz, $CDCl_3$)：δ 7.55-7.51 (m, 4H), 7.29–7.25 (m, 2H), 7.08 (td, *J* = 7.6, 1.6 Hz, 2H). **^{13}C NMR** (101 MHz, $CDCl_3$)：δ 136.2, 133.0, 128.3, 128.0, 126.9, 121.1.

1,2-bis(3-chlorophenyl)disulfane (2j): CAS Number 19742-92-8.

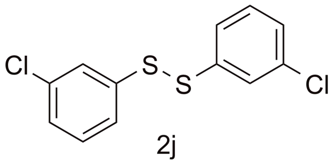
2j

Following the general procedure, the title compound was isolated as a yellow liquid (Yield 89%, 38.3 mg). **^{1}H NMR** (400 MHz, $CDCl_3$): δ 7.47 (s, 2H), 7.35 (dt, *J* = 7.6, 1.6 Hz, 2H), 7.24–7.19 (m, 4H). **^{13}C NMR** (101 MHz, $CDCl_3$): δ 138.5, 135.2, 130.3, 127.7, 127.1, 125.4.

1,2-bis(3-bromophenyl)disulfane (2k): CAS Number 19742-90-6.

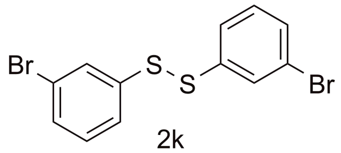
2k

Following the general procedure, the title compound was isolated as a yellow oil liquid (Yield 84%, 47.4 mg). **^{1}H NMR** (400 MHz, $CDCl_3$): δ 7.62 (d, *J* = 1.2 Hz, 2H), 7.41–7.35 (m, 4H), 7.18 (t, *J* = 8.0 Hz, 2H). **^{13}C NMR** (101 MHz, $CDCl_3$): δ 138.7, 130.6, 130.6, 129.9, 125.9, 123.3.

1,2-bis(4-fluorophenyl)disulfane (2l): CAS Number 405-31-2.

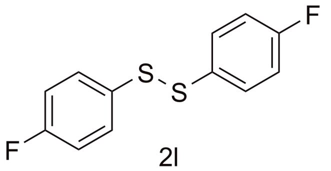
2l

Following the general procedure, the title compound was isolated as a yellow liquid (Yield 99.6%, 38.0 mg). **^{1}H NMR** (400 MHz, $CDCl_3$): δ 7.47–7.43 (m, 4H), 7.04–6.99 (m, 4H). **^{13}C NMR** (101 MHz, $CDCl_3$): δ 162.7 ($J_{C\text{-}F}$ = 249.5 Hz), 132.2 ($J_{C\text{-}F}$ = 3.0 Hz), 131.4 ($J_{C\text{-}F}$ = 8.1 Hz), 116.4 ($J_{C\text{-}F}$ = 22.2 Hz).

1,2-bis(4-chlorophenyl)disulfane (2m): CAS Number 1142-19-4.

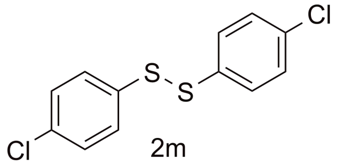
2m

Following the general procedure, the title compound was isolated as a yellowish-white solid (Yield 90%, 38.9 mg). **^{1}H NMR** (400 MHz, $CDCl_3$): δ 7.41–7.38 (m, 4H), 7.29–7.25 (m, 4H). **^{13}C NMR** (101 MHz, $CDCl_3$): δ 135.2, 133.7, 129.4, 129.4.

1,2-bis(3-bromophenyl)disulfane (2n): CAS Number 5335-84-2.

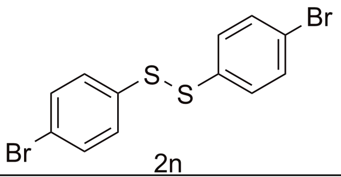
2n

Following the general procedure, the title compound was isolated as a yellowish-white solid (Yield 84%, 47.4 mg). **^{1}H NMR** (400 MHz, $CDCl_3$): δ 7.44–7.41 (m, 4H), 7.34 –7.32 (m, 4H). **^{13}C NMR** (101 MHz, $CDCl_3$):

δ 135.8, 132.3, 129.4, 121.6.

4,4'-disulfanediyldiphenol (2o): CAS Number 15015-57-3.

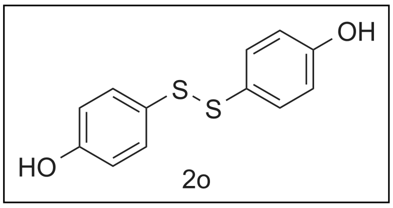

Following the general procedure, the title compound was isolated as a white to light yellow solid (Yield 87%, 32.7 mg). **^{1}H NMR** (400 MHz, $CDCl_3$)：δ 7.27–7.24 (m, 4H), 6.74–6.72 (m, 4H), 4.90 (s, 2H). **^{13}C NMR** (101 MHz, $CDCl_3$)：δ 158.1, 133.3, 126.8, 115.6.

1,2-bis(2,4-dimethylphenyl)disulfane (2p)： CAS Number 13616-83-6.

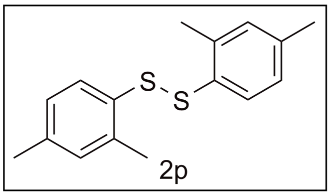

Following the general procedure, the title compound was isolated as a yellow liquid (Yield 89%, 37.8 mg). **^{1}H NMR** (400 MHz, $CDCl_3$)：δ 7.40 (d, *J* = 8.0 Hz, 2H), 7.01 (d, *J* = 2.0 Hz, 2H), 6.95 (dd, *J* = 8.0, 2.0 Hz, 2H), 2.39 (s, 6H), 2.31 (s, 6H). **^{13}C NMR** (101 MHz, $CDCl_3$)：δ 138.3, 137.9, 132.4, 131.3, 130.4, 127.5, 21.1, 20.3.

1,2-bis(2,6-dimethylphenyl)disulfane (2q)： CAS Number 2905-17-1.

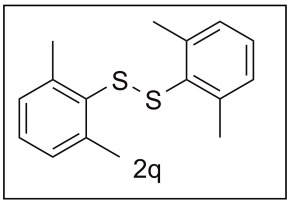

Following the general procedure, the title compound was isolated as a white solid (Yield 86%, 35.5 mg). **^{1}H-NMR** (400 MHz, $CDCl_3$): δ 7.12 (dd, *J* = 8.0, 6.8 Hz, 2H), 7.02 (d, *J* = 7.6 Hz, 4H), 2.24 (s, 12H). **^{13}C-NMR** (101 MHz, $CDCl_3$)：δ 143.5, 134.8, 129.4, 128.2, 21.6.

1,2-bis(3,4-dimethoxyphenyl)disulfane (2r)： CAS Number 66086-38-2.

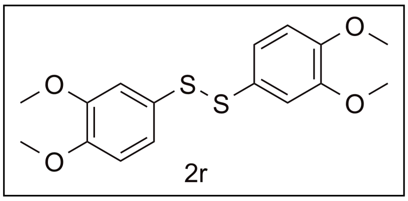

Following the general procedure, the title compound was isolated as a yellow solid (Yield 93%, 55.4 mg). **^{1}H NMR** (400 MHz, $CDCl_3$)：δ 7.03 (dt, *J* = 8.0, 2.0 Hz, 2H), 7.00–6.99 (m, 2H), 6.77 (d, *J* = 8.4 Hz, 2H), 3.86 (s, 6H), 3.82 (s, 6H). **^{13}C NMR** (101 MHz, $CDCl_3$)：δ 149.5, 149.1, 128.7, 124.0, 114.1, 111.3, 56.0, 55.9.

1,2-bis(2,5-dimethoxyphenyl)disulfane (2s)： CAS Number 29945-69-5.

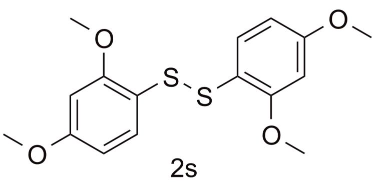

2s

Following the general procedure, the title compound was isolated as a yellow solid (Yield 99.9%, 50.7 mg). **^{1}H NMR** (400 MHz, $CDCl_3$)：δ 7.15 (d, *J* = 2.8 Hz, 2H), 6.77 (d, *J* = 8.8 Hz, 2H), 6.68 (dd, *J* = 8.8, 2.8 Hz, 2H), 3.85 (s, 6H), 3.70 (s, 6H). **^{13}C NMR** (101 MHz, $CDCl_3$)：δ 154.3, 150.9, 125.7, 113.5, 112.4, 111.7, 56.6, 55.8.

1,2-bis(2,4-dichlorophenyl)disulfane (2t)： CAS Number 15433-50-8.

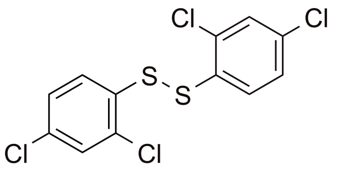

2t

Following the general procedure, the title compound was isolated as a white solid (Yield 90%, 48.0 mg). **^{1}H NMR** (400 MHz, $CDCl_3$)：δ 7.45 (d, *J* = 8.4 Hz, 2H), 7.39 (d, *J* = 2.0 Hz, 2H), 7.21 (dd, *J* = 8.4, 2.0 Hz, 2H). **^{13}C NMR** (101 MHz, $CDCl_3$)：δ 133.6, 132.9, 132.8, 129.7, 128.6, 128.1.

1,2-dibenzyldisulfane (2u)： CAS Number 150-60-7.

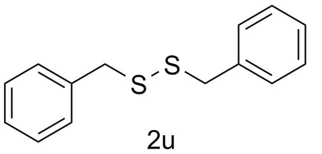

2u

Following the general procedure, the title compound was isolated as a colorless oil liquid (Yield 88%, 32.5 mg). **^{1}H-NMR** (400 MHz, $CDCl_3$)： δ 7.37–7.24 (m, 10H), 3.61 (s, 4H). **^{13}C-NMR** (101 MHz, $CDCl_3$)： δ 137.4, 129.5, 128.6, 127.6, 43.3.

1,2-di(thiophen-2-yl)disulfane (2v): CAS Number 6911-51-9.

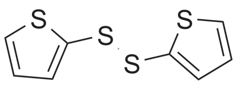

2v

Following the general procedure, the title compound was isolated as a yellow solid (Yield 72%, 24.9 mg). **^{1}H-NMR** (400 MHz, $CDCl_3$): δ 7.50 (dd, *J* = 5.6, 1.6 Hz, 2H), 7.16 (dd, *J* = 3.6, 1.2 Hz, 2H), 7.02 (dd, *J* = 5.2, 3.6 Hz, 2H). **^{13}C-NMR** (101 MHz, $CDCl_3$)：δ 135.8, 135.8, 132.4, 127.9.

1,2-di(pyrimidin-2-yl)disulfane (2w): CAS Number 15718-46-4.

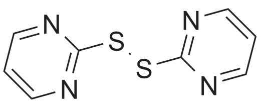

2w

Following the general procedure, the title compound was isolated as a white solid (Yield 41%, 13.5 mg). **^{1}H-NMR** (400 MHz, $CDCl_3$): δ 8.57 (d, *J* = 4.8 Hz, 4H), 7.08 (t, *J* =

4.8 Hz, 2H). **^{13}C-NMR** (101 MHz, $CDCl_3$): δ 169.8, 158.0, 118.3.

1,2-dicyclohexyldisulfane (2x): CAS Number 2550-40-5.

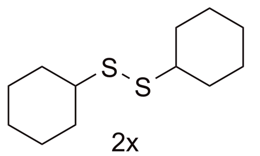

2x

Following the general procedure the title compound was isolated as a yellow liquid (Yield 68%, 23.3 mg). **^{1}H-NMR** (400 MHz, $CDCl_3$): δ 2.71-2.64 (m, 2H), 2.06-2.01 (m, 4H), 1.80-1.75 (m, 4H), 1.63 – 1.59 (m, 2H), 1.33-1.25 (m, 10H). **^{13}C-NMR** (101 MHz, $CDCl_3$): δ 50.1, 33.0, 26.2, 25.8.

1,2-dipropyldisulfane (2y): CAS Number 629-19-6.

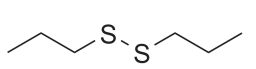

2y

Following the general procedure the title compound was isolated as a colorless to pale yellow liquid (Yield 99%, 22.3 mg). **^{1}H-NMR** (400 MHz, $CDCl_3$): δ 2.66 (t, J = 7.2 Hz, 4H), 1.75-1.66 (m, 4H), 0.99 (t, J = 7.6 Hz, 6H). **^{13}C-NMR** (101 MHz, $CDCl_3$): δ 41.3, 22.6, 13.3.

1,2-dioctyldisulfane (2z): CAS Number 822-27-5.

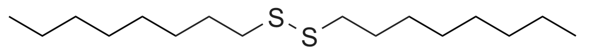

2z

Following the general procedure the title compound was isolated as a yellow-brown liquid (Yield 82%, 35.6 mg). **^{1}H-NMR** (400 MHz, $CDCl_3$): δ 2.68 (t, J = 7.2 Hz, 4H), 1.70-1.63 (m, 4H), 1.37 (t, J = 8.0 Hz, 4H), 1.30-1.27 (m, 16H), 0.88 (t, J = 6.4 Hz, 6H). **^{13}C-NMR** (101 MHz, $CDCl_3$): δ 39.3, 32.0, 29.4, 29.3, 29.3, 28.7, 22.8, 14.2.

1,2-bis(4-chlorobenzyl)disulfane (2aa): CAS Number 23566-17-8.

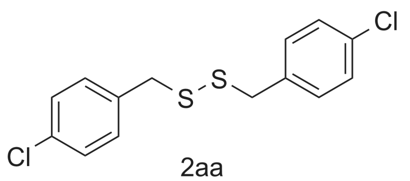

2aa

Following the general procedure the title compound was isolated as a colorless oily liquid (Yield 32%, 30.0 mg). **^{1}H-NMR** (400 MHz, $CDCl_3$): δ 7.31–7.29 (m, 4H), 7.17–7.15 (m, 4H), 3.58 (s, 4H). **^{13}C-NMR** (101 MHz, $CDCl_3$): δ 136.0, 133.5, 130.8, 128.8, 42.6.

1,2-bis(4-(trifluoromethyl)phenyl)disulfane (2ab): CAS Number 18715-45-2.

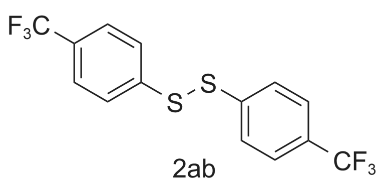

2ab

Following the general procedure the title compound was isolated as a white solid (Yield 21%, 21.9 mg). **^{1}H-NMR** (400 MHz, $CDCl_3$): δ 7.60-7.56 (m, 8H). **^{13}C-NMR** (101 MHz, $CDCl_3$): δ 140.9, 129.6 (q, J_{C-F} = 32.3 Hz), 126.7, 126.3 (q, J_{C-F} = 4.04 Hz), 124.0 (q, J_{C-F} = 272.7 Hz).

1-(4-methoxyphenyl)-2-(o-tolyl)disulfane (3a): CAS Number 2445788-86-1.

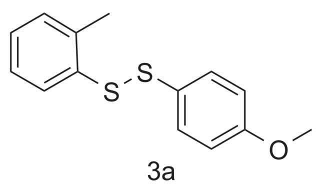

3a

Following the general procedure the title compound was isolated as a yellow oily liquid (Yield 33%, 26.3 mg). **^{1}H-NMR** (400 MHz, $CDCl_3$): δ 7.63–7.59 (m, 1H), 7.43–7.39 (m, 2H), 7.17 (d, J = 3.2 Hz, 3H), 6.84–6.81 (m, 2H), 3.79 (s, 3H), 2.39 (s, 3H). **^{13}C-NMR** (101 MHz, $CDCl_3$): δ 160.0, 137.8, 136.1, 132.3, 130.5, 129.4, 128.0, 127.6, 126.7, 114.8, 55.5, 20.2.

1-(4-chlorobenzyl)-2-(4-methoxyphenyl)disulfane (3aa): CAS Number 2492436-80-1.

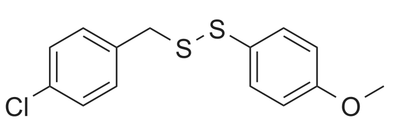

3aa

Following the general procedure the title compound was isolated as a yellow oily liquid (Yield 33%, 29.6 mg). **^{1}H-NMR** (400 MHz, $CDCl_3$): δ 7.35 (d, J = 8.4 Hz, 2H), 7.24–7.17 (m, 4H), 6.83–6.81 (m, 2H), 3.89 (s, 2H), 3.81 (s, 3H). **^{13}C-NMR** (101 MHz, $CDCl_3$): δ 159.8, 135.5, 133.4, 132.2, 130.9, 128.7, 127.8, 114.7, 55.5, 42.5.

1-(4-methoxyphenyl)-2-(4-(trifluoromethyl)phenyl)disulfane (3ab): CAS Number 1670249-61-2.

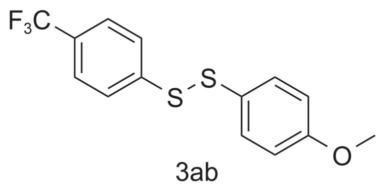

3ab

Following the general procedure the title compound was isolated as a yellow oily liquid (Yield 46%, 43.6 mg). **^{1}H-NMR** (400 MHz, $CDCl_3$): δ 7.63 (d, J = 7.6 Hz, 2H), 7.56 (d, J = 8.4 Hz, 2H), 7.45–7.41 (m, 2H), 6.86–6.83 (m, 2H), 3.79 (s, 3H). **^{13}C-NMR** (101 MHz, $CDCl_3$): δ 160.2, 142.5 (d, J_{C-F} = 2.02 Hz), 132.0, 129.0 (q, J_{C-F} = 33.3 Hz), 127.2, 127.1, 126.0 (q, J_{C-F} = 4.04 Hz), 124.2 (q, J_{C-F} = 272.7 Hz), 115.0, 55.5.

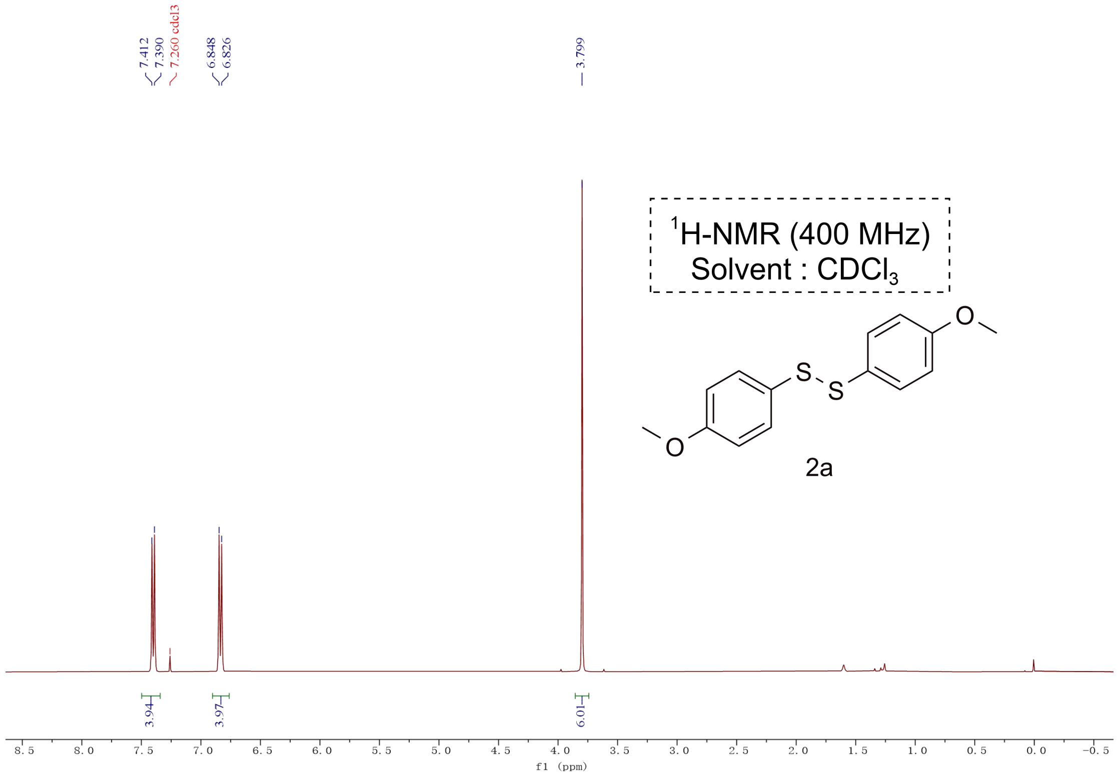
7.412
7.390
7.260 cdcl3
6.848
6.826
3.799
1H-NMR (400 MHz)
Solvent : CDCl3
O
S
S
O
2a
3.94
3.97
6.01
8.5 8.0 7.5 7.0 6.5 6.0 5.5 5.0 4.5 4.0 3.5 3.0 2.5 2.0 1.5 1.0 0.5 0.0 -0.5
f1 (ppm)

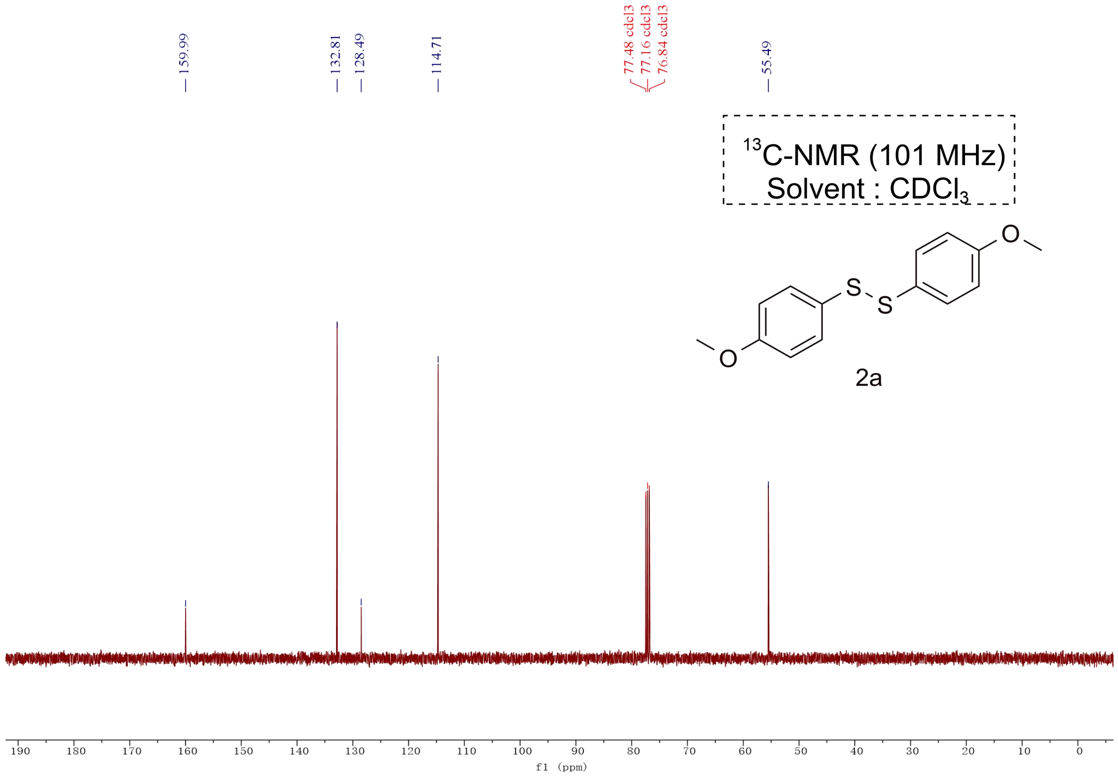
159.99
132.81
128.49
114.71
77.48 cdcl3
77.16 cdcl3
76.84 cdcl3
55.49
13C-NMR (101 MHz)
Solvent : CDCl3
O
S
S
O
2a
190 180 170 160 150 140 130 120 110 100 90 80 70 60 50 40 30 20 10 0
f1 (ppm)

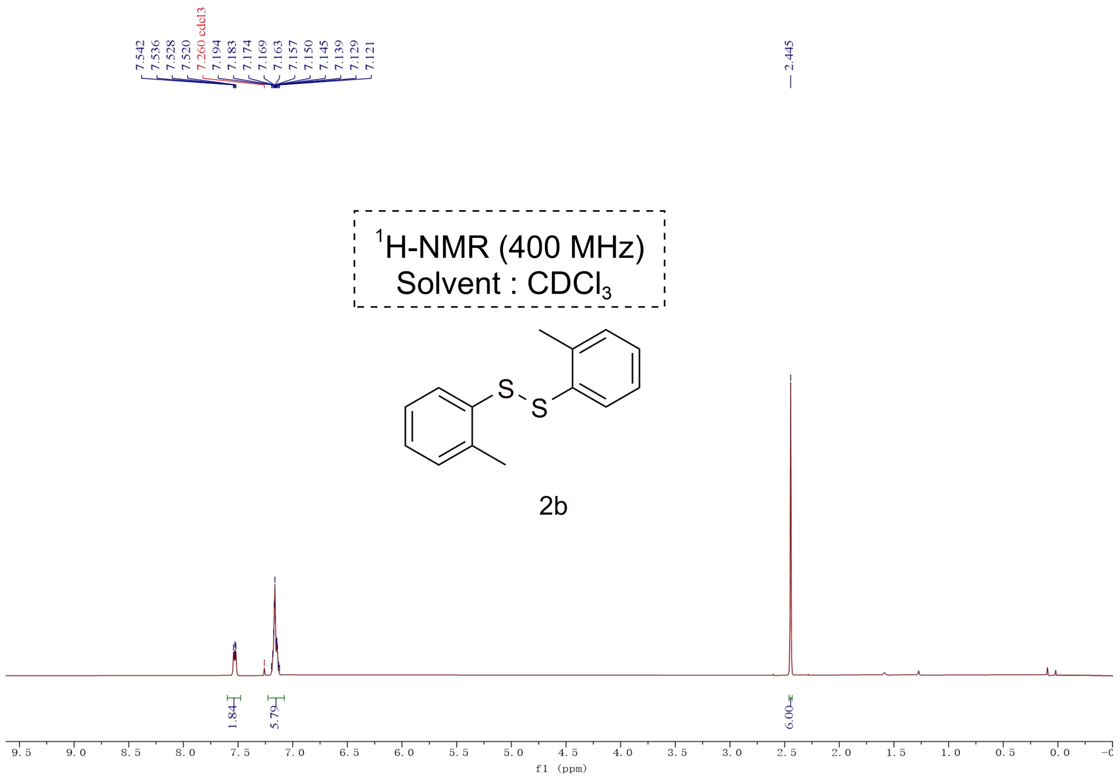
7.542
7.536
7.528
7.520
7.260 cdcl3
7.194
7.183
7.174
7.169
7.163
7.157
7.150
7.145
7.139
7.129
7.121
2.445
^{1}H-NMR (400 MHz)
Solvent : $CDCl_3$
S
S
2b
1.84
5.79
6.00
f1 (ppm)

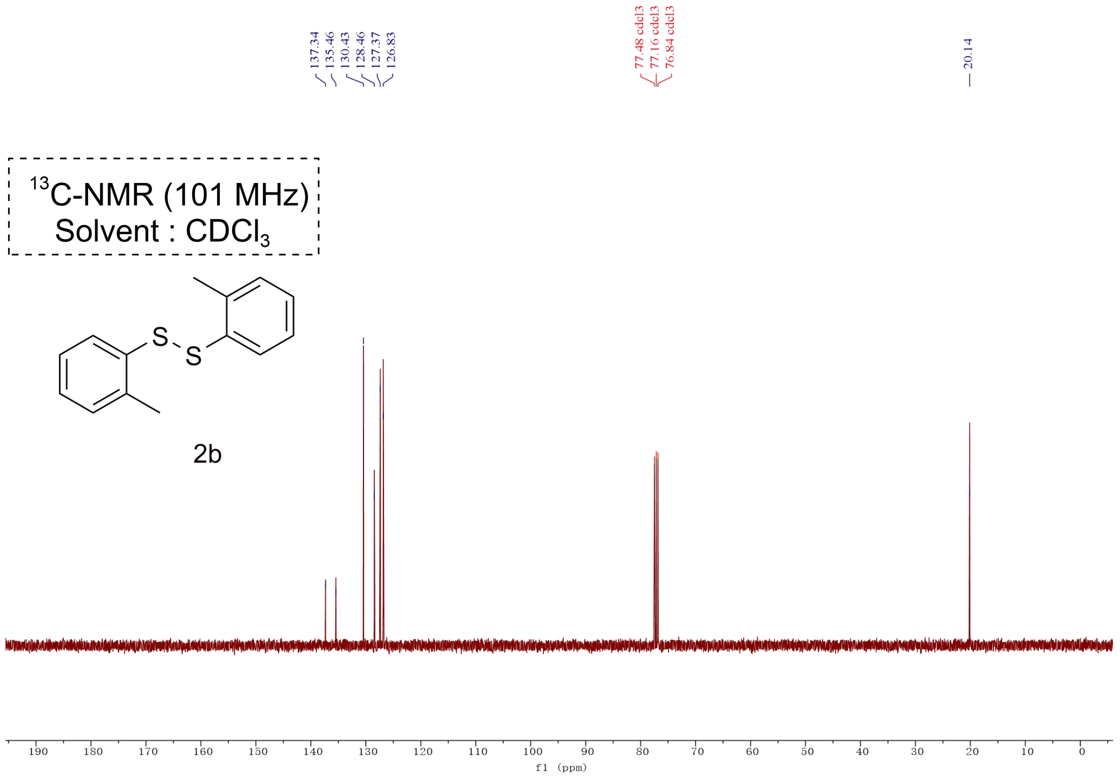
137.34
135.46
130.43
128.46
127.37
126.83
77.48 cdcl3
77.16 cdcl3
76.84 cdcl3
20.14
^{13}C-NMR (101 MHz)
Solvent : $CDCl_3$
S
S
2b
f1 (ppm)

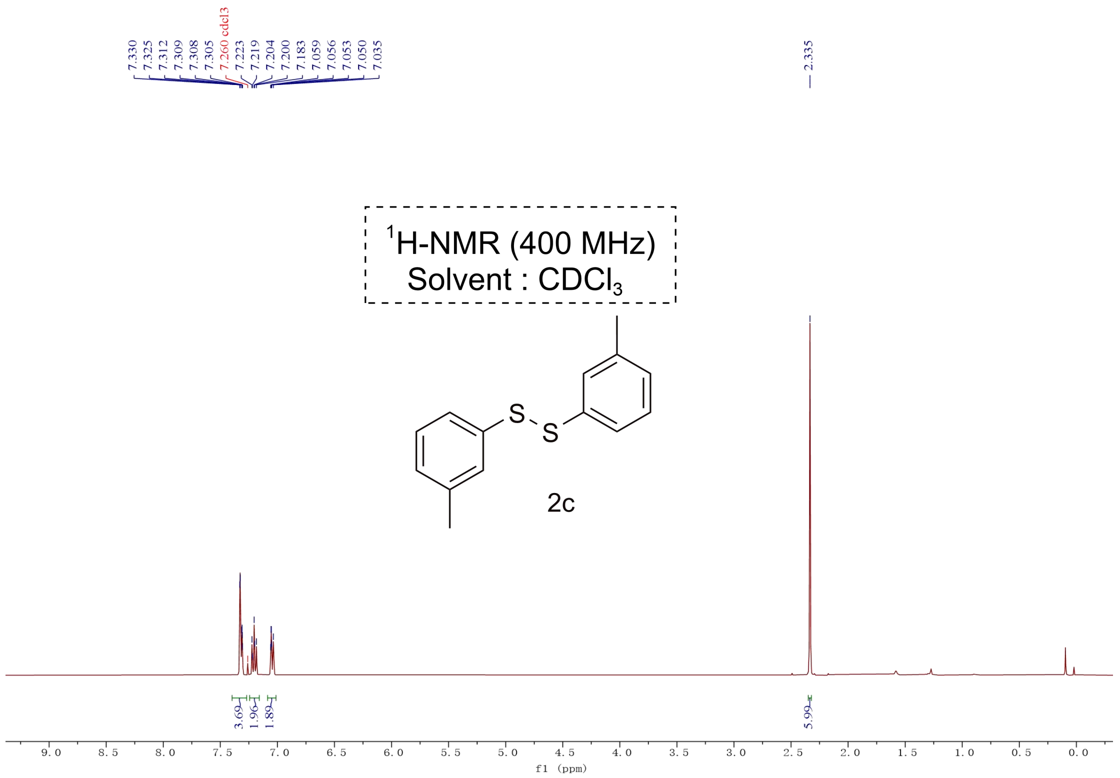

7.330
7.325
7.312
7.309
7.308
7.305
7.260 cdcl3
7.223
7.219
7.204
7.200
7.183
7.059
7.056
7.053
7.050
7.035
2.335
1H-NMR (400 MHz)
Solvent : CDCl3
S
S
2c
3.69
1.96
1.89
5.99
9.0
8.5
8.0
7.5
7.0
6.5
6.0
5.5
5.0
4.5
4.0
3.5
3.0
2.5
2.0
1.5
1.0
0.5
0.0
f1 (ppm)

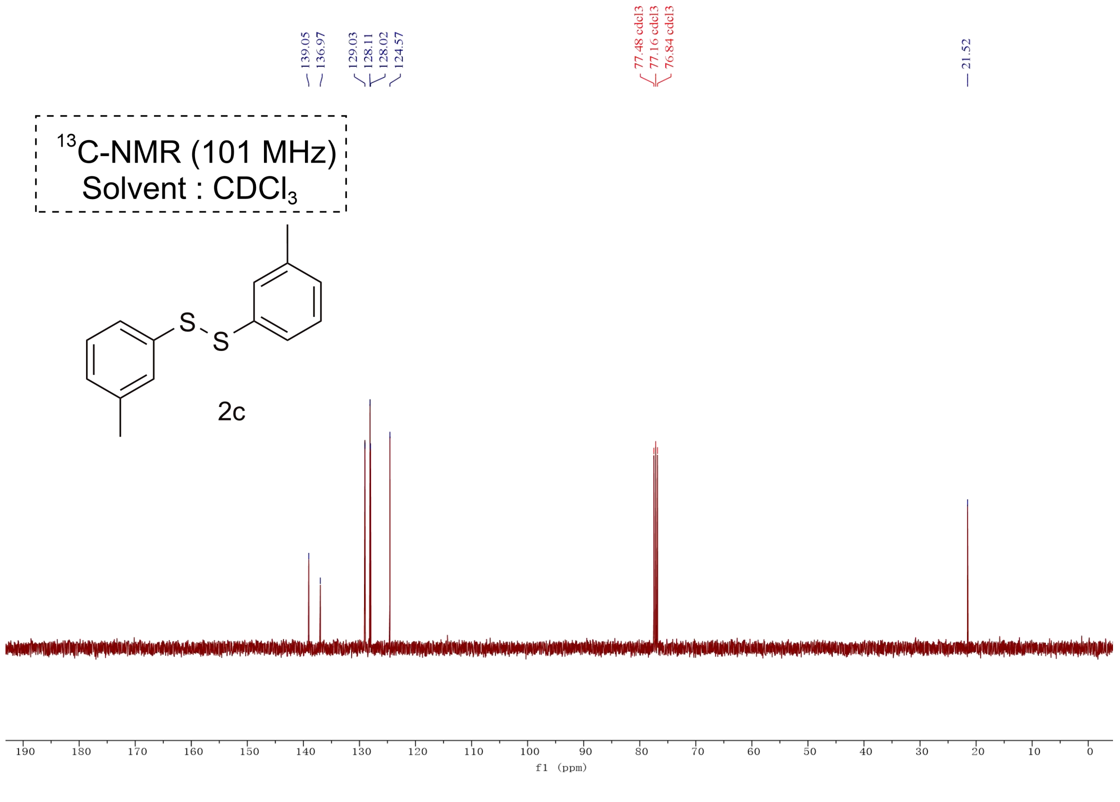

139.05
136.97
129.03
128.11
128.02
124.57
77.48 cdcl3
77.16 cdcl3
76.84 cdcl3
21.52
13C-NMR (101 MHz)
Solvent : CDCl3
S
S
2c
190
180
170
160
150
140
130
120
110
100
90
80
70
60
50
40
30
20
10
0
f1 (ppm)

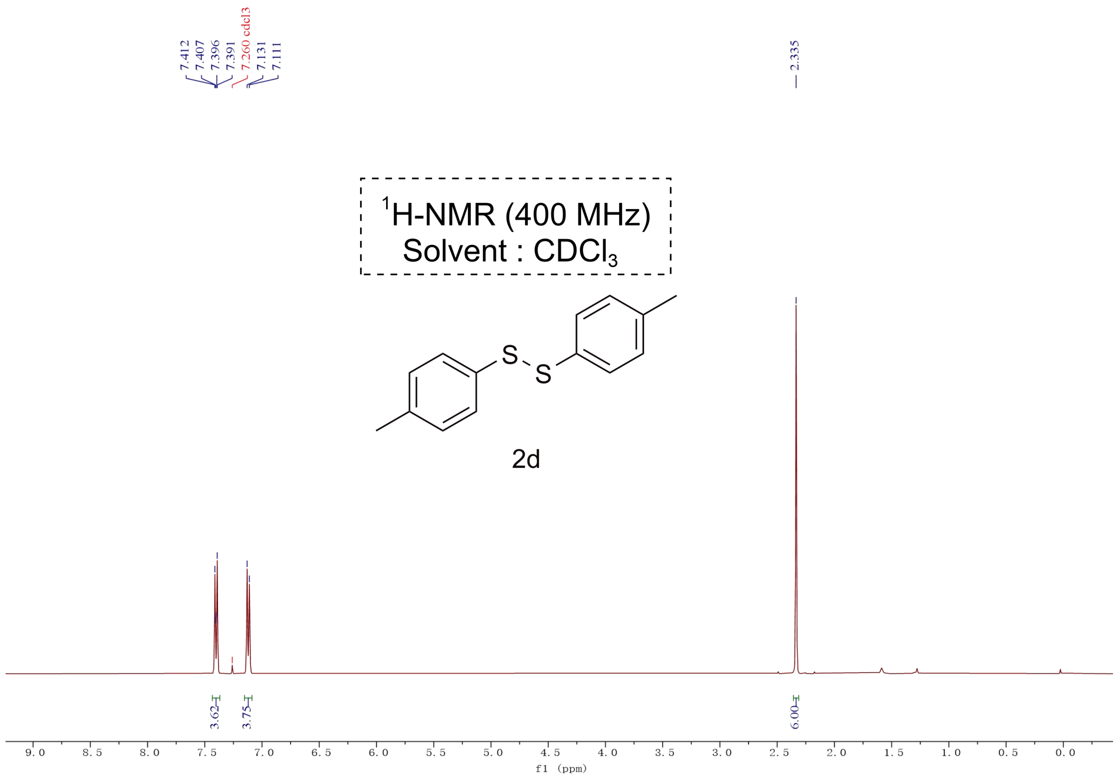
7.412
7.407
7.396
7.391
7.260 cdcl3
7.131
7.111
2.335
1H-NMR (400 MHz)
Solvent : CDCl3
S
S
2d
3.62
3.75
6.00
9.0 8.5 8.0 7.5 7.0 6.5 6.0 5.5 5.0 4.5 4.0 3.5 3.0 2.5 2.0 1.5 1.0 0.5 0.0
f1 (ppm)

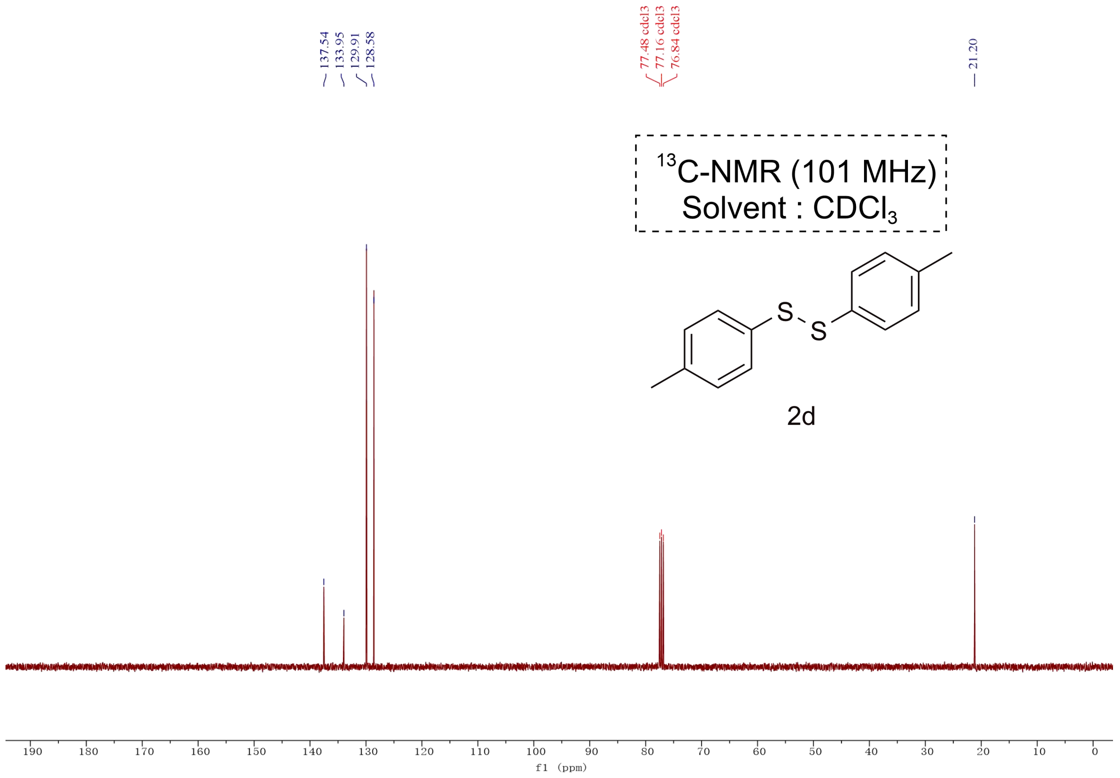
137.54
133.95
129.91
128.58
77.48 cdcl3
77.16 cdcl3
76.84 cdcl3
21.20
13C-NMR (101 MHz)
Solvent : CDCl3
S
S
2d
190 180 170 160 150 140 130 120 110 100 90 80 70 60 50 40 30 20 10 0
f1 (ppm)

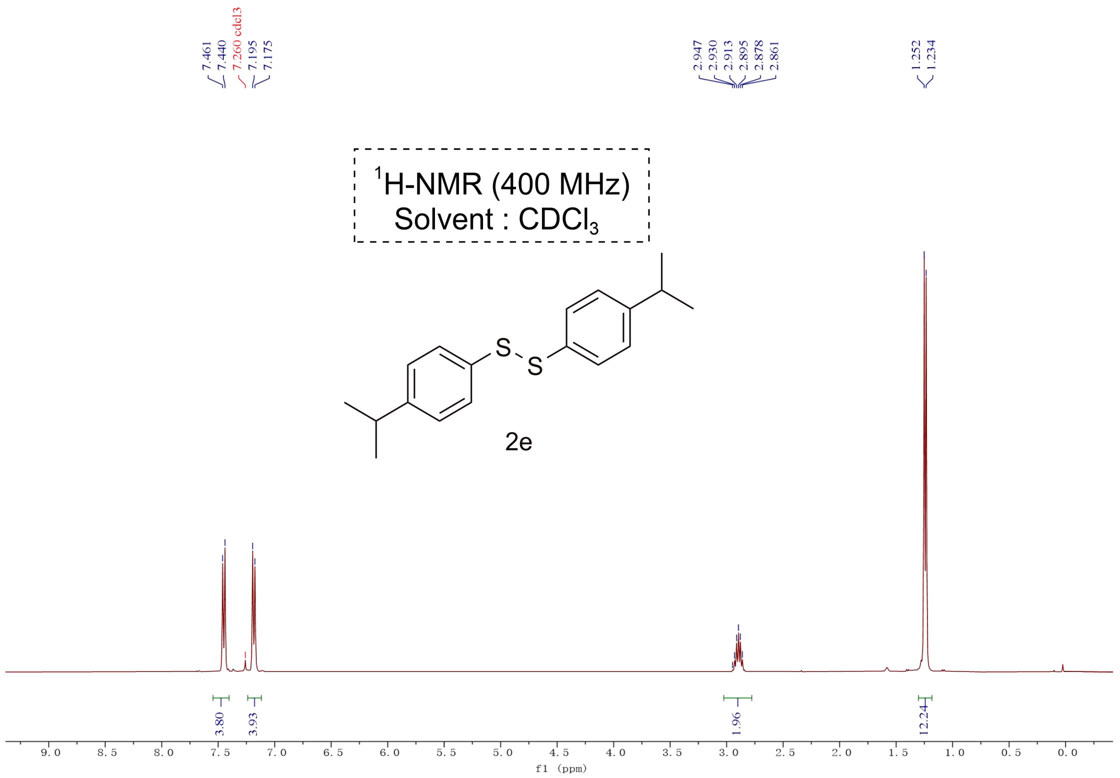
7.461
7.440
7.260 cdcl3
7.195
7.175
2.947
2.930
2.913
2.895
2.878
2.861
1.252
1.234
1H-NMR (400 MHz)
Solvent : CDCl3
S
S
2e
3.80
3.93
1.96
12.24
9.0 8.5 8.0 7.5 7.0 6.5 6.0 5.5 5.0 4.5 4.0 3.5 3.0 2.5 2.0 1.5 1.0 0.5 0.0
f1 (ppm)

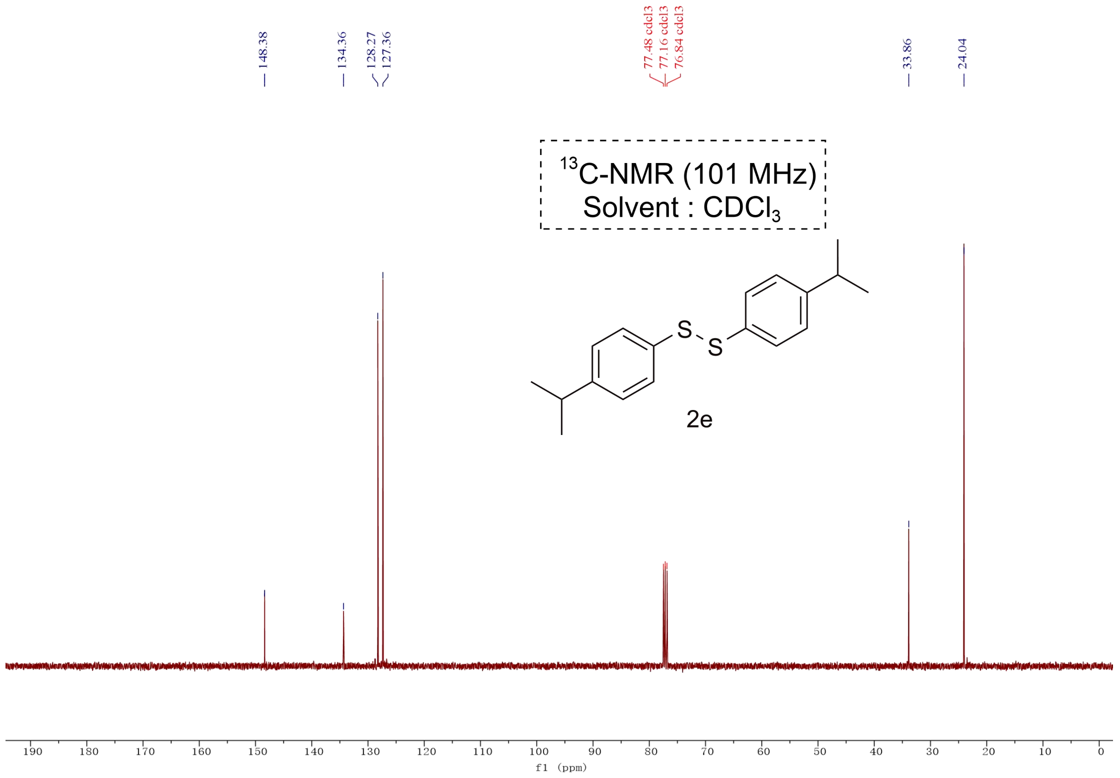
148.38
134.36
128.27
127.36
77.48 cdcl3
77.16 cdcl3
76.84 cdcl3
33.86
24.04
13C-NMR (101 MHz)
Solvent : CDCl3
S
S
2e
190 180 170 160 150 140 130 120 110 100 90 80 70 60 50 40 30 20 10 0
f1 (ppm)

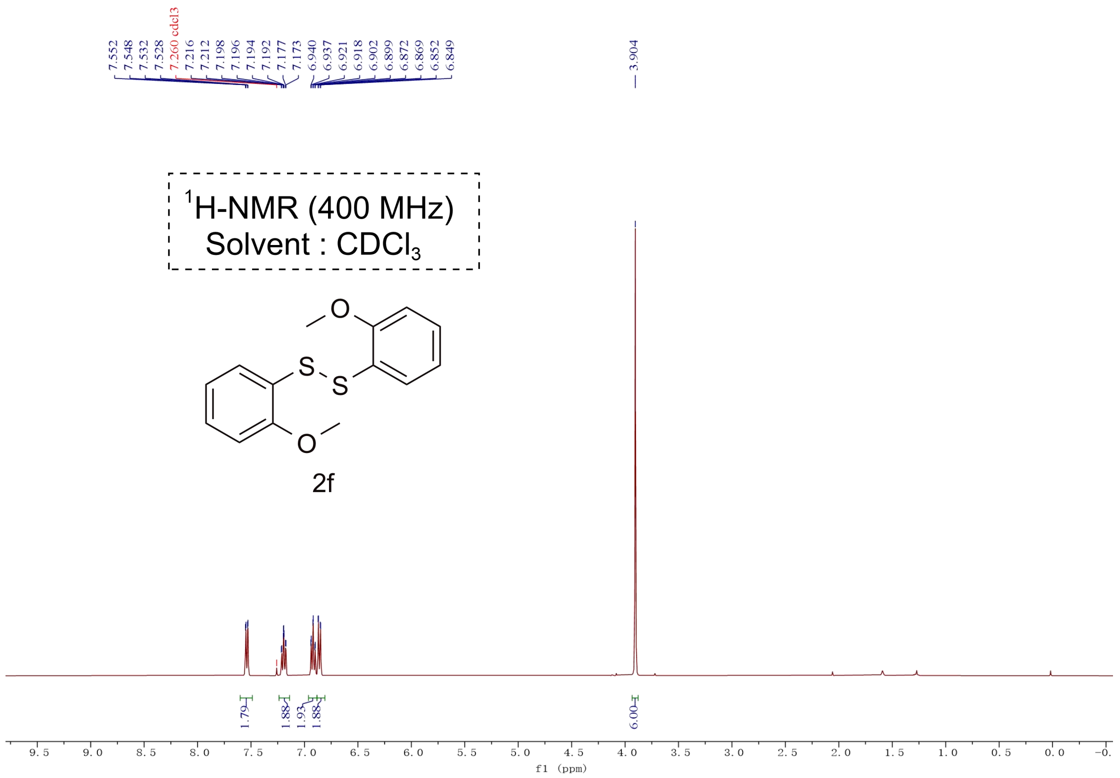
7.552
7.548
7.532
7.528
7.260 cdcl3
7.216
7.212
7.198
7.196
7.194
7.192
7.177
7.173
6.940
6.937
6.921
6.918
6.902
6.899
6.872
6.869
6.852
6.849
3.904
¹H-NMR (400 MHz)
Solvent : CDCl3
O
S
S
O
2f
1.79
1.88
1.93
1.88
6.00
f1 (ppm)

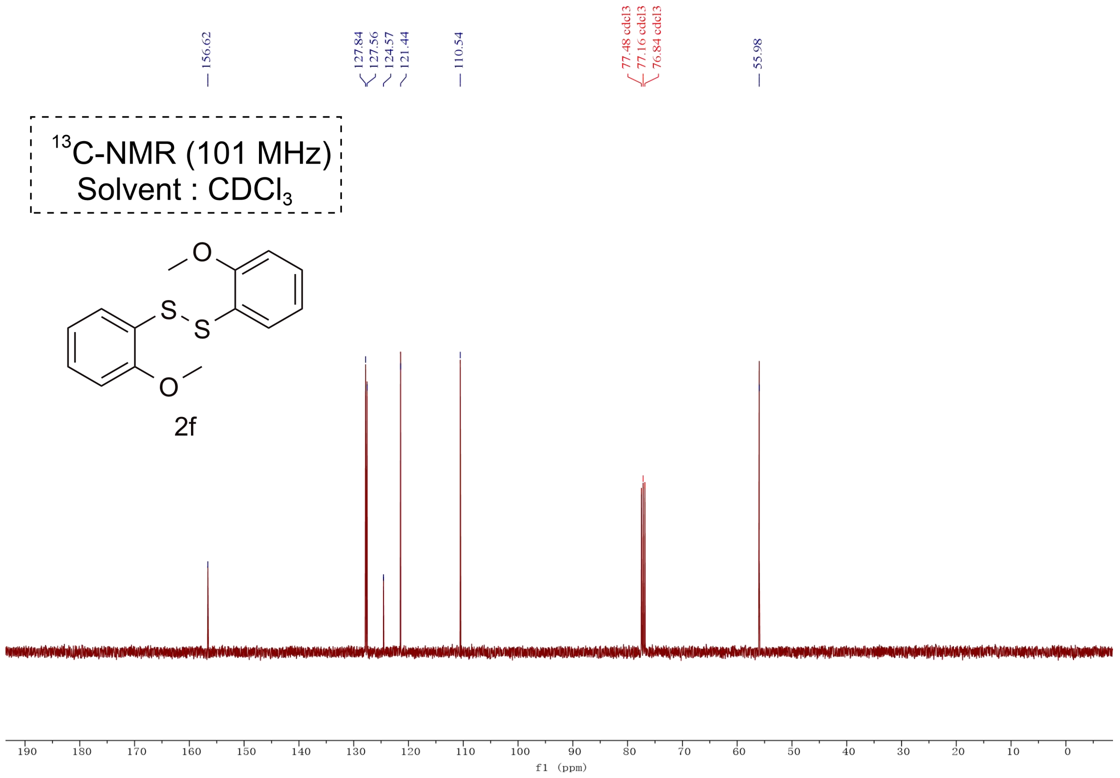
156.62
127.84
127.56
124.57
121.44
110.54
77.48 cdcl3
77.16 cdcl3
76.84 cdcl3
55.98
¹³C-NMR (101 MHz)
Solvent : CDCl3
O
S
S
O
2f
f1 (ppm)

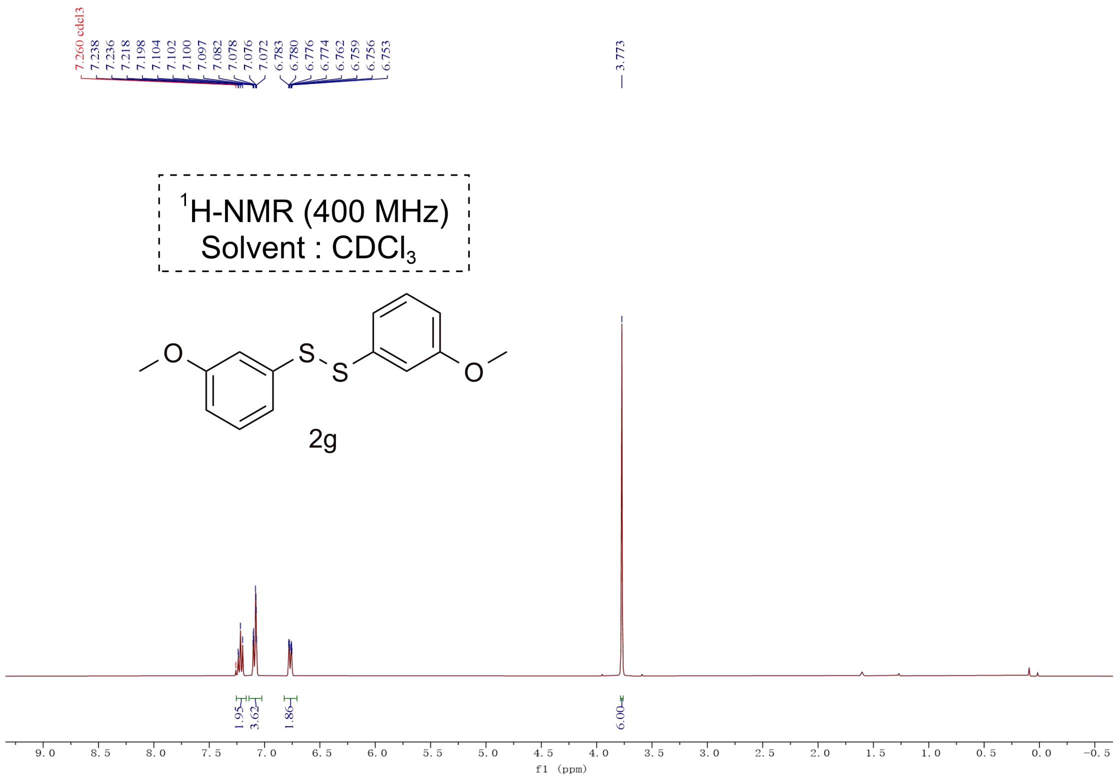
7.260 cdcl3
7.238
7.236
7.218
7.198
7.104
7.102
7.100
7.097
7.082
7.078
7.076
7.072
6.783
6.780
6.776
6.774
6.762
6.759
6.756
6.753
3.773
^{1}H-NMR (400 MHz)
Solvent : $CDCl_3$
2g
1.95
3.62
1.86
6.00
f1 (ppm)

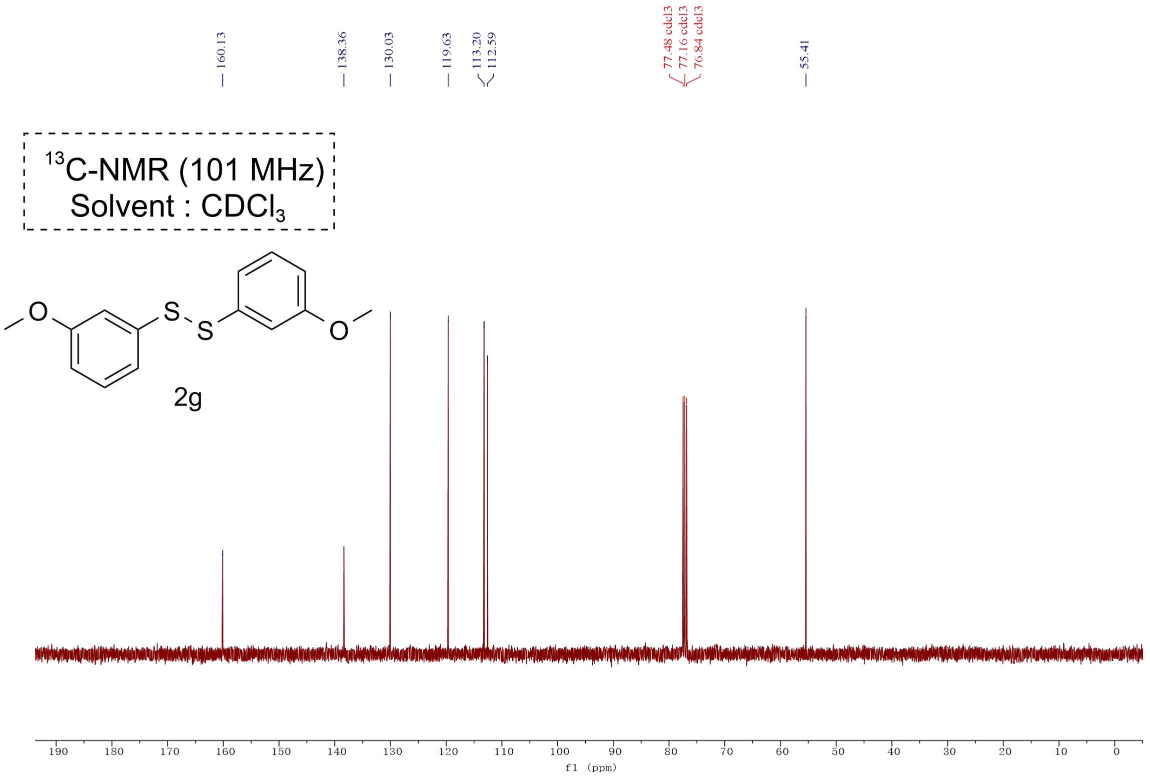
160.13
138.36
130.03
119.63
113.20
112.59
77.48 cdcl3
77.16 cdcl3
76.84 cdcl3
55.41
^{13}C-NMR (101 MHz)
Solvent : $CDCl_3$
2g
f1 (ppm)

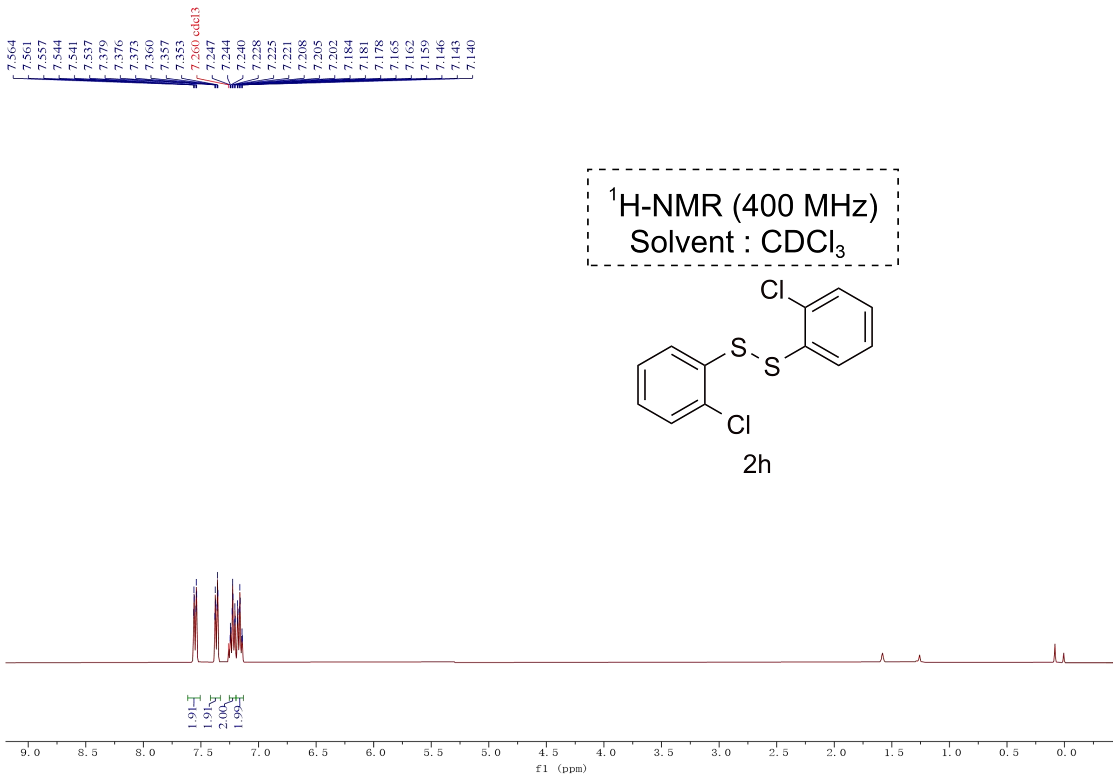
7.564 7.561 7.557 7.544 7.541 7.537 7.379 7.376 7.373 7.360 7.357 7.353 7.260 cdcl3 7.247 7.244 7.240 7.228 7.225 7.221 7.208 7.205 7.202 7.184 7.181 7.178 7.165 7.162 7.159 7.146 7.143 7.140
^{1}H-NMR (400 MHz)
Solvent : $CDCl_3$
Cl
S
S
Cl
2h
1.91 1.91 2.00 1.99
9.0 8.5 8.0 7.5 7.0 6.5 6.0 5.5 5.0 4.5 4.0 3.5 3.0 2.5 2.0 1.5 1.0 0.5 0.0
f1 (ppm)

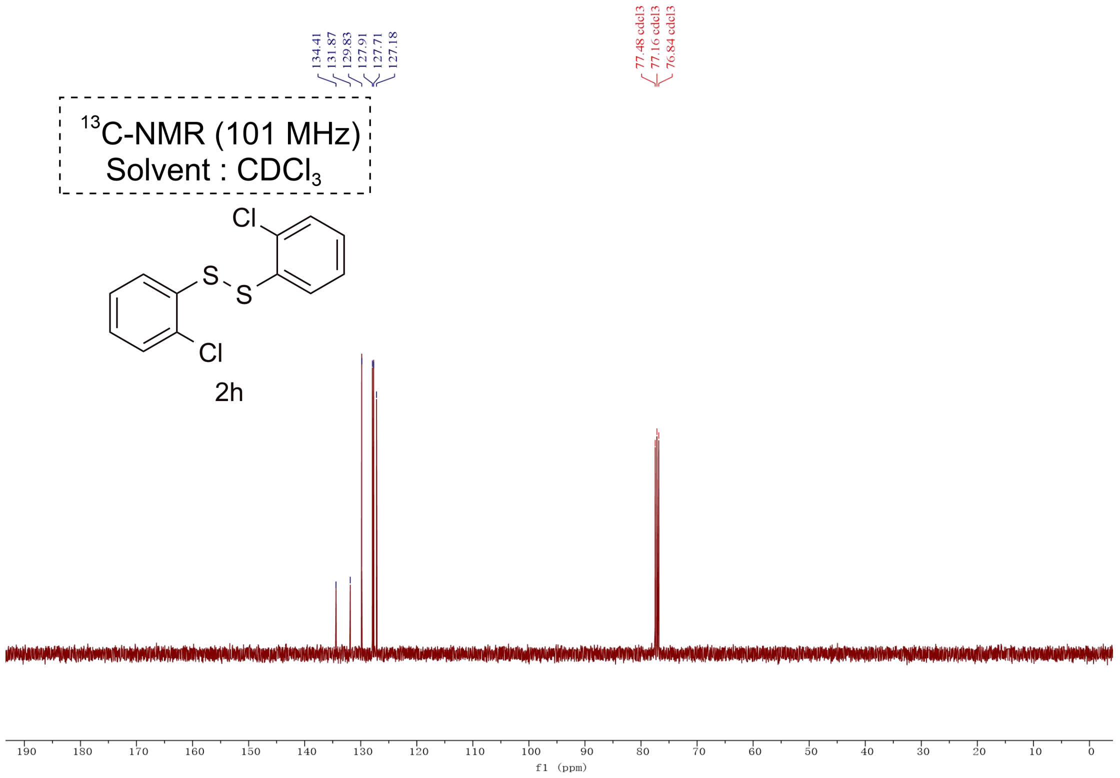
134.41 131.87 129.83 127.91 127.71 127.18
77.48 cdcl3 77.16 cdcl3 76.84 cdcl3
^{13}C-NMR (101 MHz)
Solvent : $CDCl_3$
Cl
S
S
Cl
2h
190 180 170 160 150 140 130 120 110 100 90 80 70 60 50 40 30 20 10 0
f1 (ppm)

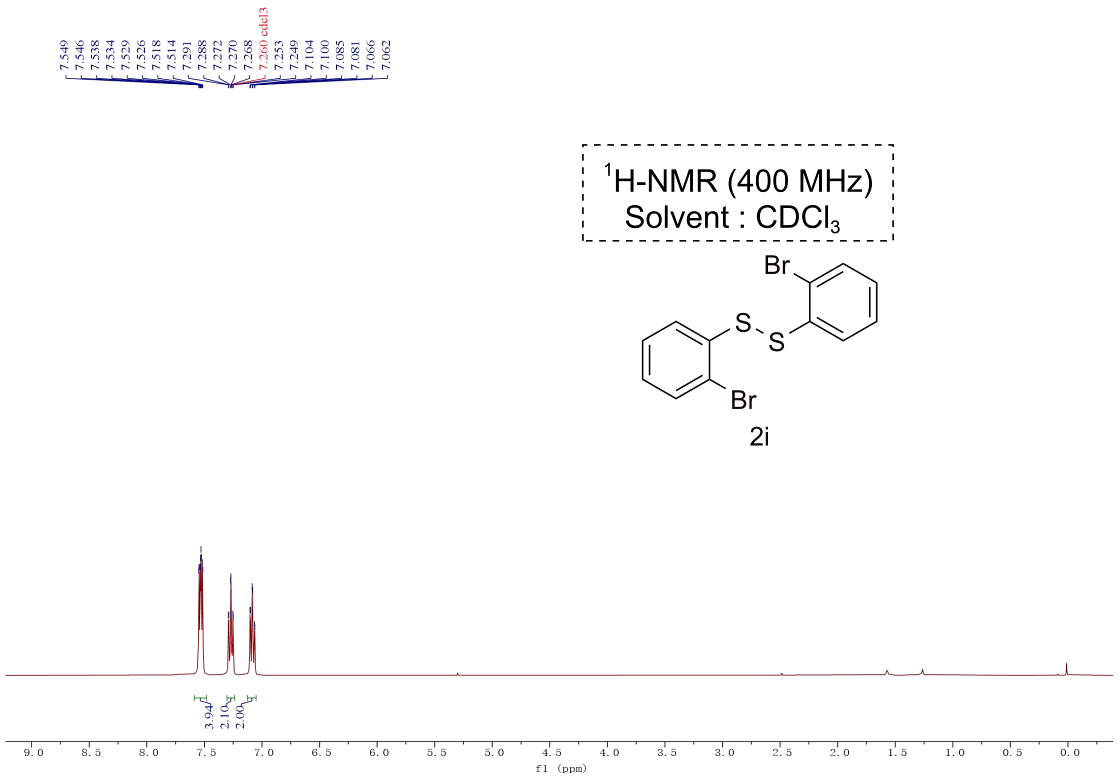
7.549 7.546 7.538 7.534 7.529 7.526 7.518 7.514 7.291 7.288 7.272 7.270 7.268 7.260 cdcl3 7.253 7.249 7.104 7.100 7.085 7.081 7.066 7.062
^{1}H-NMR (400 MHz)
Solvent : $CDCl_3$
Br
S
S
Br
2i
3.94
2.10
2.00
9.0 8.5 8.0 7.5 7.0 6.5 6.0 5.5 5.0 4.5 4.0 3.5 3.0 2.5 2.0 1.5 1.0 0.5 0.0
f1 (ppm)

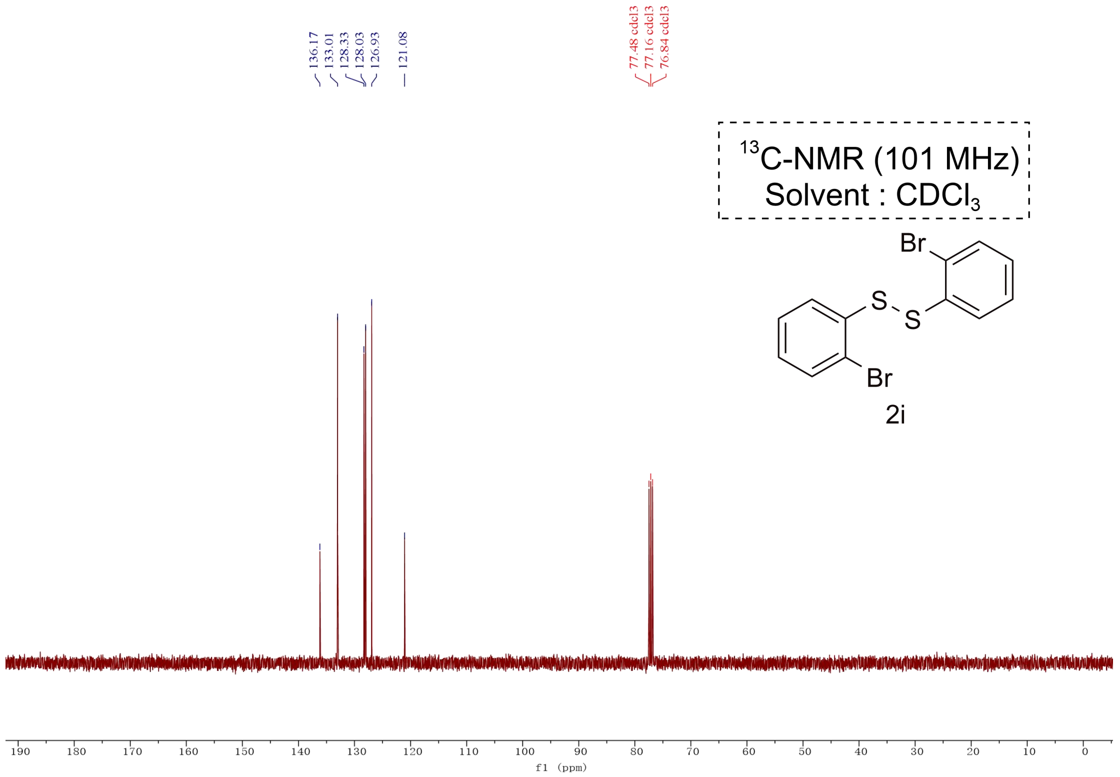
136.17 133.01 128.33 128.03 126.93 121.08
77.48 cdcl3 77.16 cdcl3 76.84 cdcl3
^{13}C-NMR (101 MHz)
Solvent : $CDCl_3$
Br
S
S
Br
2i
190 180 170 160 150 140 130 120 110 100 90 80 70 60 50 40 30 20 10 0
f1 (ppm)

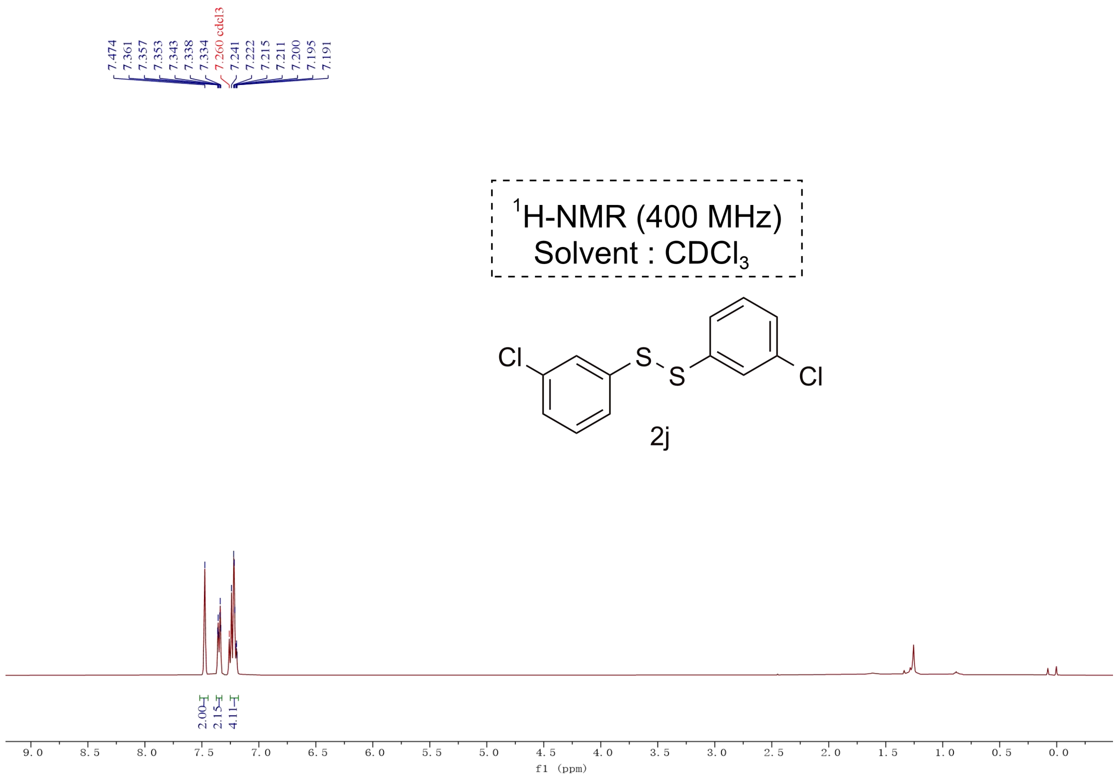
7.474
7.361
7.357
7.353
7.343
7.338
7.334
7.260 cdcl3
7.241
7.222
7.215
7.211
7.200
7.195
7.191
^{1}H-NMR (400 MHz)
Solvent : CDCl$_3$
Cl
S
S
Cl
2j
2.00
2.15
4.11
f1 (ppm)

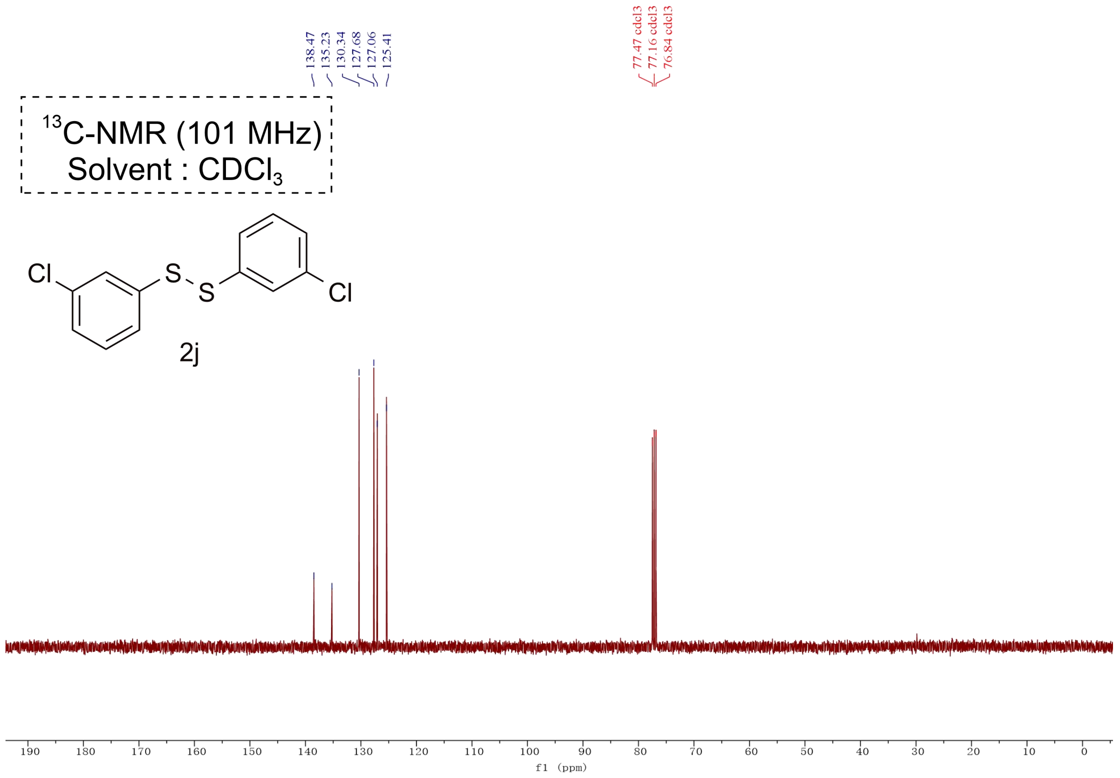
138.47
135.23
130.34
127.68
127.06
125.41
77.47 cdcl3
77.16 cdcl3
76.84 cdcl3
^{13}C-NMR (101 MHz)
Solvent : CDCl$_3$
Cl
S
S
Cl
2j
f1 (ppm)

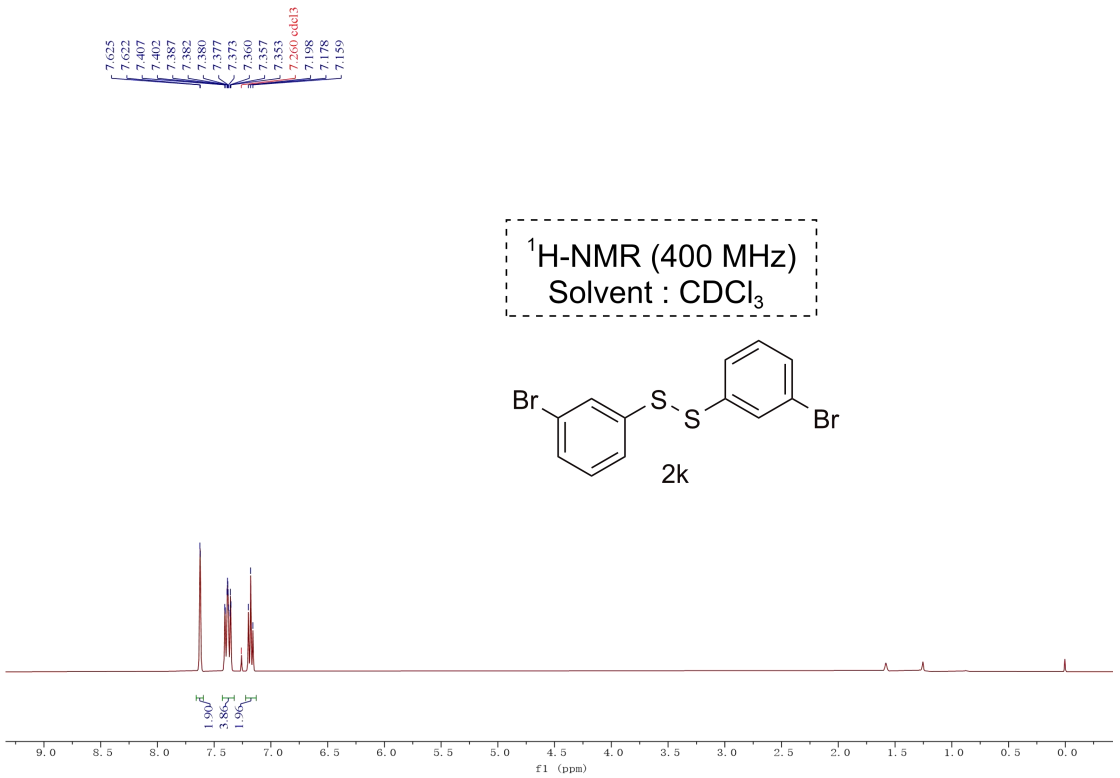
7.625
7.622
7.407
7.402
7.387
7.382
7.380
7.377
7.373
7.360
7.357
7.353
7.260 cdcl3
7.198
7.178
7.159
^{1}H-NMR (400 MHz)
Solvent : $CDCl_3$
Br
S
S
Br
2k
1.90
3.86
1.96
f1 (ppm)

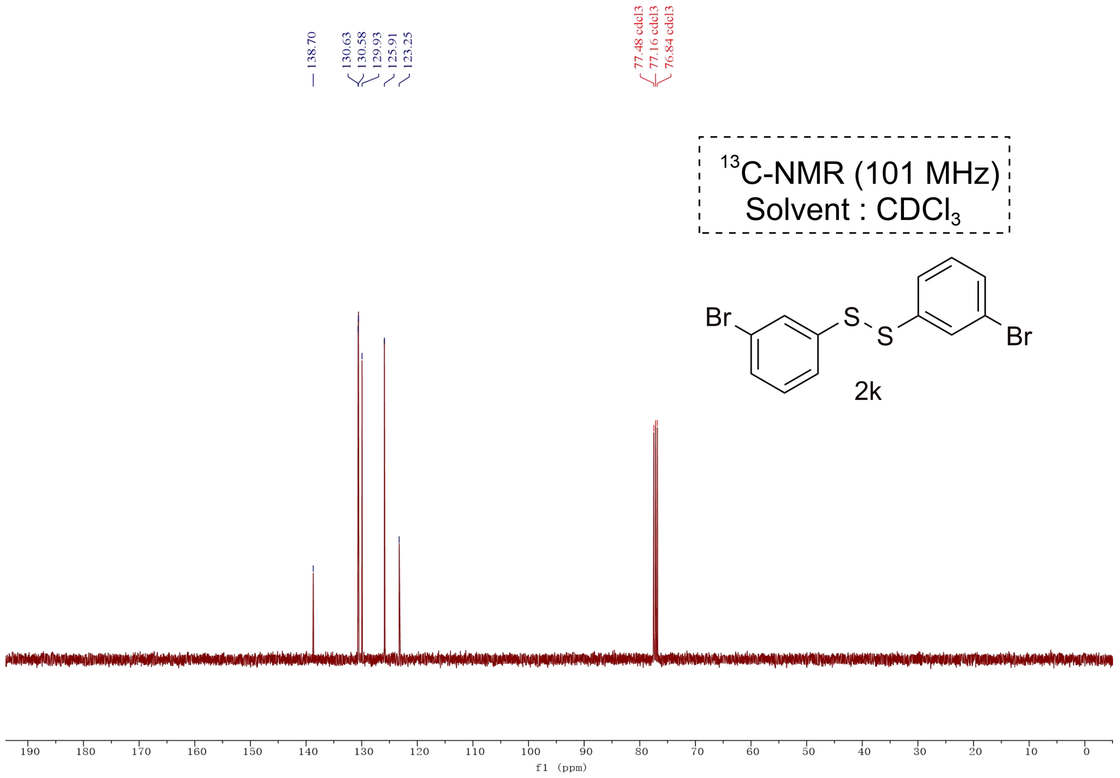
138.70
130.63
130.58
129.93
125.91
123.25
77.48 cdcl3
77.16 cdcl3
76.84 cdcl3
^{13}C-NMR (101 MHz)
Solvent : $CDCl_3$
Br
S
S
Br
2k
f1 (ppm)

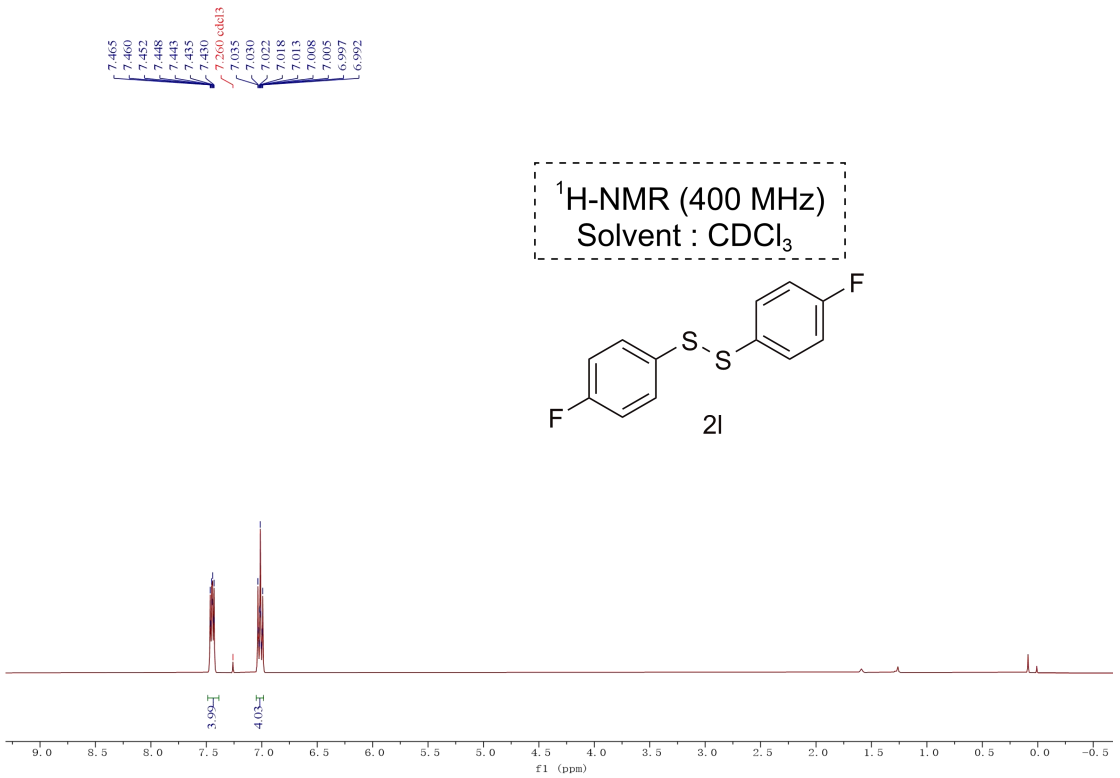
7.465
7.460
7.452
7.448
7.443
7.435
7.430
7.260 cdcl3
7.035
7.030
7.022
7.018
7.013
7.008
7.005
6.997
6.992
1H-NMR (400 MHz)
Solvent : CDCl3
2l
3.99
4.03
f1 (ppm)

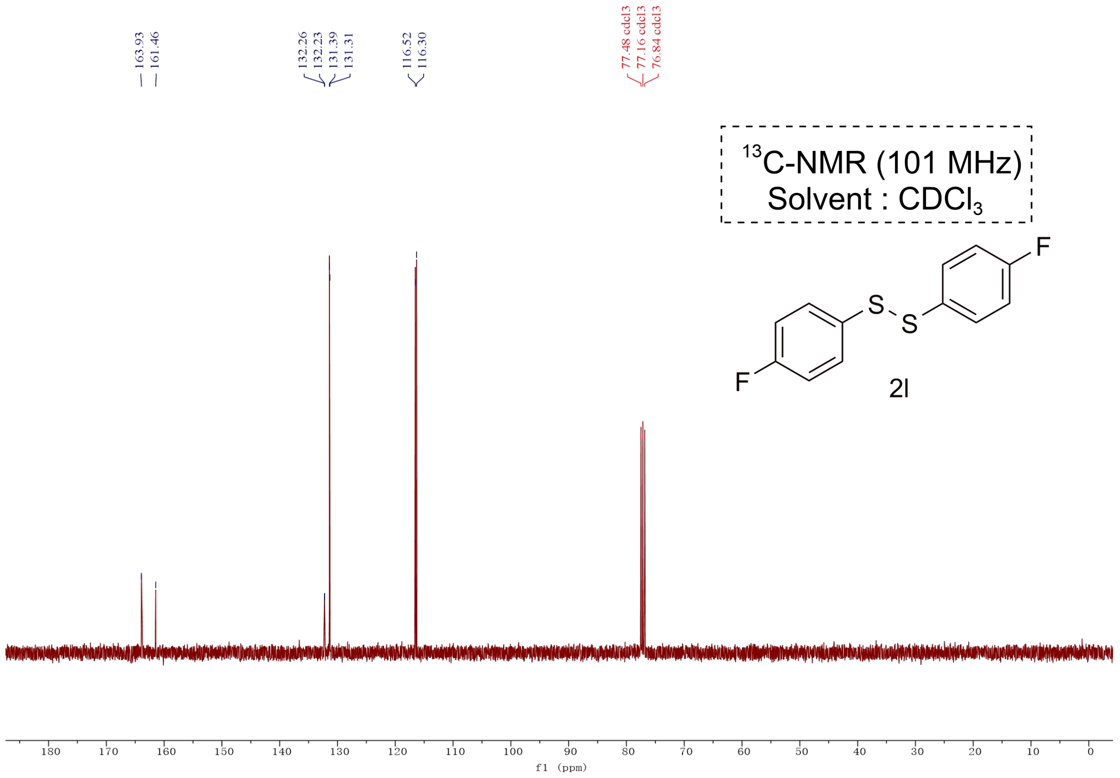
163.93
161.46
132.26
132.23
131.39
131.31
116.52
116.30
77.48 cdcl3
77.16 cdcl3
76.84 cdcl3
13C-NMR (101 MHz)
Solvent : CDCl3
2l
f1 (ppm)

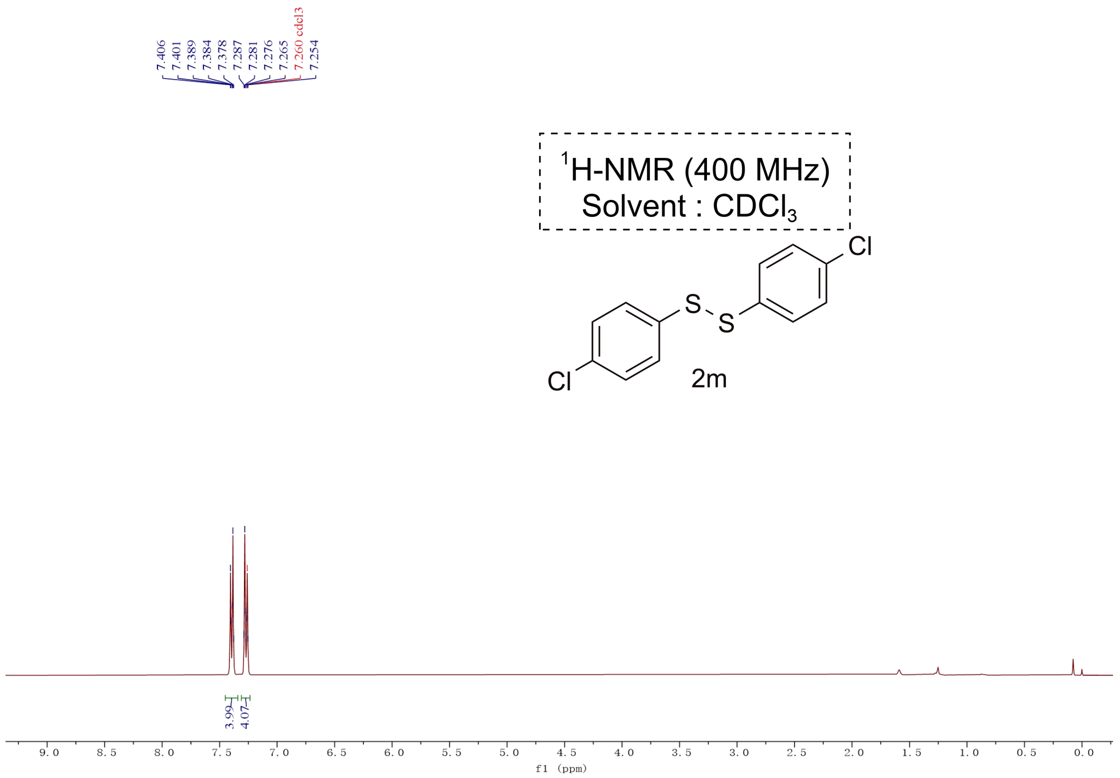

7.406
7.401
7.389
7.384
7.378
7.287
7.281
7.276
7.265
7.260 cdcl3
7.254
^{1}H-NMR (400 MHz)
Solvent : $CDCl_3$
Cl
S
S
Cl
2m
3.99
4.07
f1 (ppm)

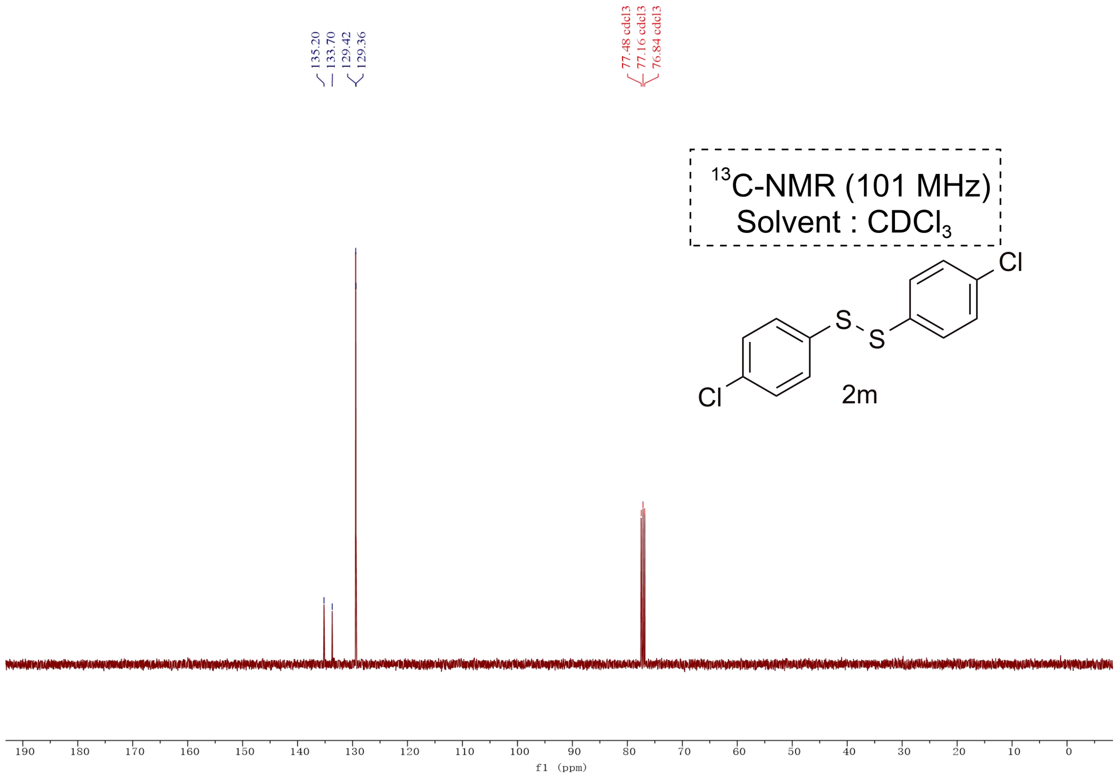

135.20
133.70
129.42
129.36
77.48 cdcl3
77.16 cdcl3
76.84 cdcl3
^{13}C-NMR (101 MHz)
Solvent : $CDCl_3$
Cl
S
S
Cl
2m
f1 (ppm)

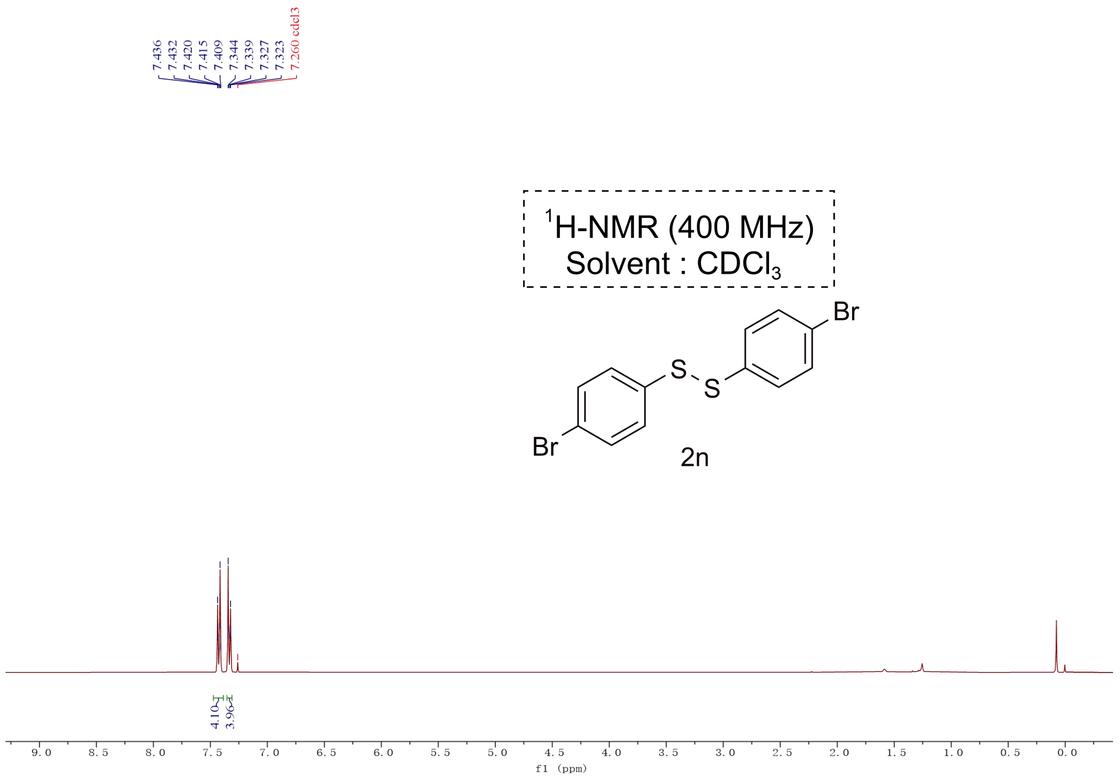

7.436
7.432
7.420
7.415
7.409
7.344
7.339
7.327
7.323
7.260 cdcl3
^{1}H-NMR (400 MHz)
Solvent : $CDCl_3$
Br
S
S
Br
2n
4.10
3.96
f1 (ppm)

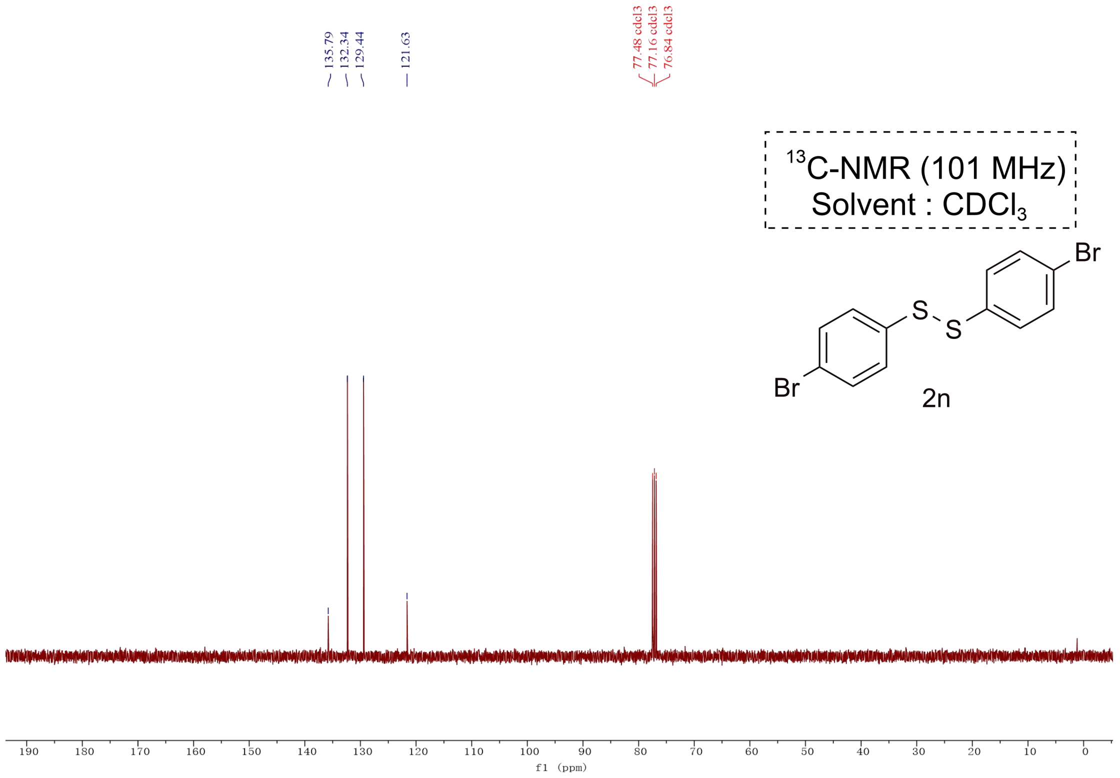

135.79
132.34
129.44
121.63
77.48 cdcl3
77.16 cdcl3
76.84 cdcl3
^{13}C-NMR (101 MHz)
Solvent : $CDCl_3$
Br
S
S
Br
2n
f1 (ppm)

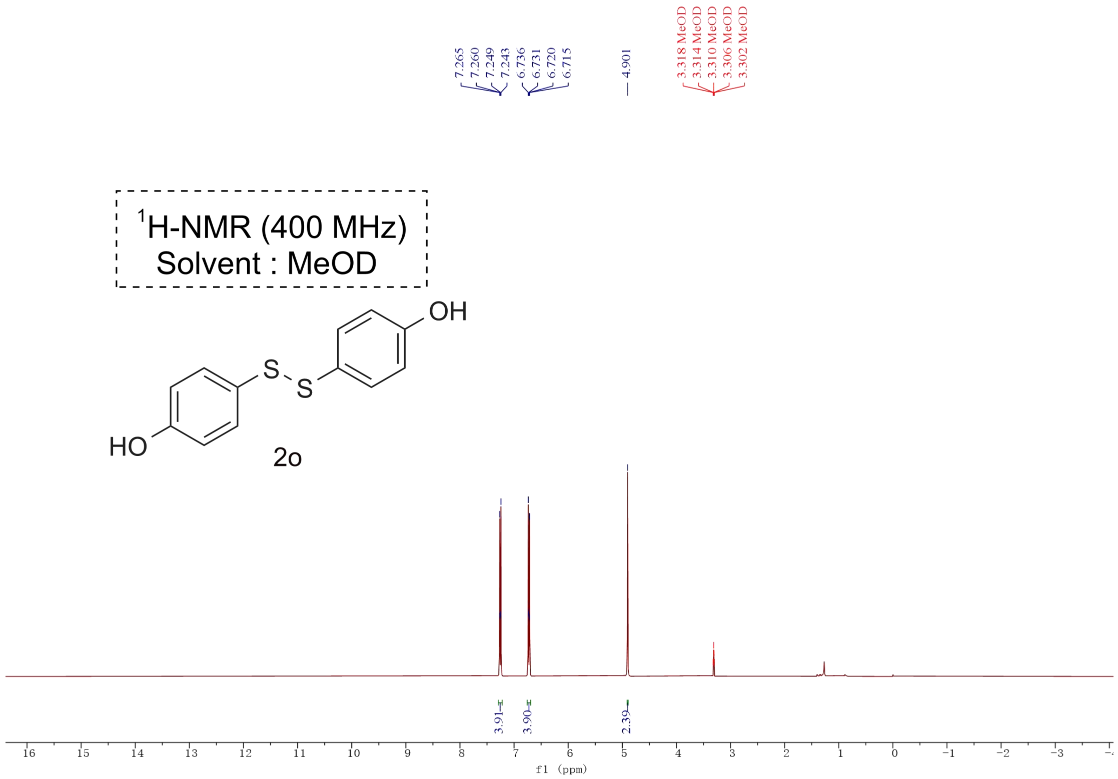

7.265
7.260
7.249
7.243
6.736
6.731
6.720
6.715
4.901
3.318 MeOD
3.314 MeOD
3.310 MeOD
3.306 MeOD
3.302 MeOD
^{1}H-NMR (400 MHz)
Solvent : MeOD
OH
S
S
HO
2o
3.91
3.90
2.39
f1 (ppm)

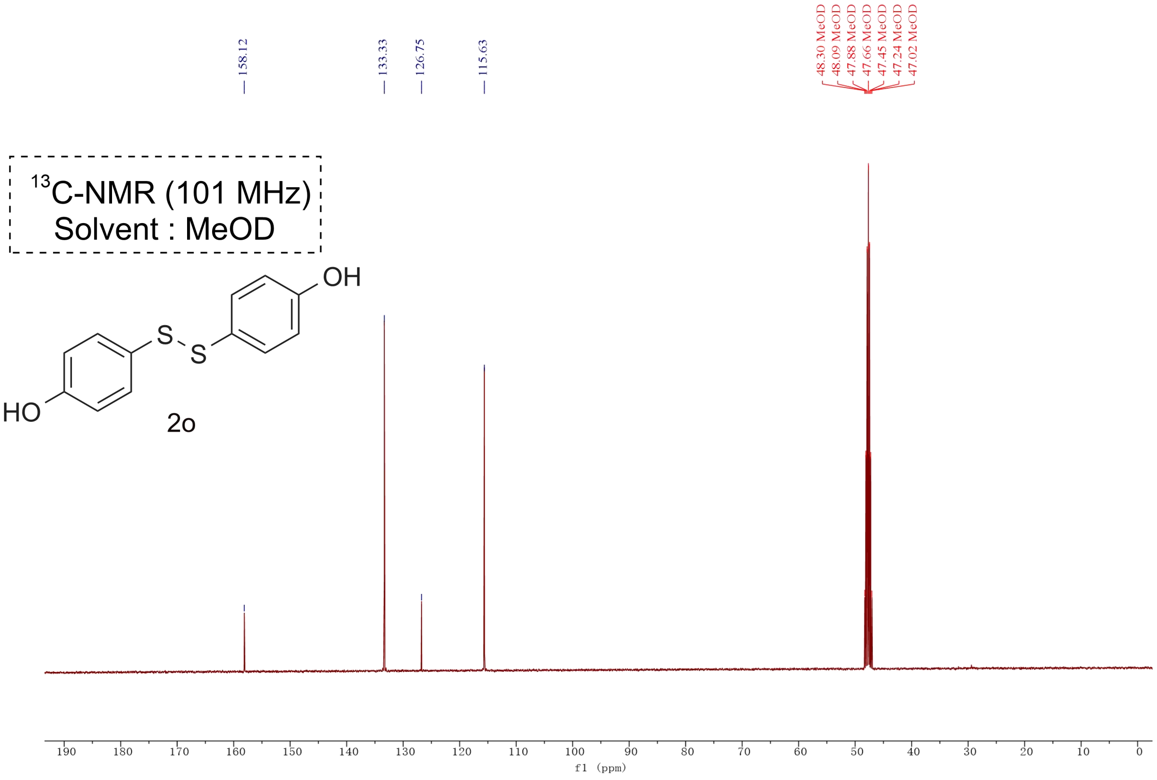

158.12
133.33
126.75
115.63
48.30 MeOD
48.09 MeOD
47.88 MeOD
47.66 MeOD
47.45 MeOD
47.24 MeOD
47.02 MeOD
^{13}C-NMR (101 MHz)
Solvent : MeOD
OH
S
S
HO
2o
f1 (ppm)

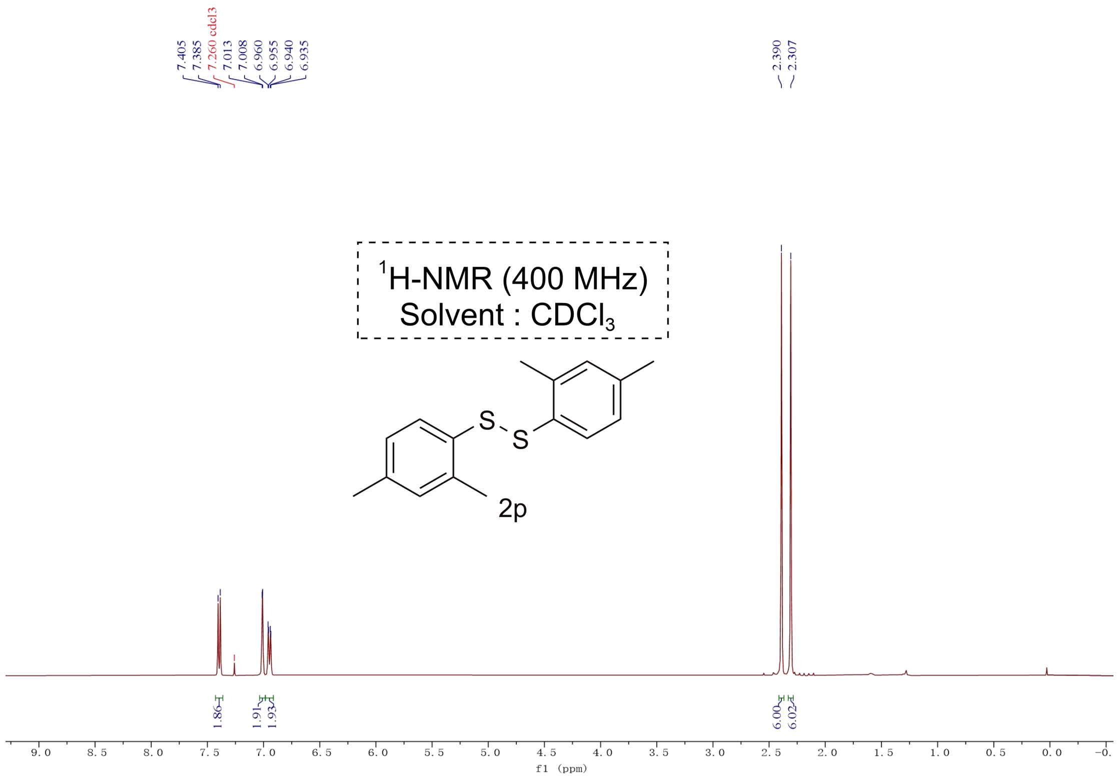
7.405
7.385
7.260 cdcl3
7.013
7.008
6.960
6.955
6.940
6.935
2.390
2.307
^{1}H-NMR (400 MHz)
Solvent : $CDCl_3$
S
S
2p
1.86
1.91
1.93
6.00
6.02
f1 (ppm)

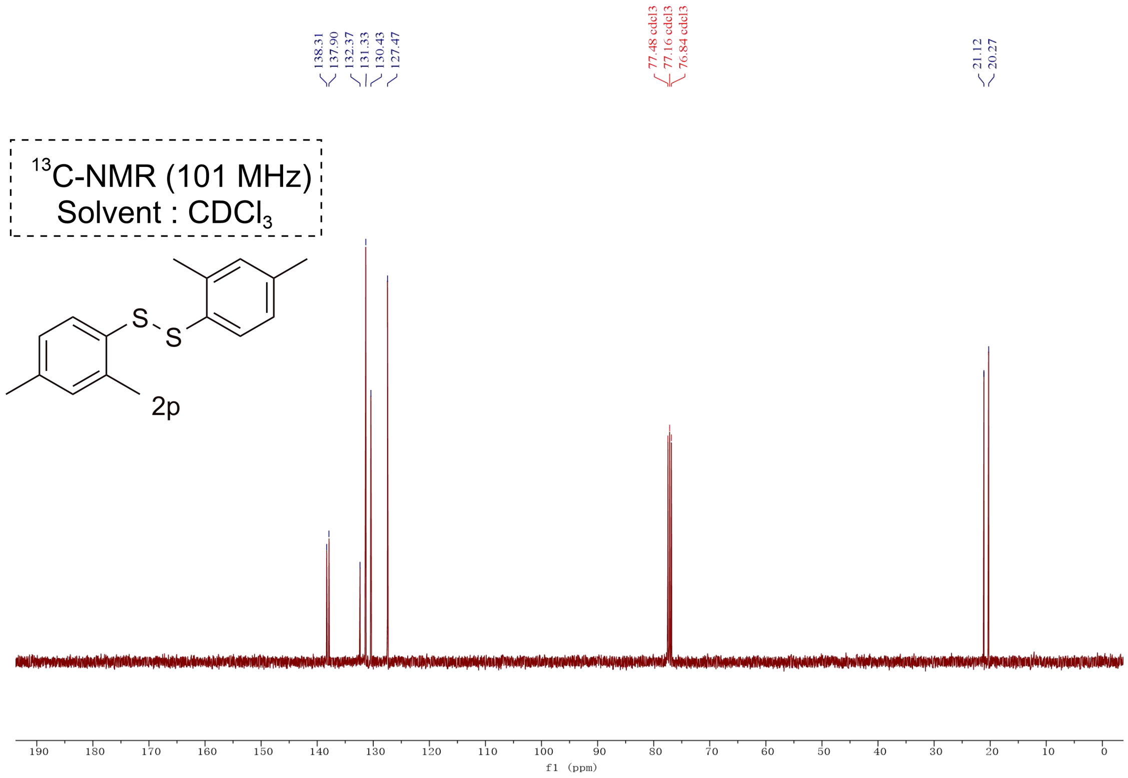
138.31
137.90
132.37
131.33
130.43
127.47
77.48 cdcl3
77.16 cdcl3
76.84 cdcl3
21.12
20.27
^{13}C-NMR (101 MHz)
Solvent : $CDCl_3$
S
S
2p
f1 (ppm)

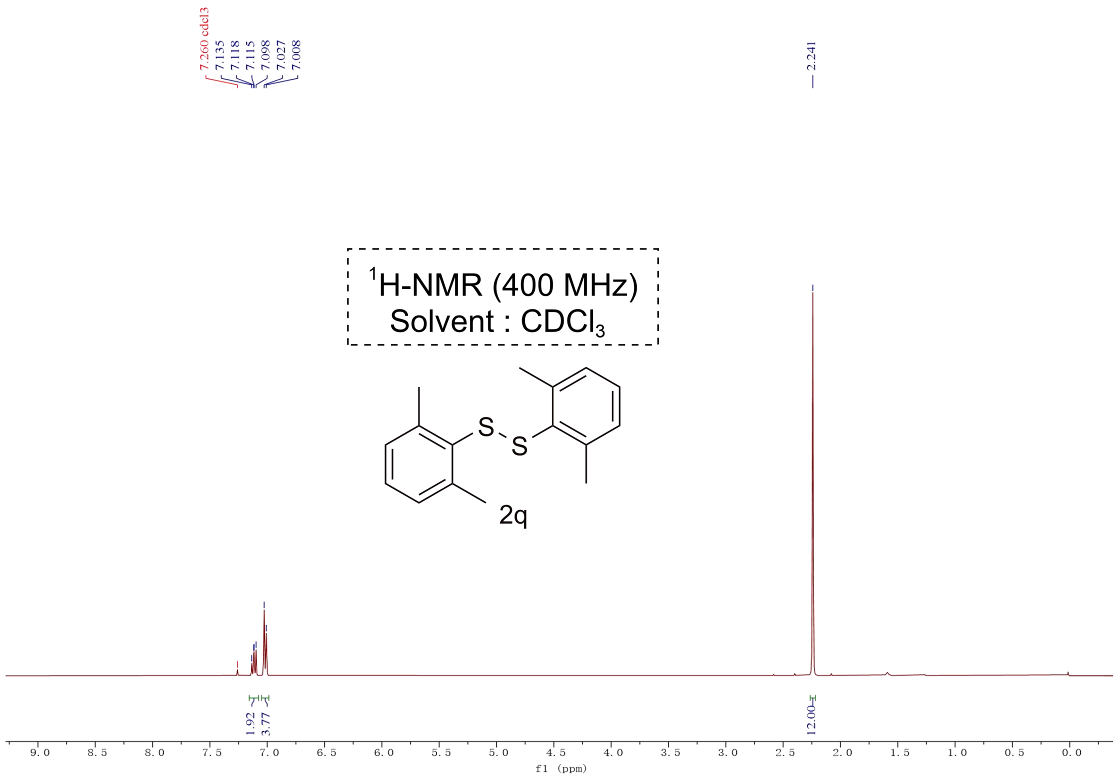

7.260 cdcl3
7.135
7.118
7.115
7.098
7.027
7.008
2.241
1H-NMR (400 MHz)
Solvent : CDCl3
S
S
2q
1.92
3.77
12.00
9.0 8.5 8.0 7.5 7.0 6.5 6.0 5.5 5.0 4.5 4.0 3.5 3.0 2.5 2.0 1.5 1.0 0.5 0.0
f1 (ppm)

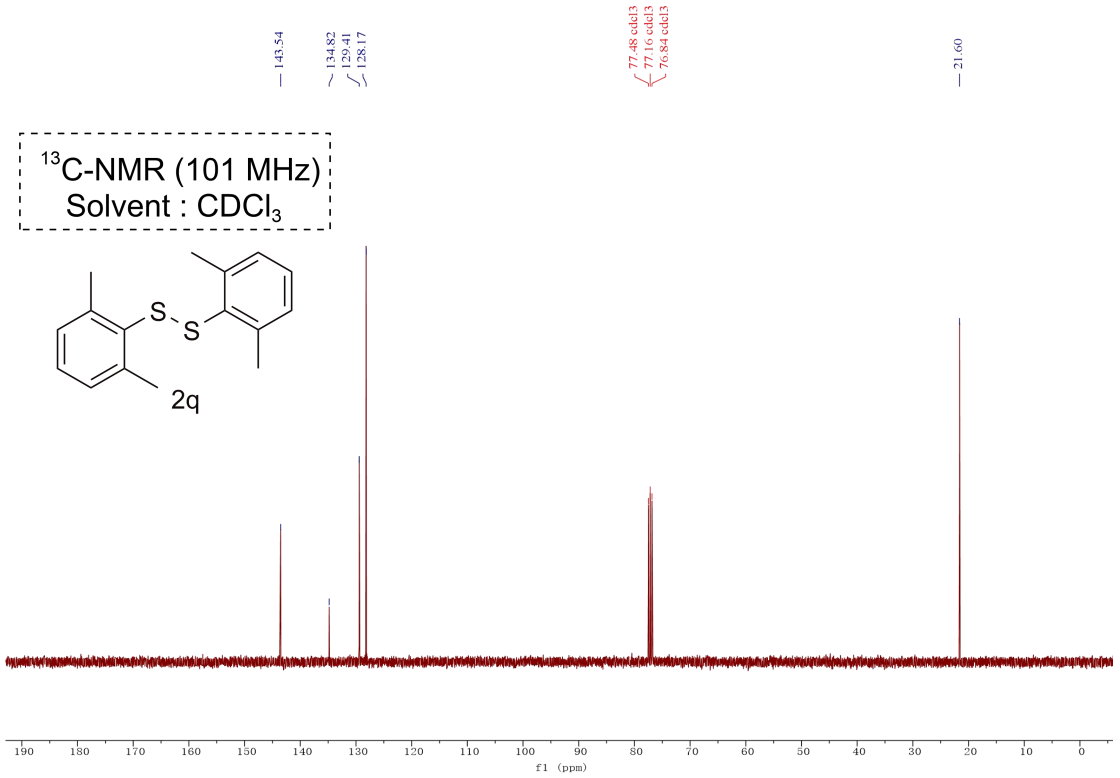

143.54
134.82
129.41
128.17
77.48 cdcl3
77.16 cdcl3
76.84 cdcl3
21.60
13C-NMR (101 MHz)
Solvent : CDCl3
S
S
2q
190 180 170 160 150 140 130 120 110 100 90 80 70 60 50 40 30 20 10 0
f1 (ppm)

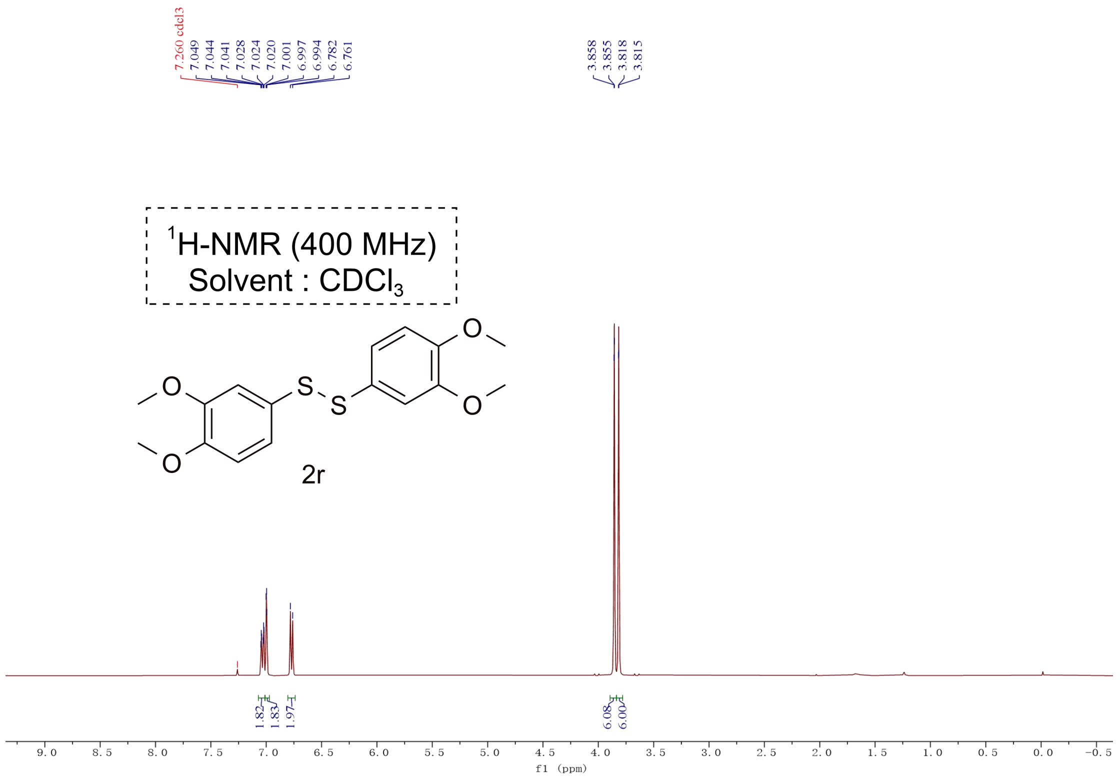
7.260 cdcl3
7.049
7.044
7.041
7.028
7.024
7.020
7.001
6.997
6.994
6.782
6.761
3.858
3.855
3.818
3.815
^{1}H-NMR (400 MHz)
Solvent : $CDCl_3$
S
S
O
O
O
O
2r
1.82
1.83
1.97
6.08
6.00
9.0 8.5 8.0 7.5 7.0 6.5 6.0 5.5 5.0 4.5 4.0 3.5 3.0 2.5 2.0 1.5 1.0 0.5 0.0 -0.5
f1 (ppm)

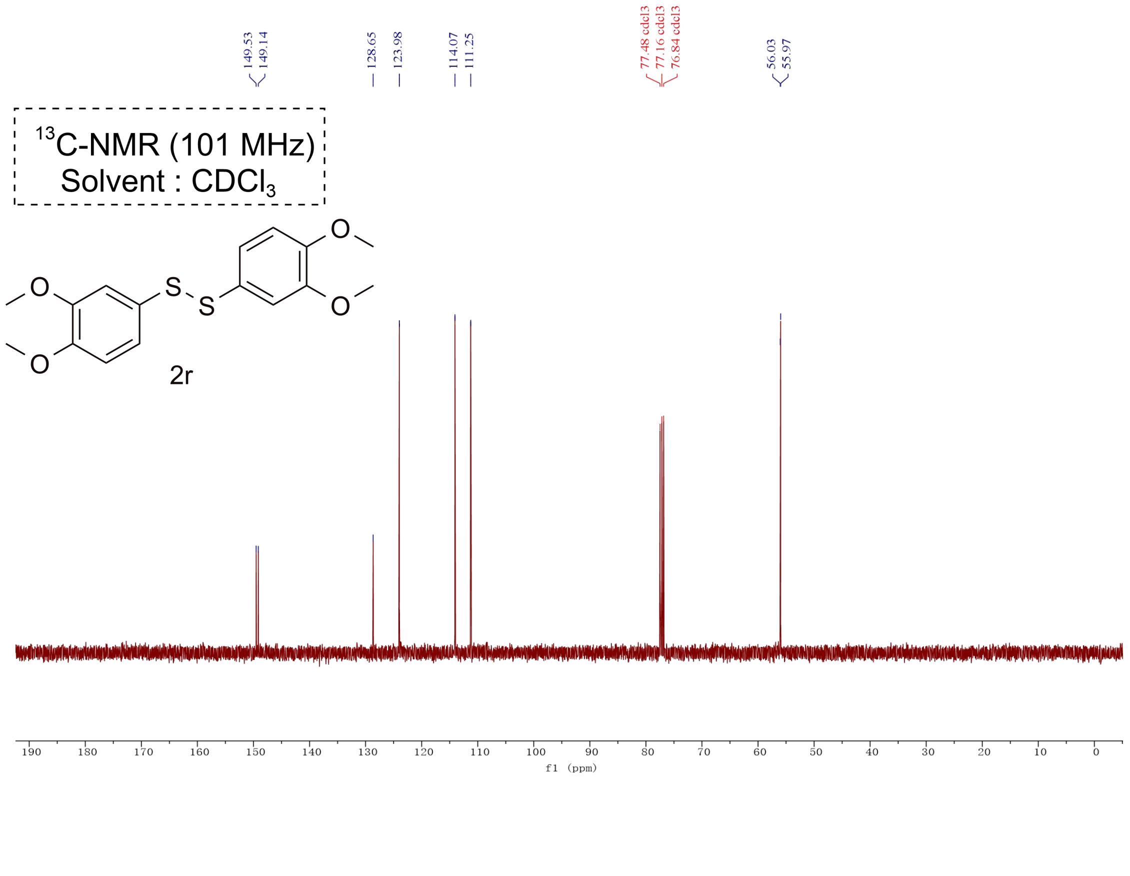
149.53
149.14
128.65
123.98
114.07
111.25
77.48 cdcl3
77.16 cdcl3
76.84 cdcl3
56.03
55.97
^{13}C-NMR (101 MHz)
Solvent : $CDCl_3$
S
S
O
O
O
O
2r
190 180 170 160 150 140 130 120 110 100 90 80 70 60 50 40 30 20 10 0
f1 (ppm)

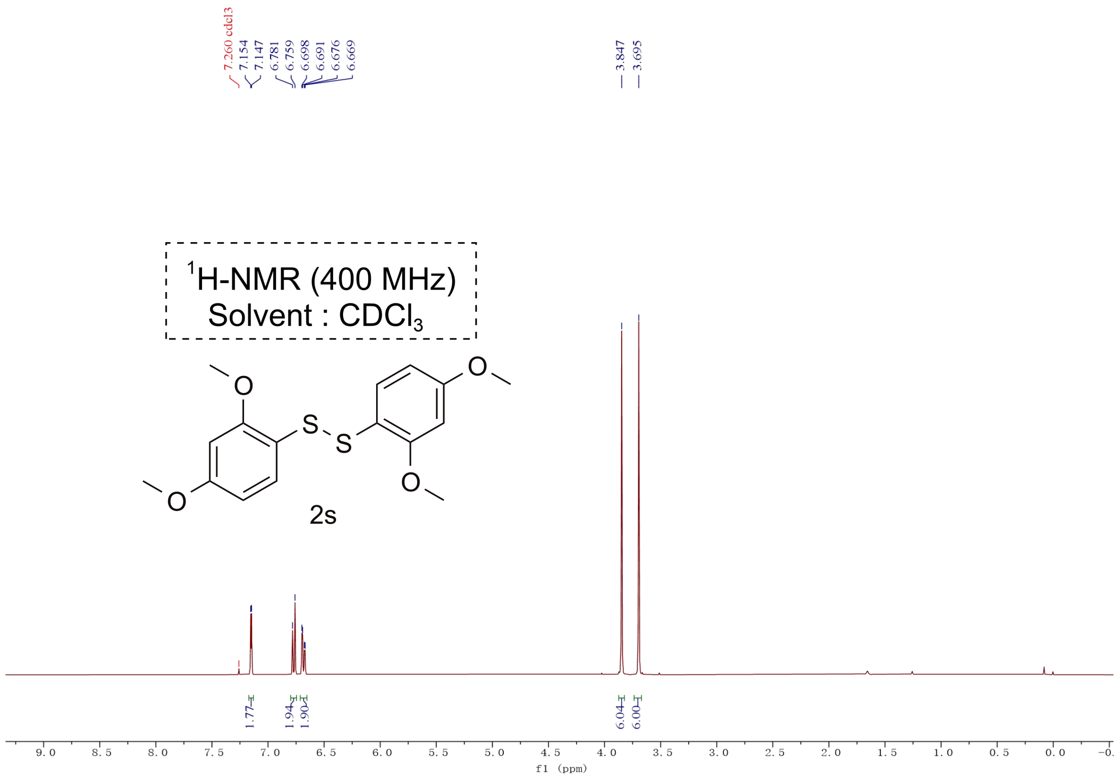
7.260 cdcl3
7.154
7.147
6.781
6.759
6.698
6.691
6.676
6.669
3.847
3.695
1H-NMR (400 MHz)
Solvent : CDCl3
2s
1.77
1.94
1.90
6.04
6.00
f1 (ppm)

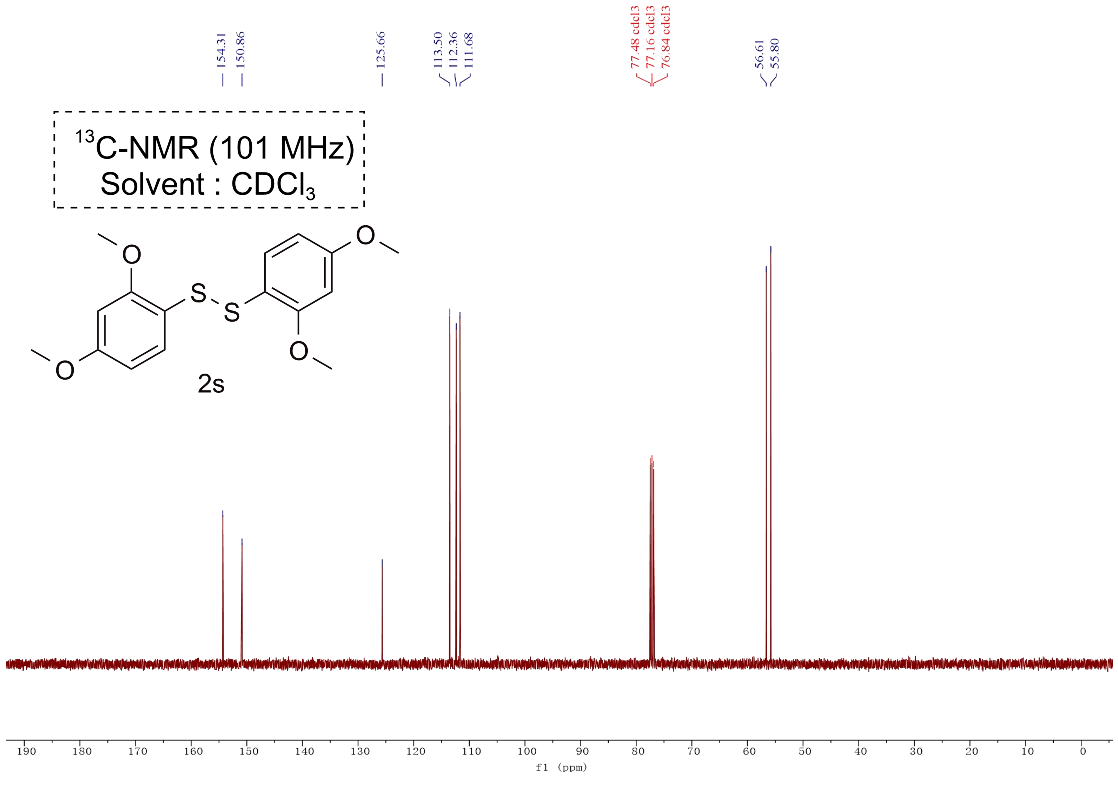
154.31
150.86
125.66
113.50
112.36
111.68
77.48 cdcl3
77.16 cdcl3
76.84 cdcl3
56.61
55.80
13C-NMR (101 MHz)
Solvent : CDCl3
2s
f1 (ppm)

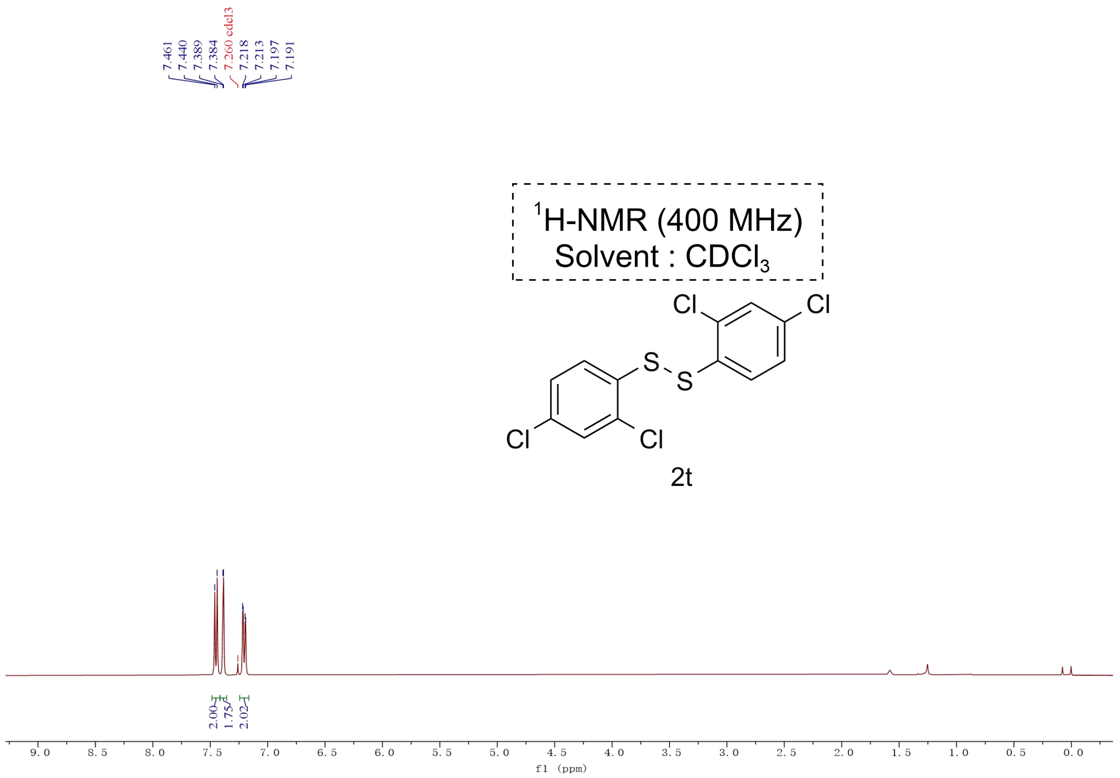
7.461
7.440
7.389
7.384
7.260 cdcl3
7.218
7.213
7.197
7.191
1H-NMR (400 MHz)
Solvent : CDCl3
Cl
Cl
S
S
Cl
Cl
2t
2.00
1.75
2.02
9.0 8.5 8.0 7.5 7.0 6.5 6.0 5.5 5.0 4.5 4.0 3.5 3.0 2.5 2.0 1.5 1.0 0.5 0.0
f1 (ppm)

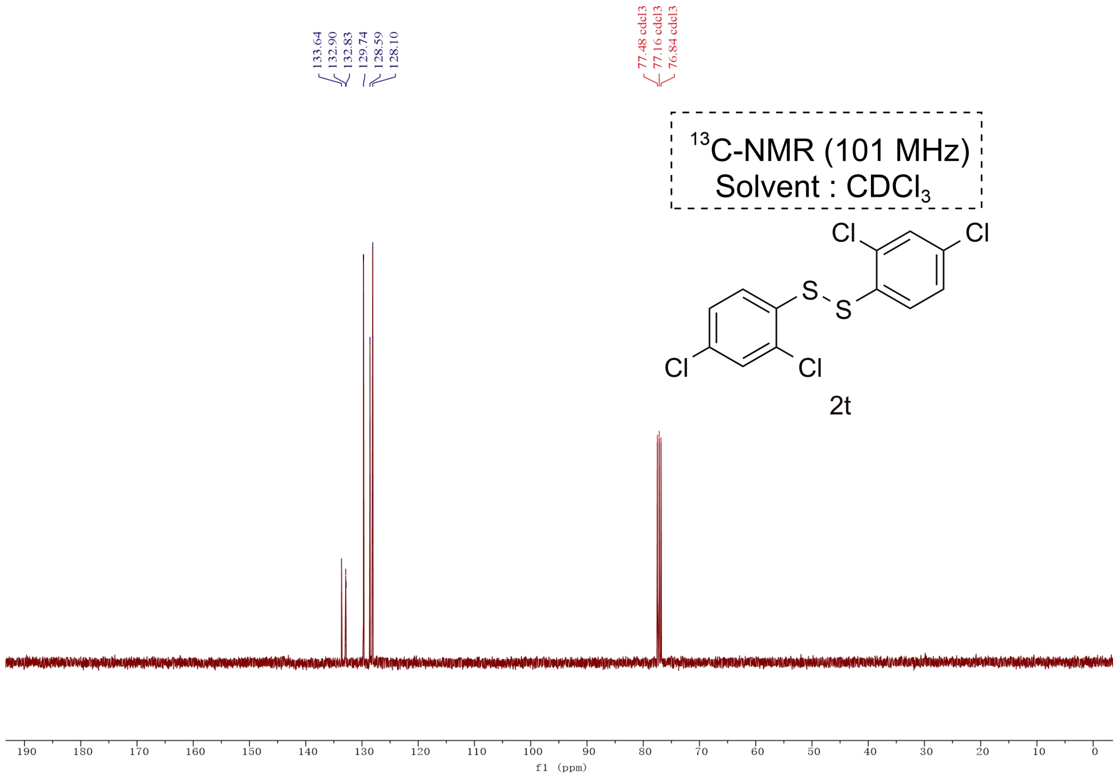
133.64
132.90
132.83
129.74
128.59
128.10
77.48 cdcl3
77.16 cdcl3
76.84 cdcl3
13C-NMR (101 MHz)
Solvent : CDCl3
Cl
Cl
S
S
Cl
Cl
2t
190 180 170 160 150 140 130 120 110 100 90 80 70 60 50 40 30 20 10 0
f1 (ppm)

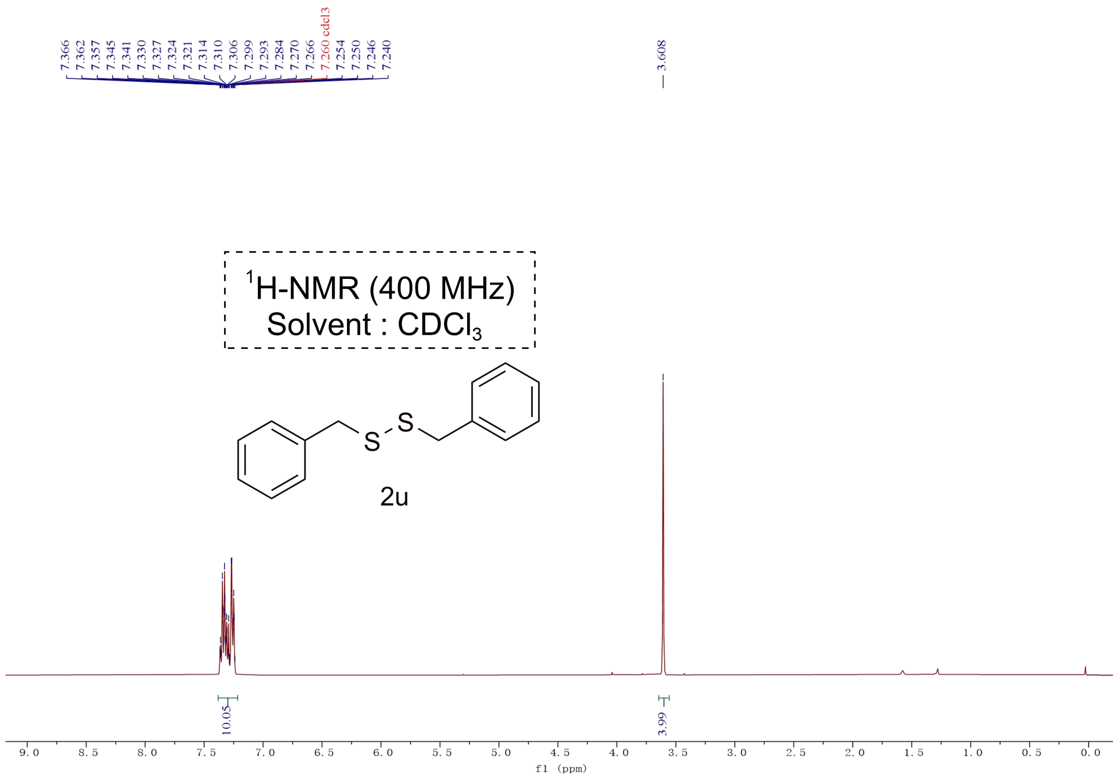

7.366
7.362
7.357
7.345
7.341
7.330
7.327
7.324
7.321
7.314
7.310
7.306
7.299
7.293
7.284
7.270
7.266
7.260 cdcl3
7.254
7.250
7.246
7.240
3.608
^{1}H-NMR (400 MHz)
Solvent : CDCl$_3$
S
S
2u
10.05
3.99
f1 (ppm)

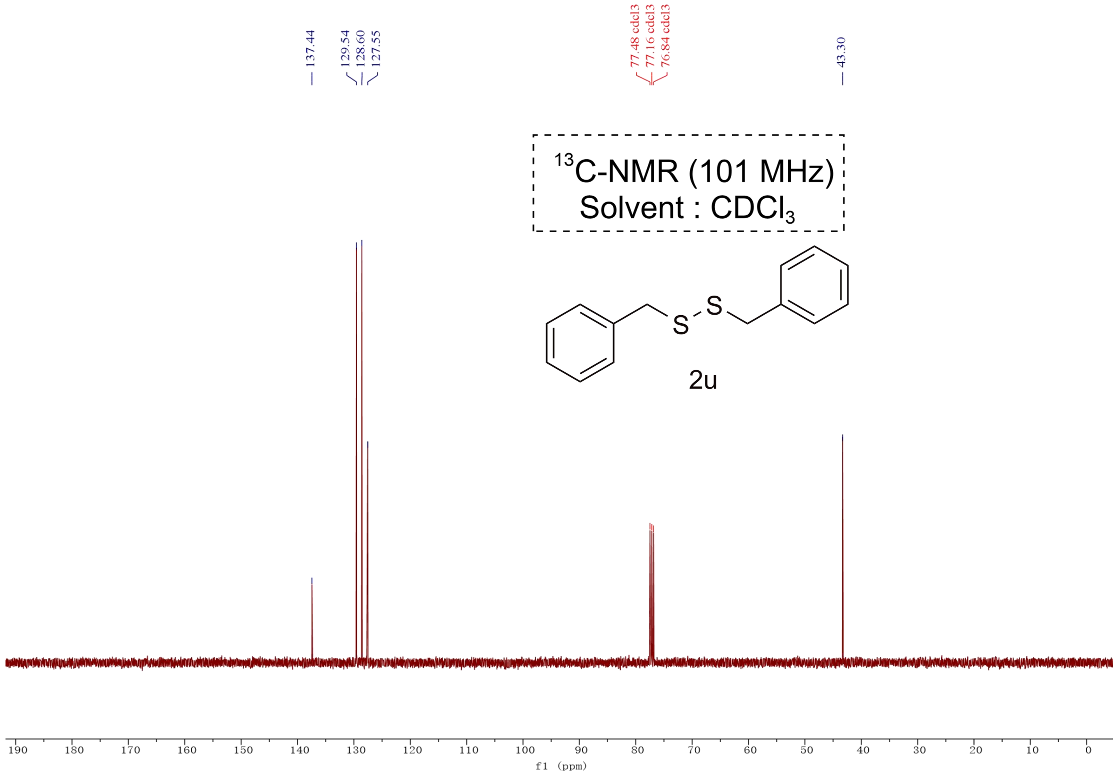

137.44
129.54
128.60
127.55
77.48 cdcl3
77.16 cdcl3
76.84 cdcl3
43.30
^{13}C-NMR (101 MHz)
Solvent : CDCl$_3$
S
S
2u
f1 (ppm)

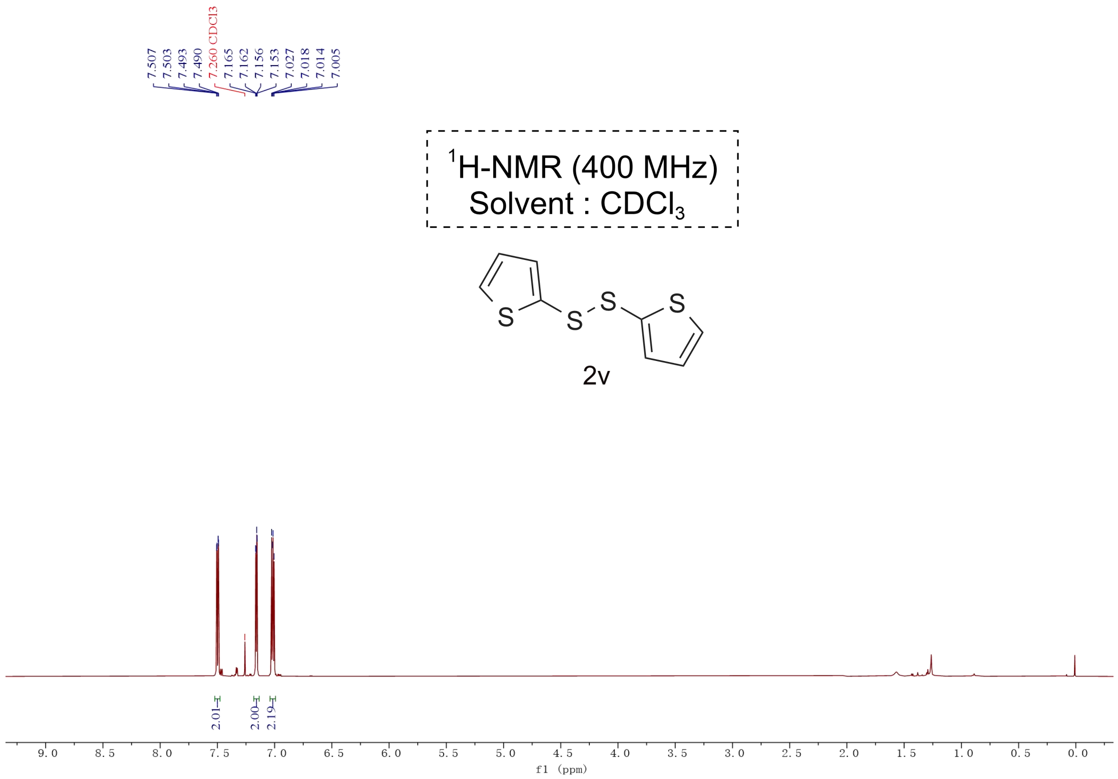

^{1}H-NMR (400 MHz)
Solvent : $CDCl_3$

2v

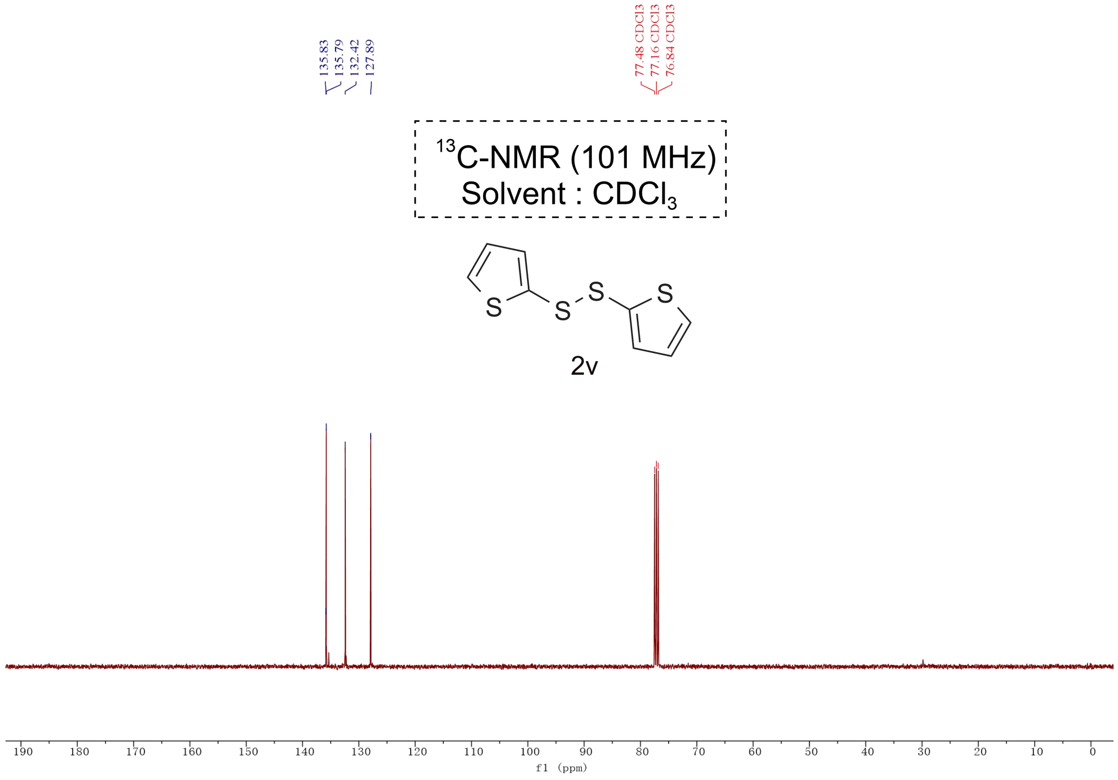

^{13}C-NMR (101 MHz)
Solvent : $CDCl_3$

2v

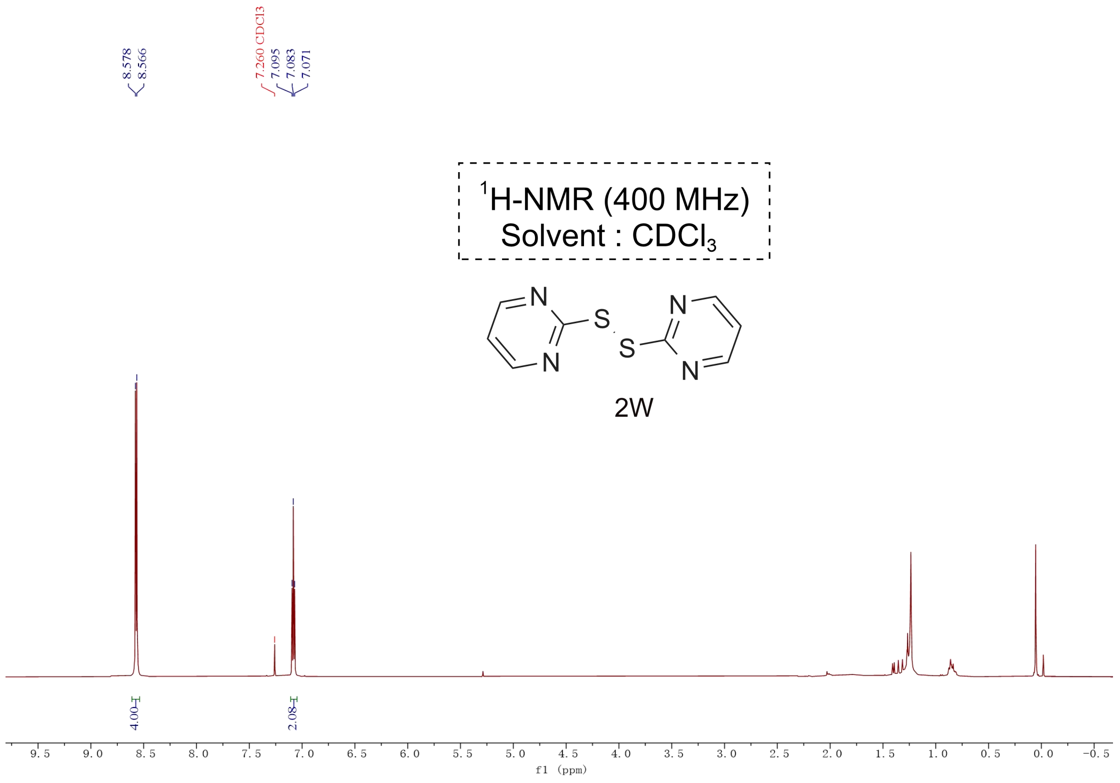

8.578
8.566
7.260 CDCl3
7.095
7.083
7.071
^{1}H-NMR (400 MHz)
Solvent : CDCl$_3$
N
S
S
N
N
N
2W
4.00
2.08
f1 (ppm)

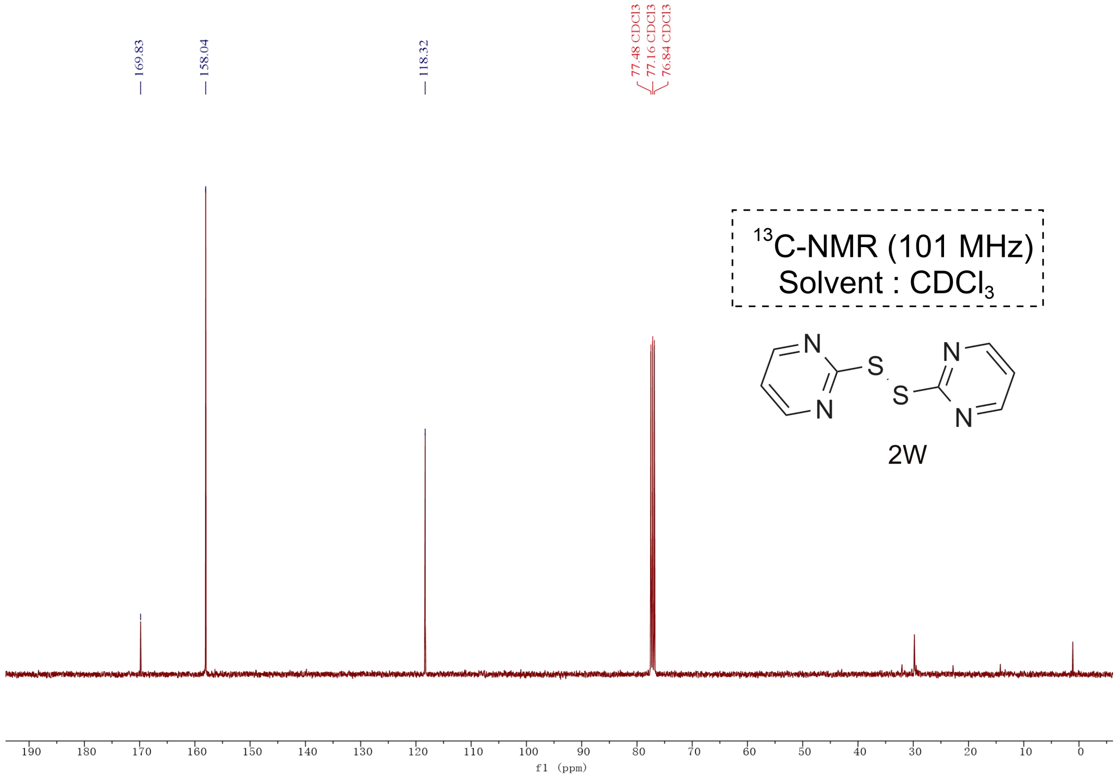

169.83
158.04
118.32
77.48 CDCl3
77.16 CDCl3
76.84 CDCl3
^{13}C-NMR (101 MHz)
Solvent : CDCl$_3$
N
S
S
N
N
N
2W
f1 (ppm)

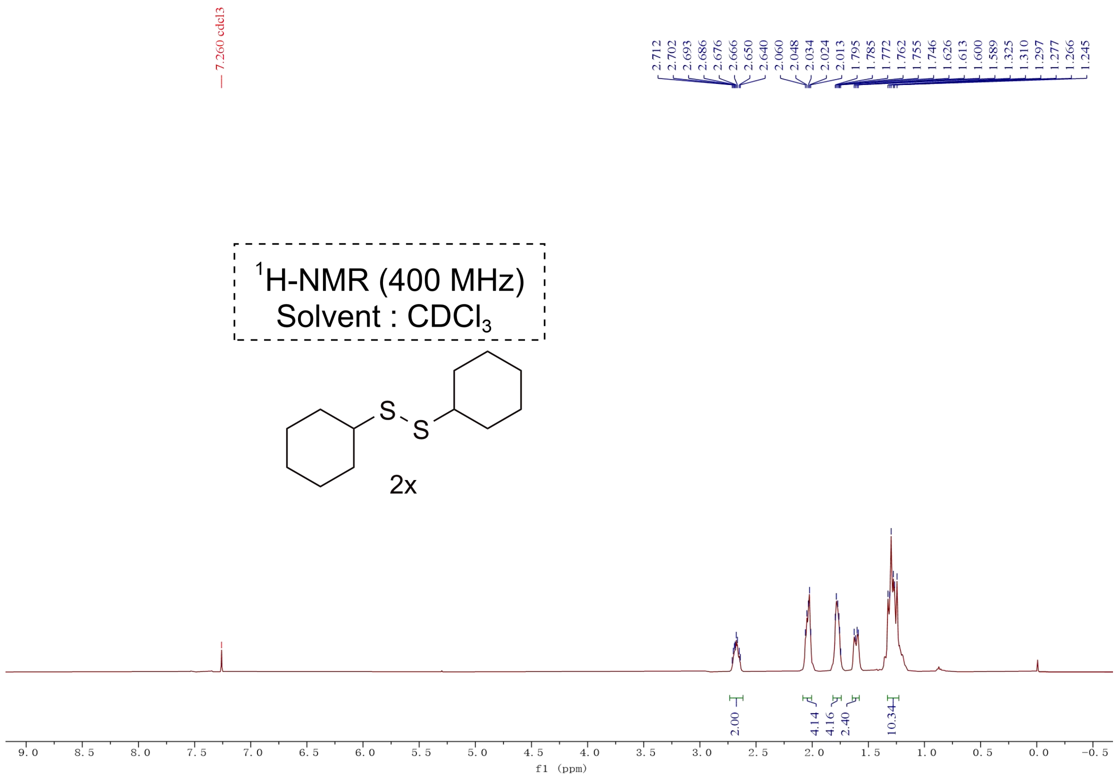
7.260 cdcl3
2.712 2.702 2.693 2.686 2.676 2.666 2.650 2.640 2.060 2.048 2.034 2.024 2.013 1.795 1.785 1.772 1.762 1.755 1.746 1.626 1.613 1.600 1.589 1.325 1.310 1.297 1.277 1.266 1.245
^{1}H-NMR (400 MHz)
Solvent : $CDCl_3$
S
S
2x
2.00
4.14
4.16
2.40
10.34
f1 (ppm)

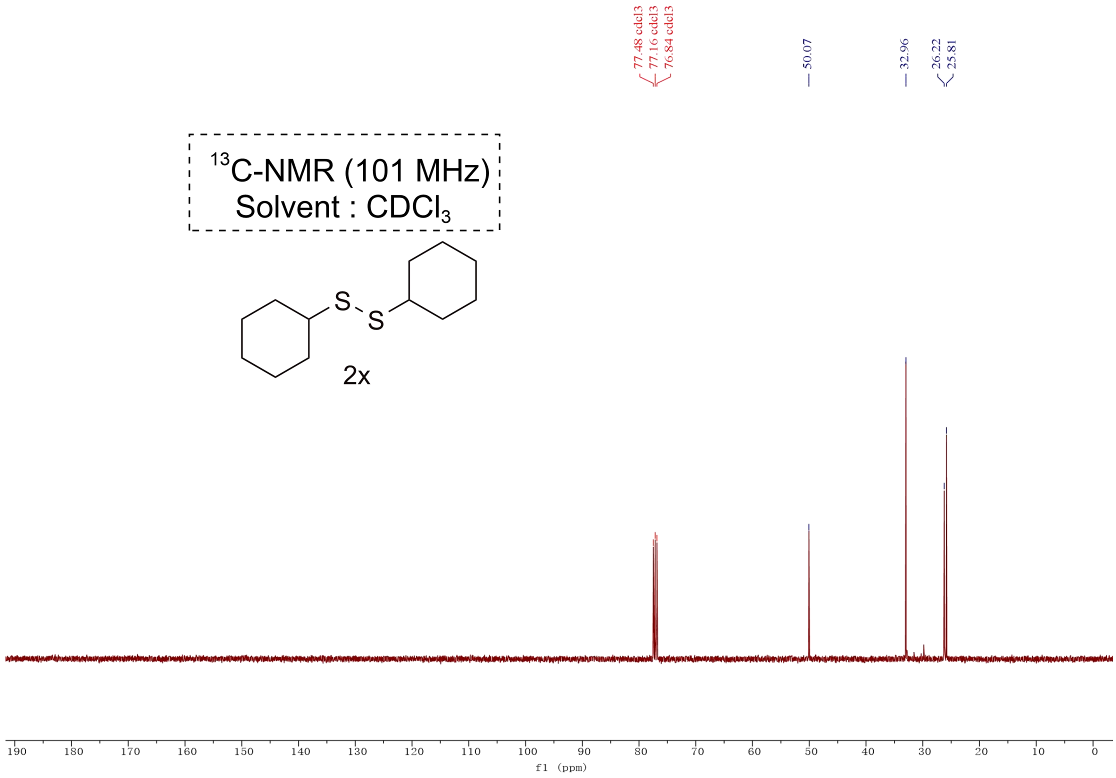
77.48 cdcl3
77.16 cdcl3
76.84 cdcl3
50.07
32.96
26.22
25.81
^{13}C-NMR (101 MHz)
Solvent : $CDCl_3$
S
S
2x
f1 (ppm)

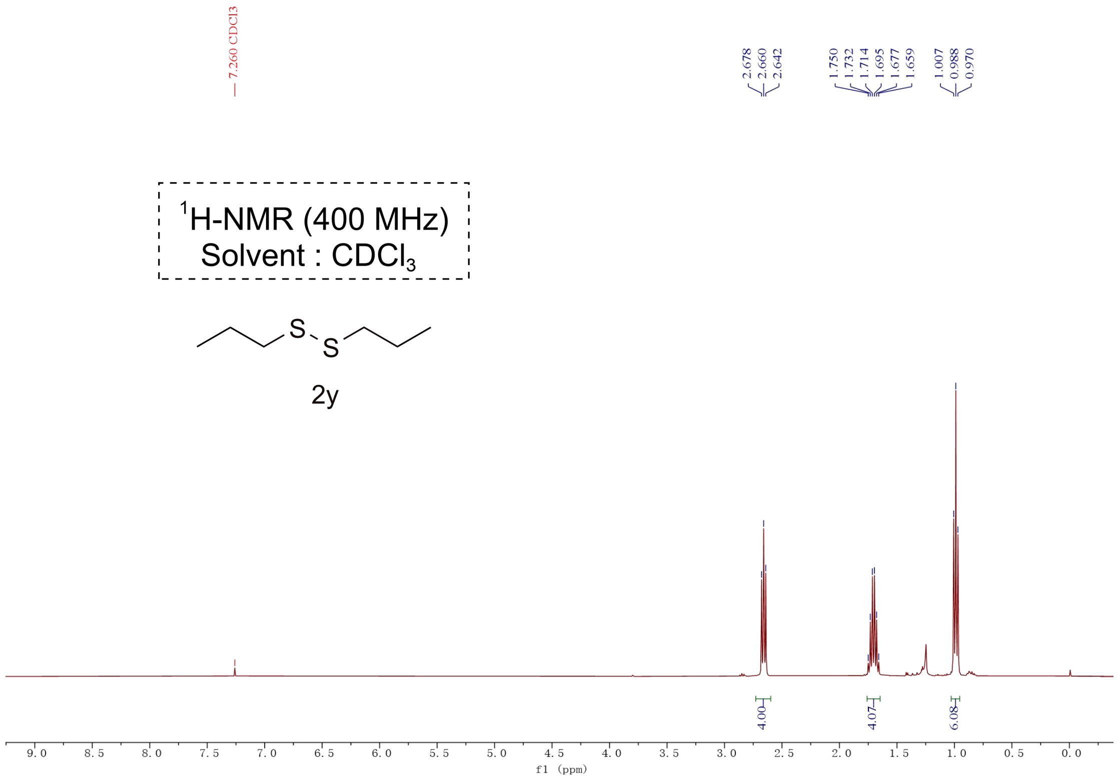
7.260 CDCl3
2.678
2.660
2.642
1.750
1.732
1.714
1.695
1.677
1.659
1.007
0.988
0.970
1H-NMR (400 MHz)
Solvent : CDCl3
S
S
2y
4.00
4.07
6.08
f1 (ppm)

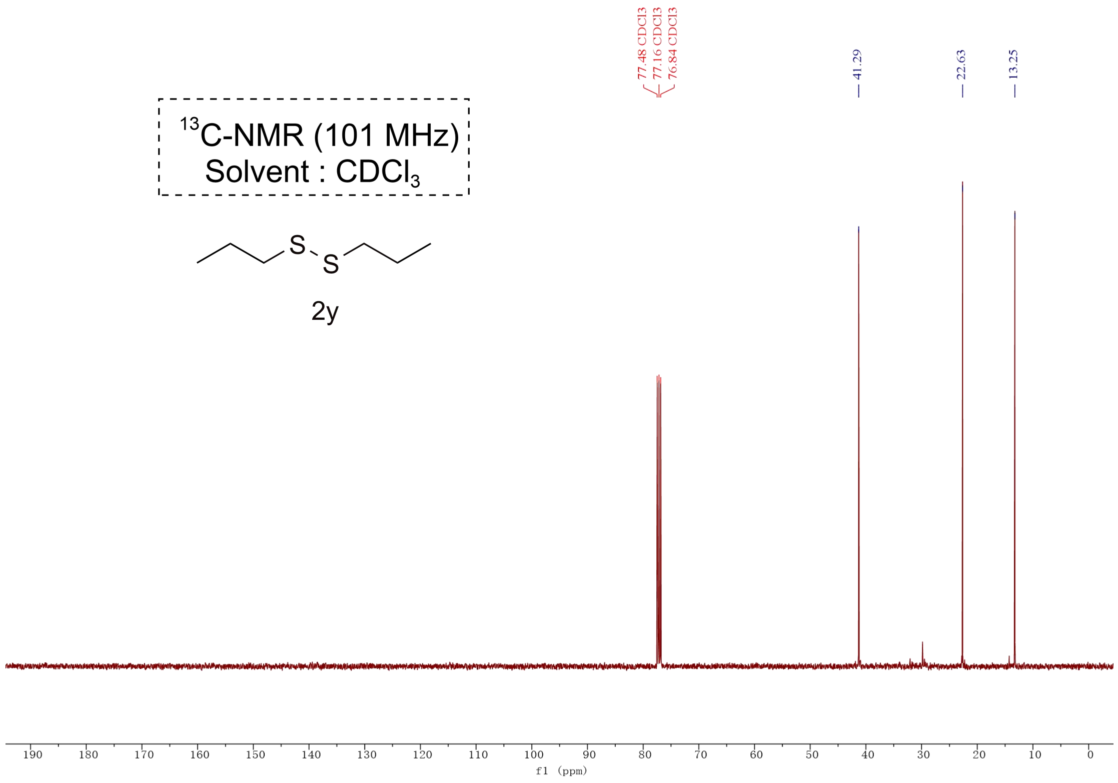
77.48 CDCl3
77.16 CDCl3
76.84 CDCl3
41.29
22.63
13.25
13C-NMR (101 MHz)
Solvent : CDCl3
S
S
2y
f1 (ppm)

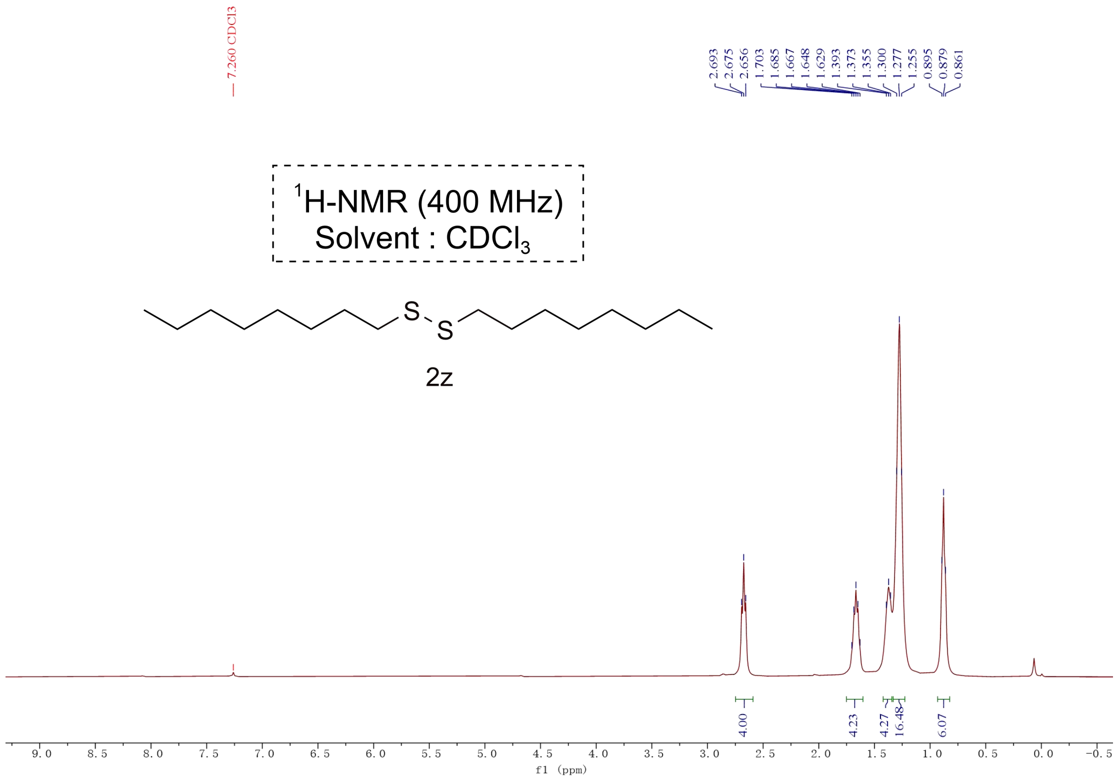

7.260 CDCl3
2.693
2.675
2.656
1.703
1.685
1.667
1.648
1.629
1.393
1.373
1.355
1.300
1.277
1.255
0.895
0.879
0.861
1H-NMR (400 MHz)
Solvent : CDCl3
S
S
2z
4.00
4.23
4.27
16.48
6.07
f1 (ppm)

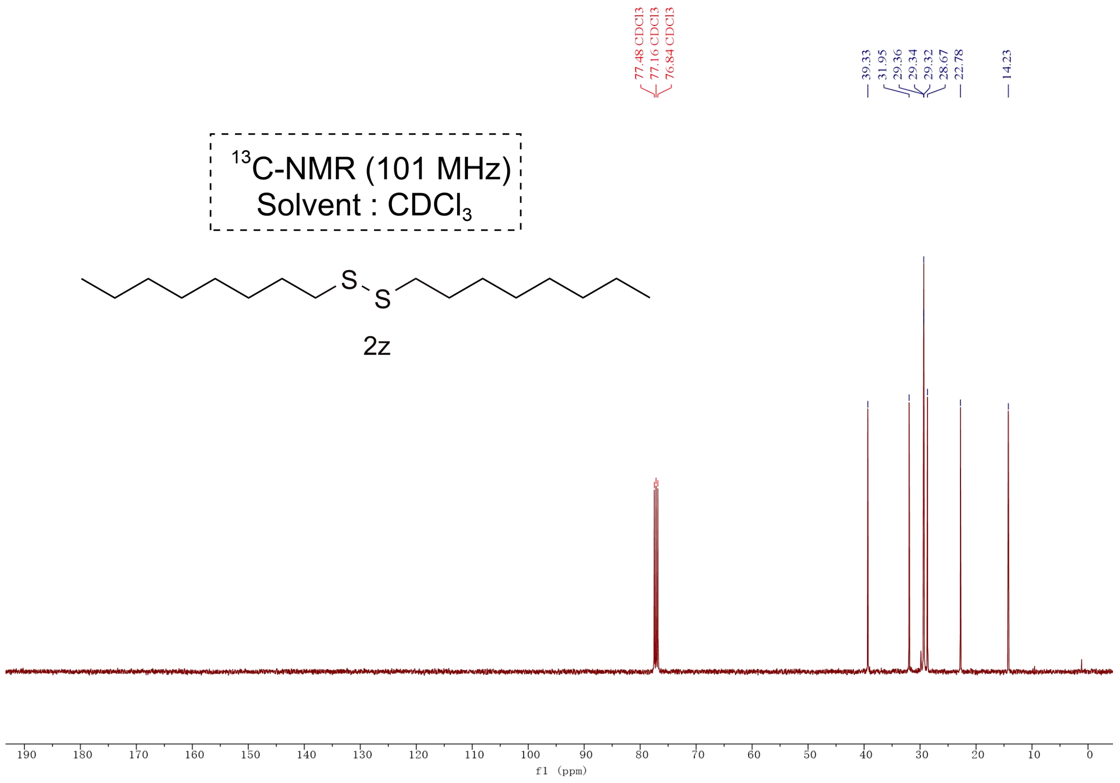

77.48 CDCl3
77.16 CDCl3
76.84 CDCl3
39.33
31.95
29.36
29.34
29.32
28.67
22.78
14.23
13C-NMR (101 MHz)
Solvent : CDCl3
S
S
2z
f1 (ppm)

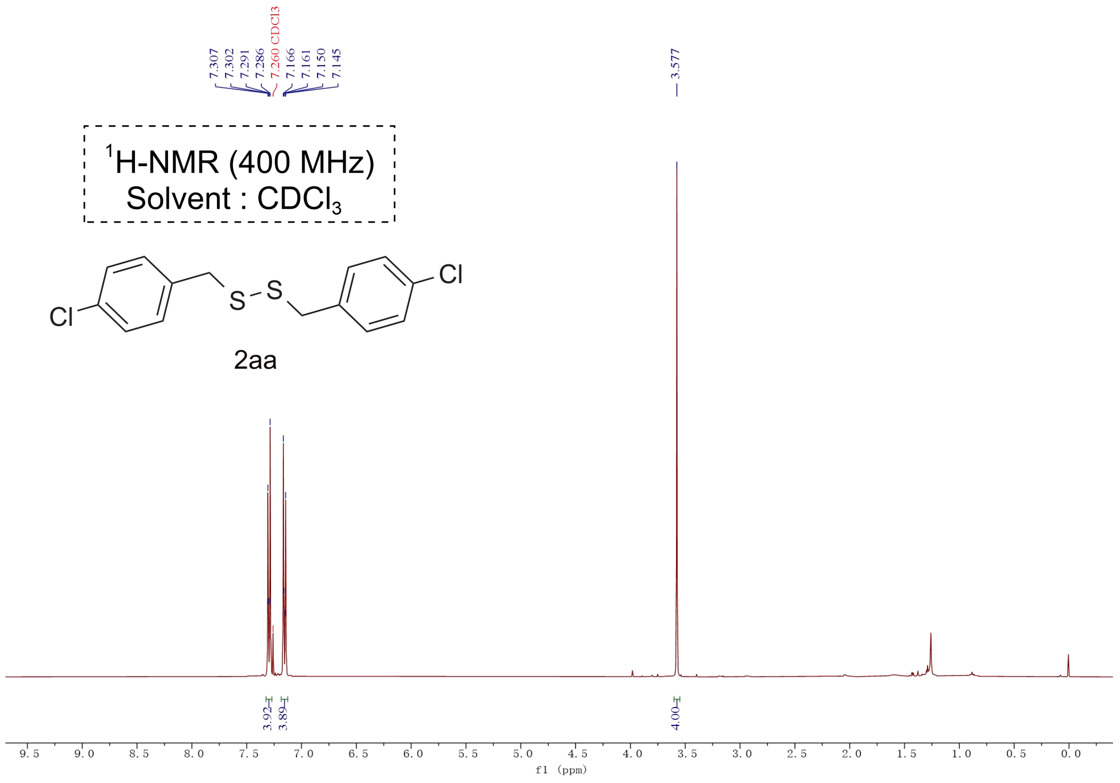

7.307
7.302
7.291
7.286
7.260 CDCl3
7.166
7.161
7.150
7.145
3.577
^{1}H-NMR (400 MHz)
Solvent : $CDCl_3$
Cl
S
S
Cl
2aa
3.92
3.89
4.00
9.5 9.0 8.5 8.0 7.5 7.0 6.5 6.0 5.5 5.0 4.5 4.0 3.5 3.0 2.5 2.0 1.5 1.0 0.5 0.0
f1 (ppm)

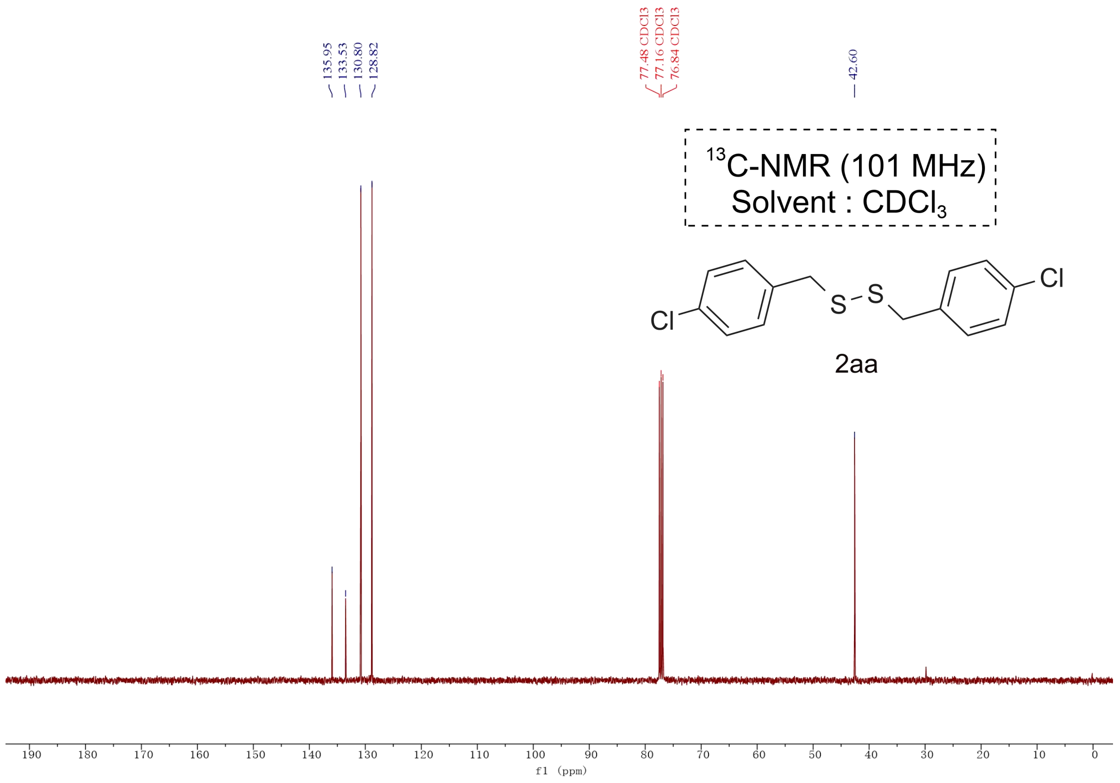

135.95
133.53
130.80
128.82
77.48 CDCl3
77.16 CDCl3
76.84 CDCl3
42.60
^{13}C-NMR (101 MHz)
Solvent : $CDCl_3$
Cl
S
S
Cl
2aa
190 180 170 160 150 140 130 120 110 100 90 80 70 60 50 40 30 20 10 0
f1 (ppm)

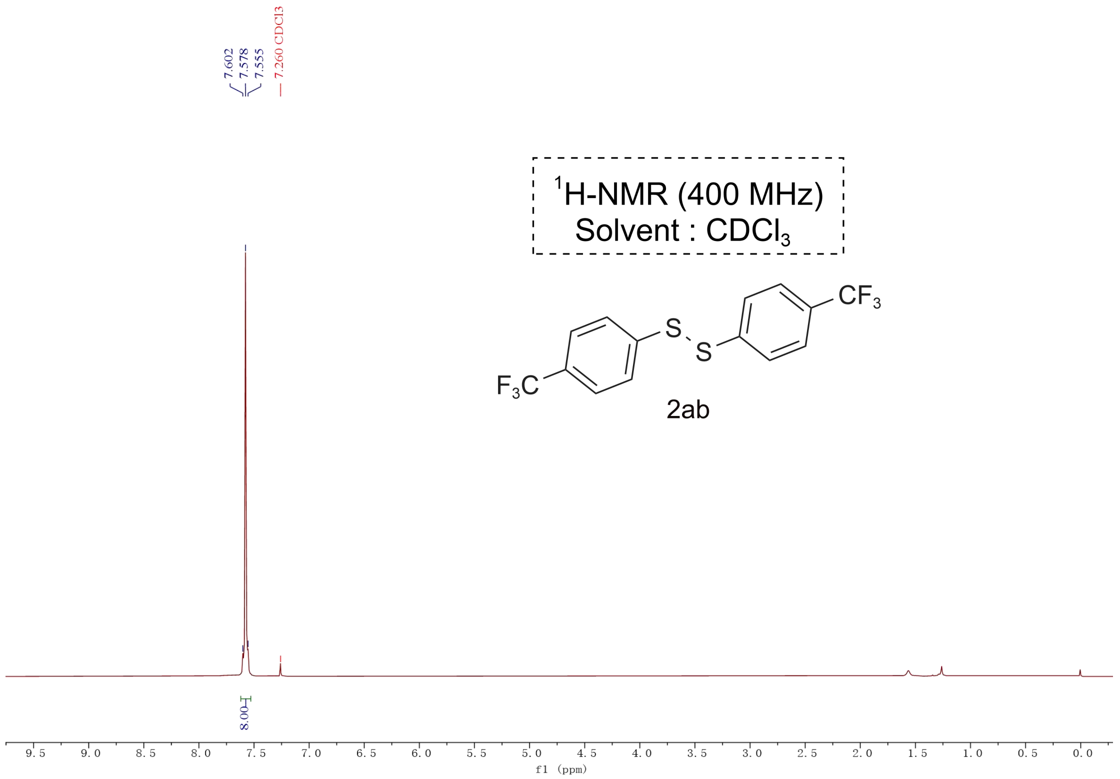
7.602
7.578
7.555
7.260 CDCl3
1H-NMR (400 MHz)
Solvent : CDCl3
CF3
S
S
F3C
2ab
8.00
9.5 9.0 8.5 8.0 7.5 7.0 6.5 6.0 5.5 5.0 4.5 4.0 3.5 3.0 2.5 2.0 1.5 1.0 0.5 0.0
f1 (ppm)

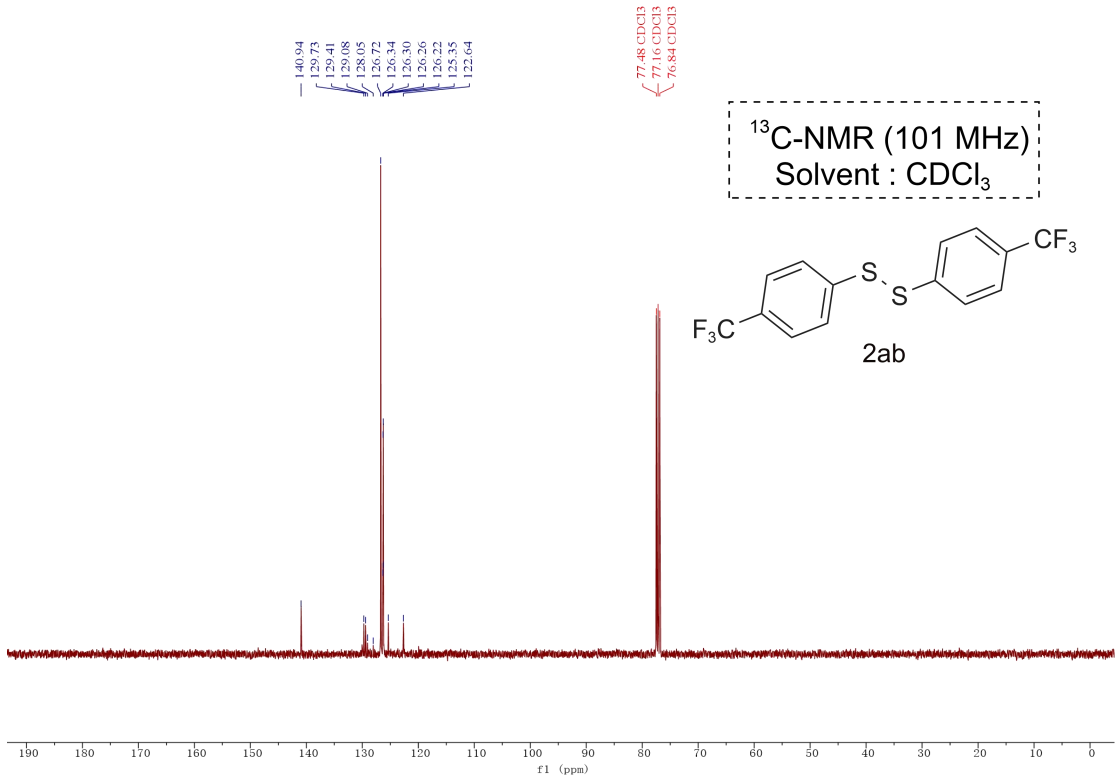
140.94
129.73
129.41
129.08
128.05
126.72
126.34
126.30
126.26
126.22
125.35
122.64
77.48 CDCl3
77.16 CDCl3
76.84 CDCl3
13C-NMR (101 MHz)
Solvent : CDCl3
CF3
S
S
F3C
2ab
190 180 170 160 150 140 130 120 110 100 90 80 70 60 50 40 30 20 10 0
f1 (ppm)

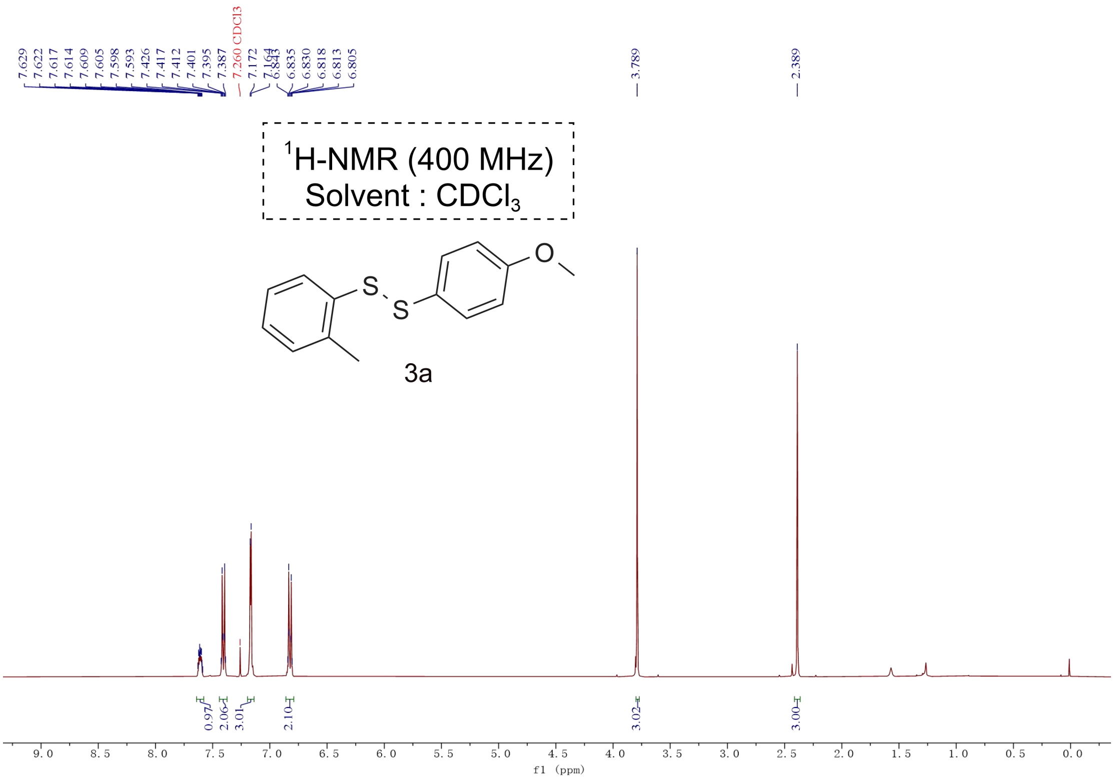
7.629
7.622
7.617
7.614
7.609
7.605
7.598
7.593
7.426
7.417
7.412
7.401
7.395
7.387
7.260 CDCl3
7.172
7.164
6.843
6.835
6.830
6.818
6.813
6.805
3.789
2.389
¹H-NMR (400 MHz)
Solvent : CDCl₃
S
S
O
3a
0.97
2.06
3.01
2.10
3.02
3.00
f1 (ppm)

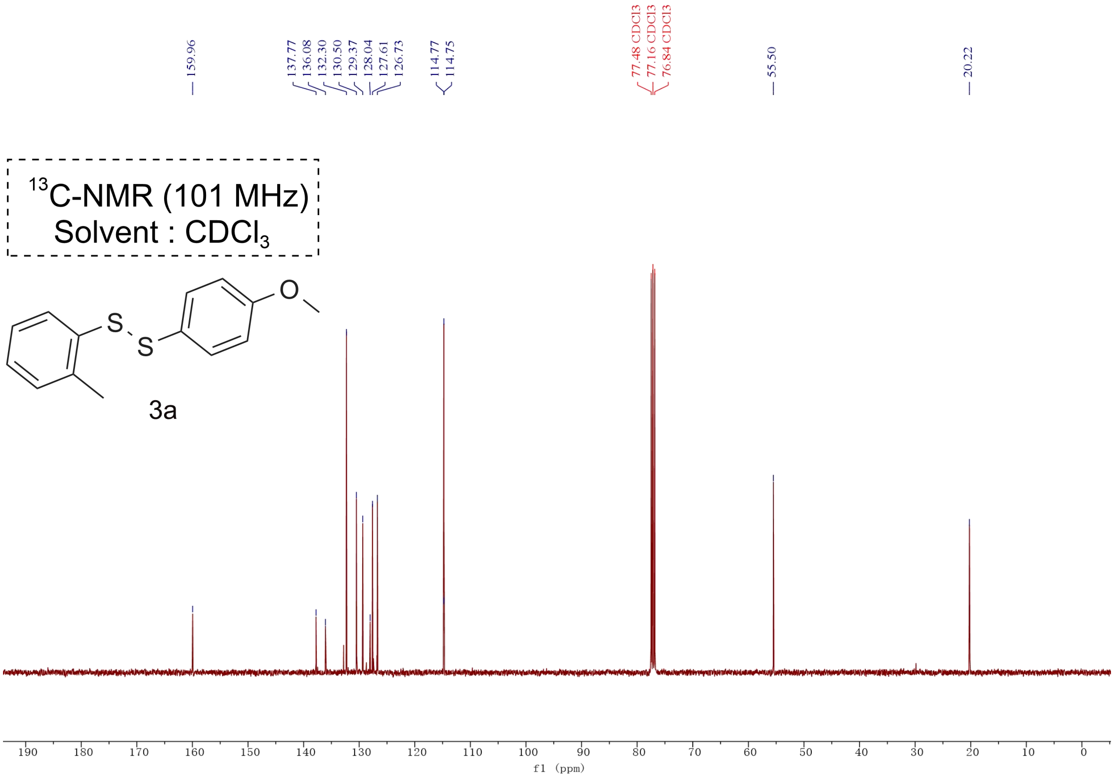
159.96
137.77
136.08
132.30
130.50
129.37
128.04
127.61
126.73
114.77
114.75
77.48 CDCl3
77.16 CDCl3
76.84 CDCl3
55.50
20.22
¹³C-NMR (101 MHz)
Solvent : CDCl₃
S
S
O
3a
f1 (ppm)

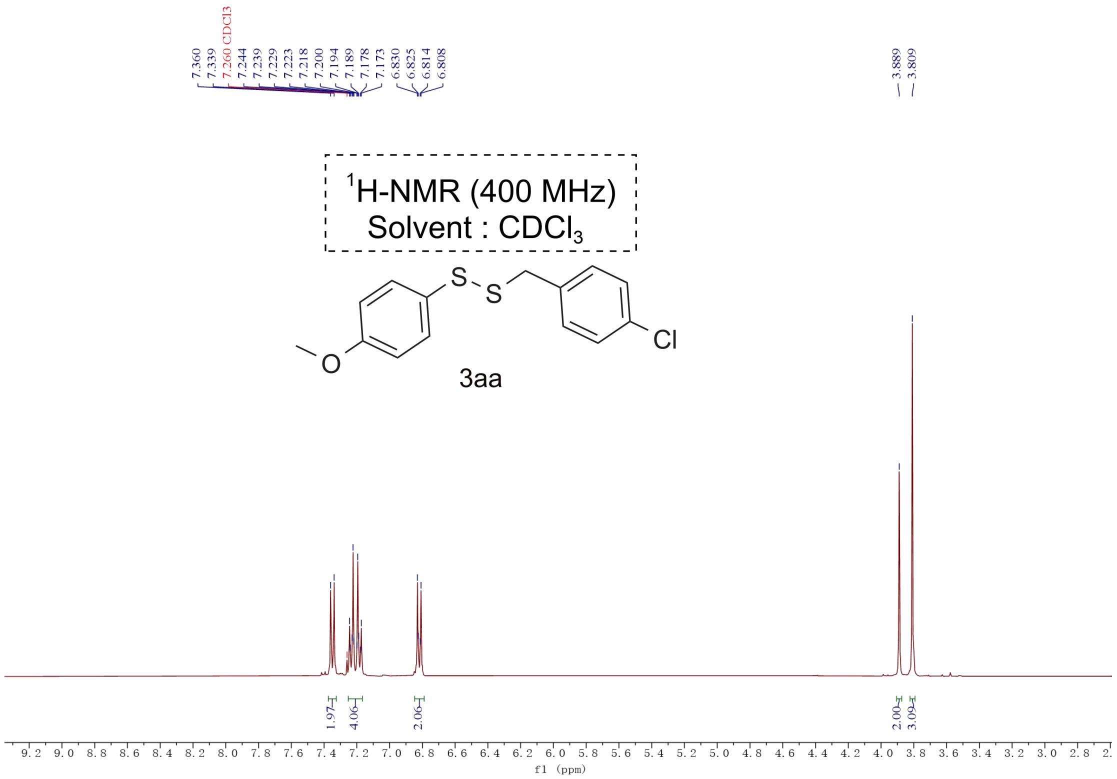
7.360
7.339
7.260 CDCl3
7.244
7.239
7.229
7.223
7.218
7.200
7.194
7.189
7.178
7.173
6.830
6.825
6.814
6.808
3.889
3.809
1H-NMR (400 MHz)
Solvent : CDCl3
S
S
O
Cl
3aa
1.97
4.06
2.06
2.00
3.09
f1 (ppm)

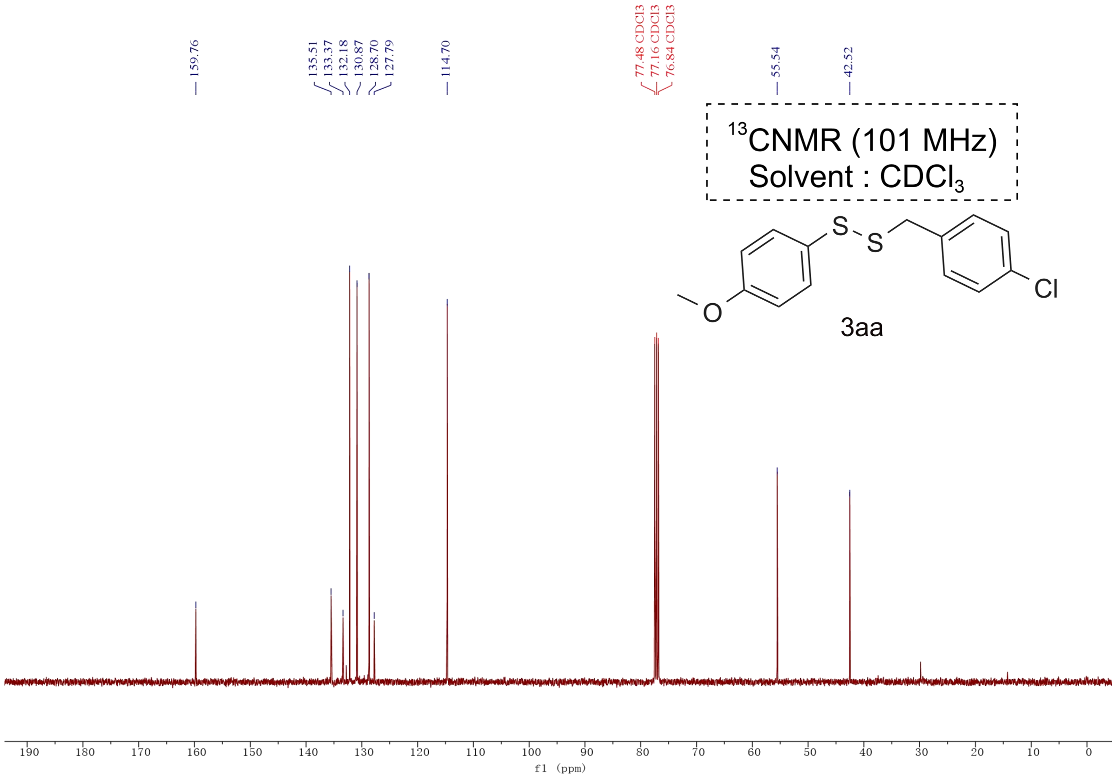
159.76
135.51
133.37
132.18
130.87
128.70
127.79
114.70
77.48 CDCl3
77.16 CDCl3
76.84 CDCl3
55.54
42.52
13CNMR (101 MHz)
Solvent : CDCl3
S
S
O
Cl
3aa
f1 (ppm)

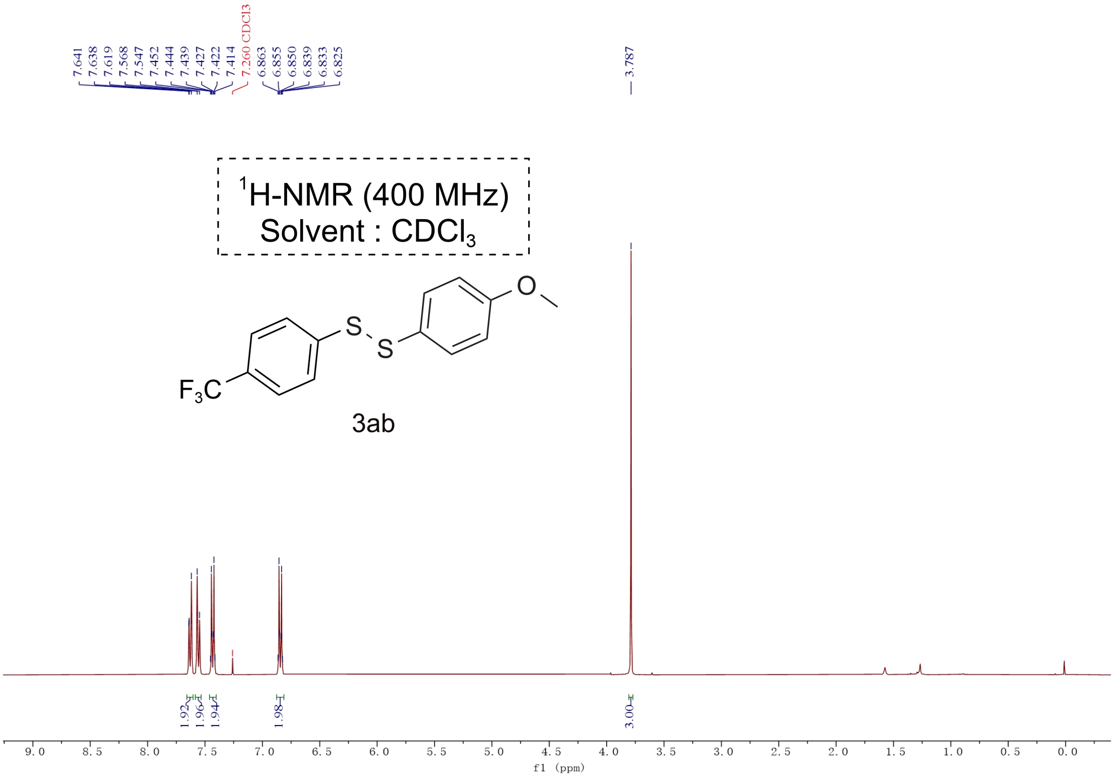
7.641
7.638
7.619
7.568
7.547
7.452
7.444
7.439
7.427
7.422
7.414
7.260 CDCl3
6.863
6.855
6.850
6.839
6.833
6.825
3.787
1H-NMR (400 MHz)
Solvent : CDCl3
O
S
S
F3C
3ab
1.92
1.96
1.94
1.98
3.00
f1 (ppm)

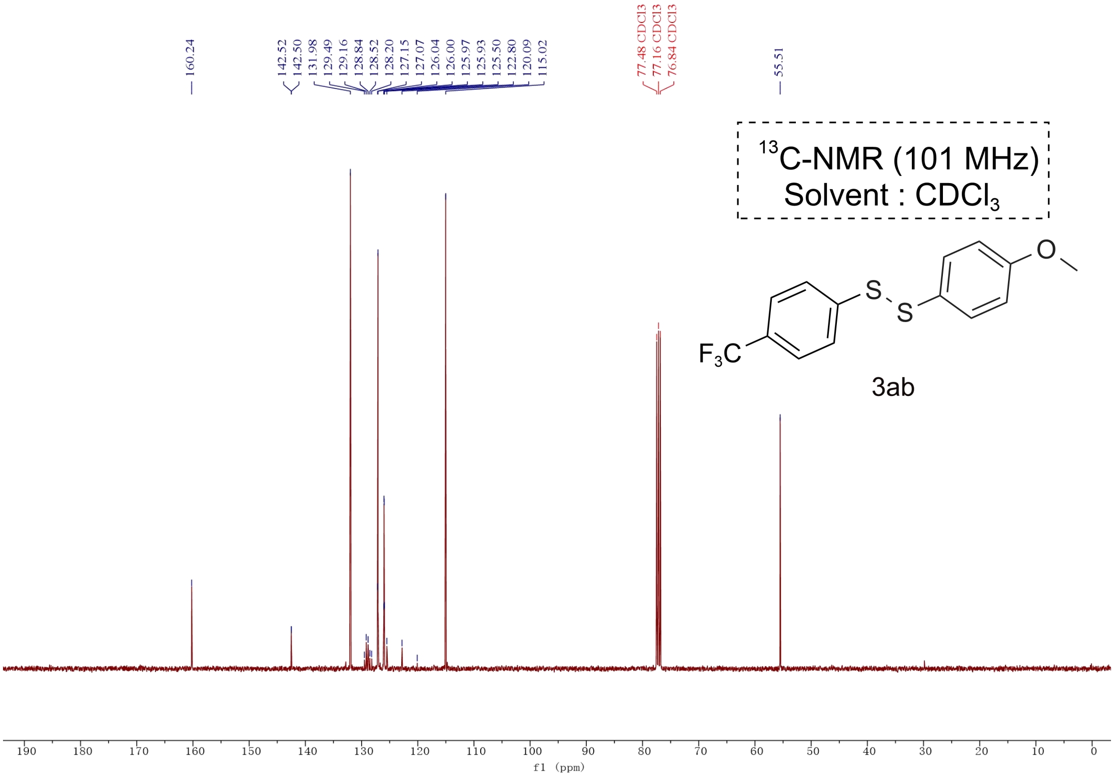
160.24
142.52
142.50
131.98
129.49
129.16
128.84
128.52
128.20
127.15
127.07
126.04
126.00
125.97
125.93
125.50
122.80
120.09
115.02
77.48 CDCl3
77.16 CDCl3
76.84 CDCl3
55.51
13C-NMR (101 MHz)
Solvent : CDCl3
O
S
S
F3C
3ab
f1 (ppm)
